# Selective Native N_(in)_–H Bond Activation in Peptides with Metallaphotocatalysis

**DOI:** 10.1021/jacsau.5c00119

**Published:** 2025-03-28

**Authors:** José A. C. Delgado, Jéssica C. Amaral, Paula S. Penteado, Antonio G. Ferreira, Maria Fátima G. F. da Silva, Burkhard König, Márcio W. Paixão

**Affiliations:** † Laboratory for Sustainable Organic Synthesis and Catalysis, Department of Chemistry, Federal University of São Carlos − UFSCar, Rodovia Washington Luís, km 235 - SP-310, São Carlos, São Paulo 13565-905, Brazil; ϕ Department of Plant Pathology and Nematology, University of São Paulo (USP)/Luiz de Queiroz College of Agriculture (ESALQ), Av. Pádua Dias, 11, Piracicaba, São Paulo 13418-900, Brazil; ¶ Department of Chemistry, Federal University of São Carlos − UFSCar, Rodovia Washington Luís, km 235 - SP-310, São Carlos, São Paulo 13565-905, Brazil; § Institute of Organic Chemistry, University of Regensburg, 93040 Regensburg, Germany

**Keywords:** glucagon-like peptide-1, Flufirvitide-3, metallaphotocatalysis, solid-phase, chemoselective, orthogonal, C(sp^2^)-N cross-coupling

## Abstract

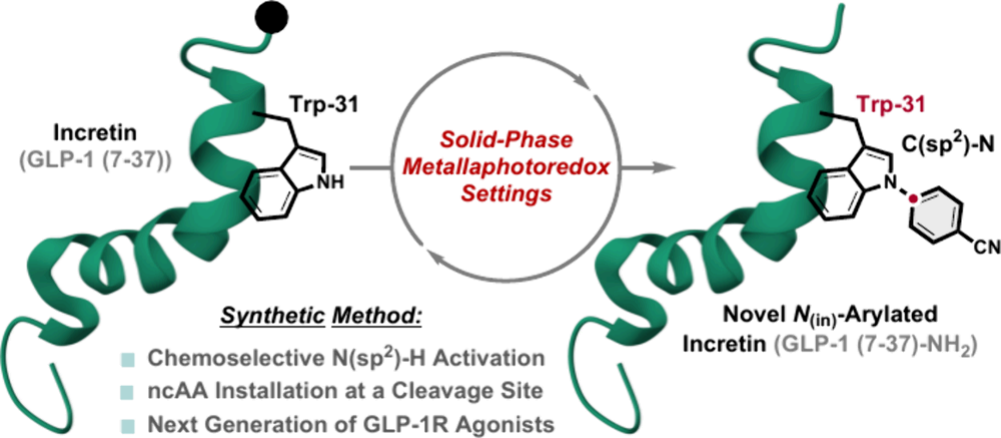

The development of chemical methods enabling site-selective incorporation of noncanonical amino acids into peptide backbones with precise functional tailoring remains a critical challenge. Particularly compelling is the use of underexplored endogenous amino acid hotspots, such as the *N*_(in)_ of tryptophan, as versatile anchors for diversification. Herein, we report a chemoselective N(sp^2^)–H bond activation strategy targeting native tryptophan residues within peptide frameworks, exemplified by GLP-1 (7–37), using nickel metallaphotocatalysis under postsynthetic solid-phase conditions. This selective *N*_(in)_-arylation reaction proceeds efficiently within 3 h of light irradiation in highly functionalized heterogeneous environments, employing minimal excesses of electrophile and base, alongside catalytic quantities of nickel, ligand, and photocatalyst. The method affords homogeneous peptide products with high chemoselectivity and operational simplicity. We envision that this strategy could contribute to advancing the design of the next-generation long-acting class II G protein-coupled receptor agonist therapeutics.

## Introduction

Endogenous bioactive peptides (*endo*BPs) are naturally occurring signaling molecules derived from proteins secreted in various cells and glands. Typically composed of fewer than 50 amino acid residues, these peptides are activated throughout processes such as protein folding, unfolding, and enzymatic cleavage.^1−5^*Endo*BPs are characterized by their high selectivity and specificity, enabling them to bind target receptors with minimal off-target interactions and thereby trigger precise intracellular signaling pathways. This remarkable selectivity and specificity are the outcomes of evolutionary refinement, shaped over millions of years to achieve complementary structural and functional diversity.^6^ As a result, *endo*BPs have garnered significant attention as promising therapeutic candidates, representing one of the hottest drug discovery and development themes among the leading global pharmaceutical companies.^7,8^

**Scheme 1:**
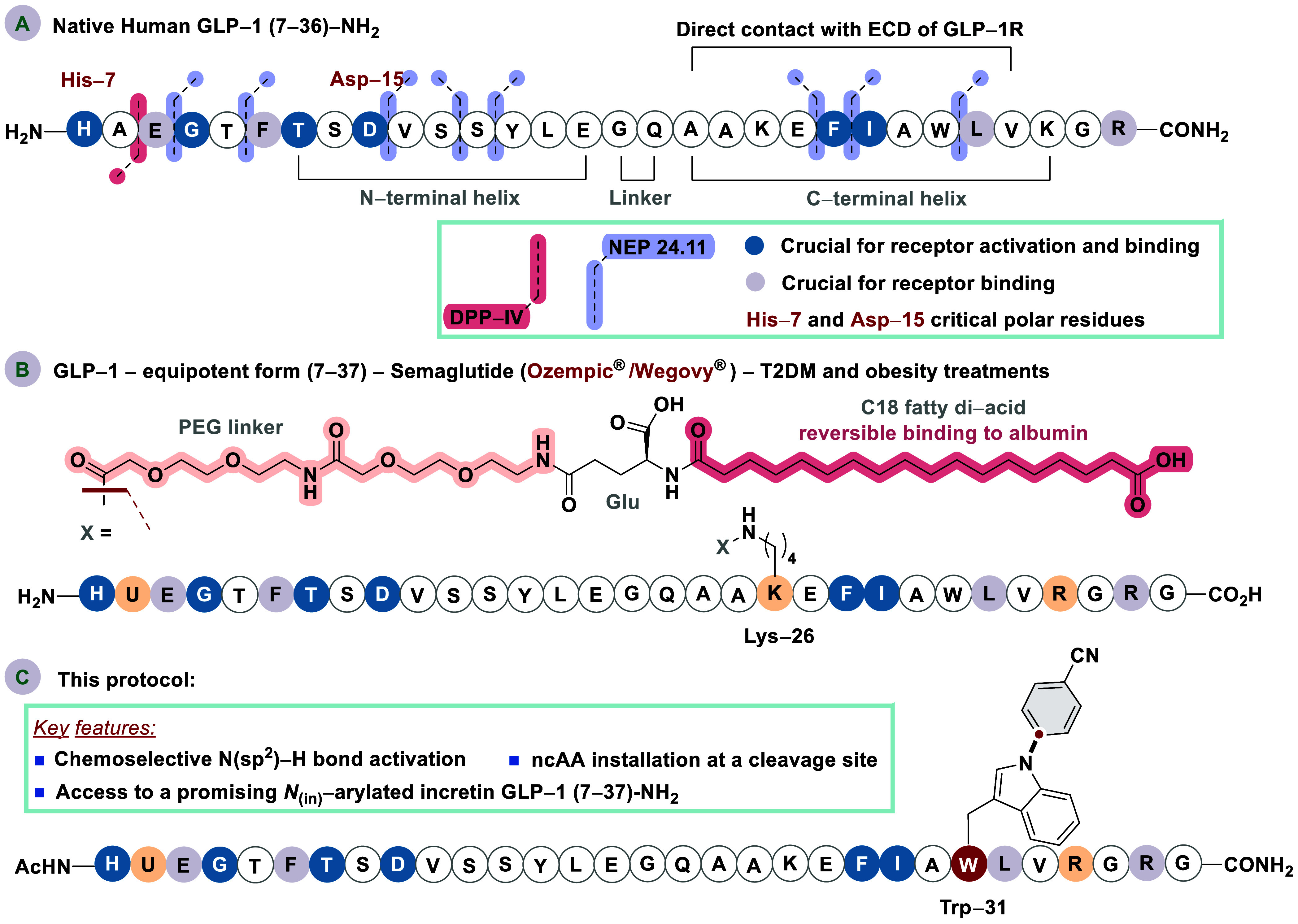
(A) Representative Features of Native Human Incretin GLP-1 (7-36)–NH_2_, (B) Structure and Features of Semaglutide Peptide (GLP-1-Equipotent Form (7–37)), and (C) This Protocol: Chemoselective and Irreversible *N*_(in)_-Arylation of Incretin GLP-1 (7–37)–NH_2_

As an outstanding naturally occurring bioactive peptide, the native human glucagon-like peptide-1 (GLP-1) encompassing incretins GLP-1 (7–36)–NH_2_ and GLP-1 (7–37) is a metabolic hormone secreted by enteroendocrine cells in response to nutrient intake.^9−11^ GLP-1 plays a pivotal role in regulating glucose homeostasis and exerts its function throughout an α-helix-mediated interaction (Scheme 1, A). Specifically, residues comprising Ala-24 to Val-33 engage with the extracellular domain (ECD) of the GLP-1 receptor (GLP-1R), a class II G protein-coupled receptor (GPCR).^9^ Alanine scanning in conjunction with other structure–activity relationship (SAR) studies have identified essential residues critical for receptor binding and activation.^9^ Studies have demonstrated that GLP-1 has a remarkably short circulation half-life time in human plasma (*ca*., 2 min) due to rapid enzymatic proteolysis by dipeptidyl peptidase IV (DPP-IV) and neutral endopeptidase 24.11 (NEP 24.11)—this intrinsic instability hampers therapeutic effectiveness in its native form.^11,12^

To cope with such a limitation, covalent modifications to the GLP-1 backbone have been explored, focusing on the strategic incorporation of customized side-chain warheads designed for tailored functionality. As a result, a series of long-acting GLP-1R agonists featuring superior pharmacokinetic and pharmacodynamic profiles have been developed, providing solutions to the intrinsic instability of native GLP-1 and unlocking its full therapeutical potential.^13−15^

Semaglutide peptide, an exogenous GLP-1 (7–37) analogue, is arguably the most exceptional GLP-1R agonist hitherto developed (Scheme 1, B). Its subcutaneously administered formulations—marketed under the registered trademarks Ozempic and Wegovy by Novo Nordisk—have revolutionized the therapeutic landscape for type-2 diabetes mellitus (T2DM) and obesity.^12,15−17^ Certainly, the strategic incorporation of tailored side chains into the native GLP-1 backbone significantly enhances its pharmacological attributes (*e.g*., semaglutide plasma half-life *ca*., 165 h).^10^

From a synthetic outlook, methodologies designed to precisely incorporate noncanonical amino acids into a peptide backbone *via* the straightforward edition of specific native amino acid side chains in late-stage scenarios have garnered significant attention across diverse disciplines.^18−21^ Such approaches expedite the construction of exogenous bioactive peptides (*exo*BPs) by precluding time- and resource-intensive *de novo* synthesis of individual analogs. Likewise, they provide a valuable platform for in-depth investigations of SAR studies. Nonetheless, achieving site-selective functionalization within polypeptides is conspicuously fraught with challenges. Among amino acid residues, cysteine (C) has emerged as the preferred target owing to its high reactive profile, low relative abundance (around 1%), and ease of engineered incorporation.^22−25^ Conversely, other residues, such as the *N*_(in)_ of tryptophan (W), pose significant challenges, rendering them less suitable for conventional functionalization strategies.^26^

We recently introduced nickel metallaphotocatalysis in solid-phase peptide synthesis (SPPS) to enable a postsynthetic orthogonal C(sp^2^)–N cross-coupling reaction targeting biologically relevant peptides.^27^ For the first time, this approach allowed the selective activation of the native N(sp^2^)–H bond within the indole unit of the tryptophan residue, discriminating it from other nucleophilic N–H hotspots. Anticipating a synergistic dual photoredox/nickel catalytic mode^28−31^ under resin-supported peptide heterogeneous settings was essential for harnessing ubiquitous N–H bonds as functional handles. The catalytic system exhibits excellent regioselectivity, achieving precise arylation at the *N*_(in)_ atom to generate homogeneous peptide conjugates. Moreover, the methodology tolerates a broad range of bromide coupling partners, including those embedded within pharmacologically active molecules, affinity tags, labeling groups, and bioconjugation handles, underscoring its versatility and potential for diverse applications.

## Overview of the Procedure

The synthetic method reported here achieves chemoselective N(sp^2^)–H bond activation, enabling irreversible arylation of structural complexes and pharmacologically significant tryptophan-containing peptides. In particular, we exemplify the operational simplicity, robustness, and relevance of our protocol for incorporating tailored functions into the exogenous incretin GLP-1 (7–37) backbone (*i.e*., Ac-**HUEGTFTSDVSSYLEGQAAKEFIAWLVRGRG**-NH_2_ sequence) (Scheme 1, C). Considering the growing demand for innovative therapeutic agents to address diabetes and related metabolic disorders, this solid-phase metallaphotoredox methodology is poised to open new opportunities to facilitate the engineering of the next generation of long-acting GLP-1R agonists based on incretins GLP-1, gut peptides, and GLP-1 homologous structural cores (*e.g*., Exendin-4).^10^ The procedure comprises three distinct steps: (i) the solid-phase assembly of the target-specific peptides using the orthogonal Fmoc/^*t*^Bu chemistry workflow; (ii) the subjection of the resulting resin’s beads loaded with crude peptidic material to nickel metallaphotoredox postsynthetic configurations; and (iii) the release of entirely unprotected *N*_(in)_-arylated crude peptides from the resin upon acidic treatment. Notably, the cross-coupling reaction proceeds smoothly under highly functionalized heterogeneous environments, using molar equivalent excesses of the electrophile and base but catalytic amounts of nickel, ligand, and photocatalyst, generating singly arylated conjugates.

## Applications of the Method

By employing an aryl bromide linker-based strategy, various relevant scaffolds can be precisely designed and cross-linked onto tryptophan-containing peptides. These scaffolds include drug molecules, bioconjugation linchpins, fluorescent and affinity tags, fatty acids, and peptides.^27^ In addition to enabling the incorporation of noncanonical structural features into side chains, thereby enhancing resistance to proteolysis, this methodology provides a versatile toolkit for investigating protein–ligand interactions and protein’s three-dimensional structure. Notably, the experimental setup described here is compatible with any tryptophan-containing peptide,^27^ including those with challenging long sequences that are difficult to synthesize manually or automatically. This compatibility is exemplified by the chemoselective and irreversible *N*_(in)_-arylation of the full-length 31-mer incretin GLP-1 (7–37) and 16-mer Flufirvitide-3 analogous peptides, showcasing the robustness and broad applicability of the method.

## Experimental Design

**Scheme 2:**
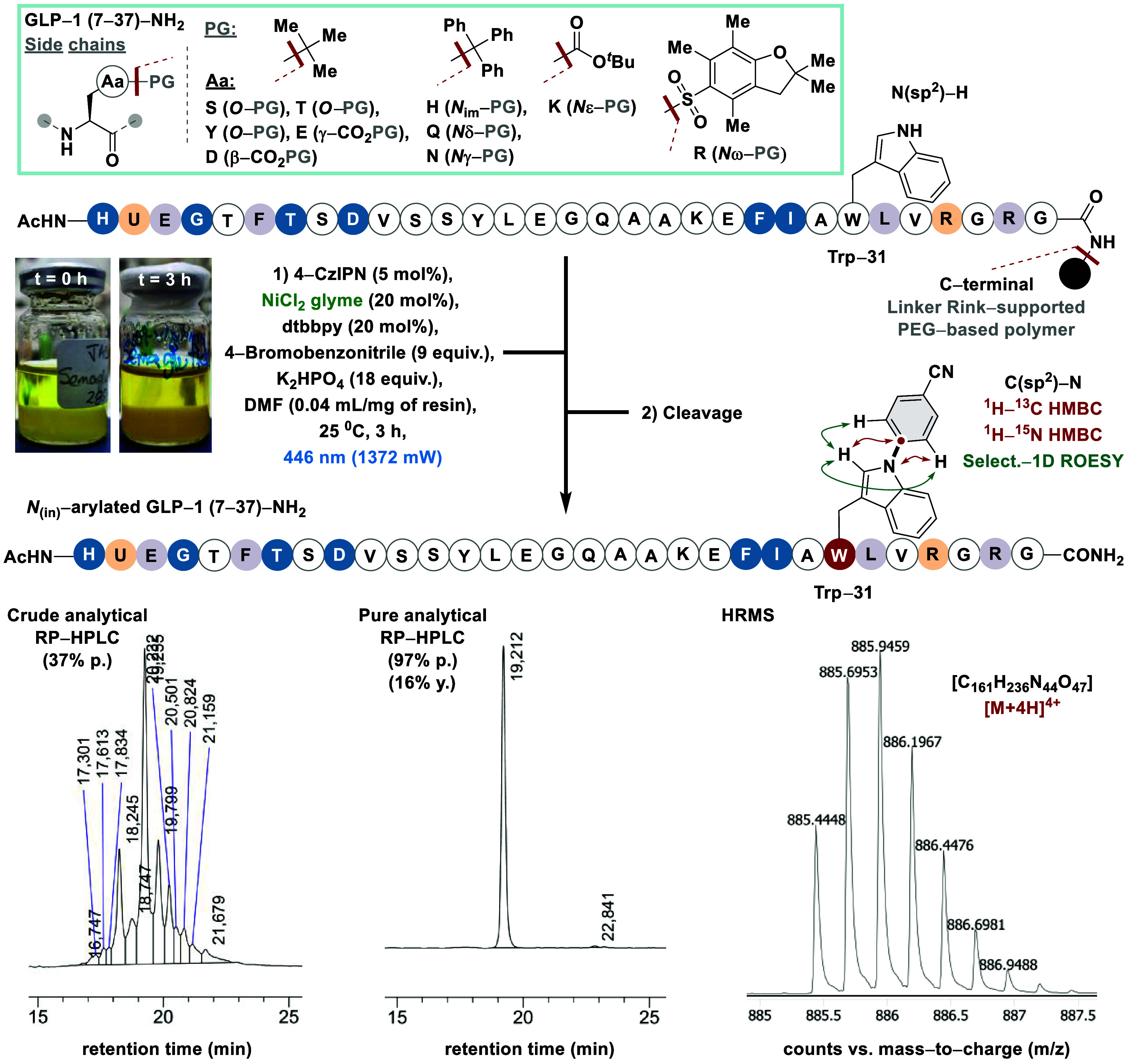
Side-Selective Solid-Phase *N*_(in)_-Arylation of Incretin GLP-1 (7–37) with Nickel Metallaphotocatalysis, Reaction conditions: Peptide (45 μmol), 4-bromobenzonitrile (9 equiv), 4-CzIPN (5 mol %), NiCl_2_·glyme (20 mol %), dtbbpy (20 mol %), K_2_HPO_4_ (18 equiv) in DMF (4 mL). In brackets, purity (p.) (calculated by analytical RP-HPLC analysis of the crude and purified peptide material) and the isolated yield (y.) after (2*n*+1 or 2*n*+2 for the acetylated sequences, *n* = number of amino acids) LLS. In the case of acetylated peptides, the reaction was carried out using 1 equiv of l-alanine methyl ester hydrochloride as additive.

Our protocol employs nickel metallaphotocatalysis to achieve chemoselective and irreversible *N*_(in)_-arylation of the native tryptophan unit in oligopeptides (Scheme 2). Akin to cysteine, tryptophan exhibits several distinctive features that make it an ideal target for site-specific functionalizations. These include its scarcity among proteinogenic amino acids (*i.e*., a frequency of *ca*., 1.7%), its status as the largest amino acid (*i.e*., molecular formula of C_11_H_12_N_2_O_2_ and molecular weight of 204.22 g/mol), and its low absorptive cross-section (*i.e*., molar absorptivity, *ε* ≈ 4500, 500 M^–1^ cm^–1^, 290, 300 nm, respectively).^32−34^ These unique physicochemical properties are particularly well-suited for leveraging photoinduced redox chemistry, enabling precise “on- and off-side chain” editing of peptides to access homogeneous conjugates accurately.^35^ Thus, as a proof of concept for this synthetic method, we aimed to target a specific and biologically relevant class of naturally occurring peptide-like hormone GPCR agonist displaying a single tryptophan residue.

We began our study by elongating the unmodified Lys-26 peptidyl core of Semaglutide through a manual SPPS protocol on the H-Rink amide ChemMatrix resin (Scheme 2). The selection for a polyethylene glycol-based resin was driven by its superior swelling properties, which facilitate the accommodation of long-length sequences,^36^ although polystyrene-based resins, such as Rink-MBHA and Wang, are also amenable.^27^ Stepwise amide couplings were carefully monitored until completion by the Kaiser qualitative test. Given that our protocol relies on a metallaphotocatalytic procedure performed on a solid support, the method of peptide assembly, whether manual or automated using commercially available synthesizers, does not critically impact reaction outcomes or reproducibility. However, we recommend evaluating the final purity of each prepared sequence on a case-by-case basis. Amino acid building blocks were used with standard side chain protecting groups according to the Fmoc/^*t*^Bu orthogonality, except for tryptophan, which was implemented in its *N*_(in)_-unprotected form.

For the GLP-1 family, which activates class II GPCRs, the histidine (H) residue positioned at the N-terminal is highly preserved and necessary for receptor binding and activation.^9^ Modifying the His-7 with *N*_(α)_-acetyl reduced its potency; however, such analogs retain a potent insulinotropic effect in vitro.^37,38^ Consequently, we opted to cap the N-terminus with the acetyl group to enhance experimental safety handling (Scheme 2). Moreover, our protocol’s metallaphotoredox reactivity when challenging N-terminal acetylated sequences remains unthwarted whenever performing additivities with l-alanine methyl ester hydrochloride (1 equiv).

Subjecting the solid-supported preassembled GLP-1 (7–37) analogue to our metallaphotoredox setup, followed by cleavage, afforded a single-site *N*_(in)_-aryl conjugate in 37% purity, as determined by RP-HPLC analysis of the crude peptide (Scheme 2). Analysis of the crude RP-HPLC and LC-MS profiles revealed the exclusive conversion of the starting peptide material to the *N*_(in)_-arylated conjugate (57% conversion), indicating product homogeneity (see SI for details). Achieving a conversion rate slightly exceeding 50% is considered highly satisfactory for this methodology. Nonetheless, we strongly recommend performing the metallaphotoredox step over more than one round to increase product conversion on a case-by-case basis. RP-HPLC purification afforded the *N*_(in)_-arylated GLP-1 (7–37)–NH_2_ analog in a 97% purity and 16% isolated yield after 64 steps of longest linear sequence (LLS) (Scheme 2). Comprehensive characterization using UHPLC-QTOF/HRMS analysis and diligent NMR experiments (*e.g*., ^1^H–^13^C and ^1^H–^15^N HMBC and selective 1D-ROESY) unequivocally corroborated one aryl unit increment in the conjugate and the chemoselectivity at *N*_(in)_ of the Trp-31 residue (Scheme 2) (see SI for complete insights).

Crucially, insights into the crystal structure of the unacetylated Lys-26 Semaglutide peptide backbone in complex with the GLP-1R ECD revealed that the hydrophobic association between the Trp-31 and Arg-36 residues with Glu-68 of the GLP-1R is one of the predominant noncovalent interactions on the ligand–receptor interface.^17^ In the same vein, peptides featuring substantial hydrophobic C-terminal noncanonical biphenyl side chains closely mimic the C-terminal 21-mer sequence of GLP-1, as demonstrated by the development of structurally optimized 11-mer GLP-1R agonists.^39^ Building on these insights, the synthetic methodology reported herein establishes a robust platform to augment GLP-1 C-terminal hydrophobicity through *N*_(in)_-arylation of the Trp-31 residue and therefore sheds light on GLP-1R pharmacology.

Furthermore, this method was also successfully applied to the *N*_(in)_-edition of the Trp unit of Flufirvitide-3—an antiviral drug that inhibits the hemagglutinin (HA) spike protein of influenza viruses.^40,41^ This 16-mer peptide once subjected to our solid-phase C(sp^2^)–N cross-coupling protocol yielded the Flufirvitide-3 analog with a 65% purity after 33 steps (LLS) (see SI for complete details). These results emphasize the applicability of this synthetic method for accessing a wide variety of decorated naturally occurring bioactive peptides as potential new therapeutics.

## Procedure for Arylating Incretin GLP-1 (7–37)

1. Assemble the peptide sequence (Ac-**HUEGTFTSDVSSYLEGQAAKEFIAWLVRGRG**-NH_2_) following Supporting Information, general procedure A, Note 1.
2. Based on the resin’s substitution value (0.45 mmol/g), prepare a 6 mL crimp headspace vial (photoreactor vessel) with 0.45 μmol of crude peptide GLP-1 (7–37), which remains resin-supported (theoretical mass for dry peptide + resin = 329 mg, experimental weighted mass for dry peptide + resin = 284 mg, Note 2).
3. Weigh 4-bromobenzonitrile (74 mg, 9 equiv), K_2_HPO_4_ (141 mg, 18 equiv), and l-alanine methyl ester hydrochloride (6 mg, 1 equiv) and transfer the reagents into the photoreactor vessel.
4. Separately, charge three individual flask vials with 4-CzIPN (17.8 mg) (stock solution 1); NiCl_2_-Glyme (19.8 mg) (stock solution 2); and dtbbpy (24.2 mg) (stock solution 3). To each vial add 1 mL of DMF and sonicate until complete homogeneity. Critical step: Use the stock solutions immediately after preparation to ensure optimal performance.
5. Add to the photoreactor vessel 3.7 mL of DMF.
6. Sequentially, transfer 100 μL aliquots of each stock solution (1, 2, and 3) into the photoreactor vessel.
7. Seal the 6 mL photoreactor vessel with an aluminum crimp cap.
8. Store the sealed photoreactor vessel in a refrigerator overnight. Critical step: The resin, with peptide attached, is weighted in its dry form and must swell properly before initiating the photochemical reaction. While storage temperature is not critical (−18 or 4 °C are both acceptable), maintaining a lower temperature is generally preferred for optimal results.
9. Using a Schlenk line and hypodermic syringe needles, bubble N_2_ through the heterogeneous solution for 15 min. Critical step: Oxygen must be removed this way; freeze–pump–thaw cycles are not suitable.
10. Wrap the aluminum crimp cap of the photoreactor vessel with parafilm to ensure a tight seal. Place the vial into a metal cooling block maintained at 25 °C, positioned on top of a 446 nm LED (20 V, 1372 mW) array plate. Ensure photochemical setup device is fixed on an orbital shaker platform.
11. Shake the reaction vial at 249.8 r.p.m. while irradiating it for 3 h, Note 3.
12. Carefully remove the aluminum crimp cap from the photoreactor vial and transfer the heterogeneous reaction mixture completely to an SPPS reactor vessel (*e*.*g*., a 5 mL disposable graduated polypropylene syringe equipped with a polyethylene frit).
13. Apply vacuum to the reaction vessel and rinse the resin with small portions of MiliQ water until no white solid (excess K_2_HPO_4_) remains.
14. Wash the resin’s beads sequentially following standard SPPS procedure: DMF (3 × 1 min); MeOH (3 × 1 min); and Et_2_O (3 × 1 min).
15. Place the SPPS reaction vessel containing the resin on a glass vacuum desiccator and allow it to dry for 1–3 days.
16. Cleave the peptide crude material from the resin beads according to the SI, general procedure B.
17. Analyze the reaction outcome by RP-HPLC (1 mg of crude lyophilized peptide dissolved to 1 mL of MiliQ-water with 0.1% of TFA containing *ca*., 40% of ACN and filtered through a disposable syringe filter, 22 μm) and LC-MS (50-μL aliquot of the RP-HPLC sample made up to 1 mL with MS-grade solvent can be directly used for LC-MS analysis). The calculated mass for the *N*_(in)_-arylated GLP-1 (7–37)–NH_2_ peptide is 1180.2549 Da for the [M+3H]^3+^ ion and 885.4430 Da for the [M+4H]^4+^ ion. The observed masses for the corresponding ions were 1180.2537 Da, diff: −1.04 ppm and 885.4431, diff: 0.1, respectively.
18. Purify the crude reaction mixture to a purity level of up to ≥95% using analytical RP-HPLC over multiple injections (purified peak: retention time of 19.26 min; autosampler configurations: injection volume: 50 μL; excess volume: 10 μL), Note 4.
19. Lyophilize the purified sample immediately to obtain the desired compound as a white solid. Critical step: Immediate lyophilization is recommended to prevent background reaction, particularly for sequences containing cysteine residues, Note 5.
20. Characterize the peptide sequence (Ac-**HUEGTFTSDVSSYLEGQAAKEFIAW(4-BrPh)LVRGRG**-NH_2_) unambiguously by combining RP-HPLC, UHPLC-QTOF/HRMS, and NMR techniques.

Note 1: Fmoc/^*t*^Bu SPPS was performed manually; however, it could also be done automatically. The following protecting groups are recommended for the side chains: **Trt** for Asn, Gln, and His; **Boc** for Lys; ^***t***^**Bu** for Ser, Thr, Asp, Glu, and Tyr; and **Pbf** for Arg. For Trp residues, no side-chain protection is required. Peptide couplings are best carried out using DIC/HOBt activation for optimal efficiency and yield.

Note 2: For reproducible results at laboratory scales, it is recommended to initiate synthesis with a resin mass of ≥400 mg. Upon completion of the synthesis, the final dry solid material was divided into equal portions based on resin substitution and experiment scale settings. Important Note: The mass of “peptide + resin” consumed during each Kaiser test procedure is considered negligible and does not significantly impact the overall yield or reproducibility.

Note 3: If the reaction shows poor conversion (according to the analysis in step 17), then it is recommended to repeat steps 2–14 on the same sample for improved results.

Note 4: Purification was performed by using an analytical HPLC (reverse phase) apparatus equipped with a 50 μL loop. For each 50 μL injection, 10 μL was excluded from purification (*e*.*g*., purification of 52.3 mg of crude peptide (*N*_(in)_-arylated GLP-1 (7–37)–NH_2_) dissolved in 3 mL of DMSO over *ca*., 56 injections afforded 14 mg of ≥95% pure material).

Note 5: Immediate lyophilization is required to minimize undesired premature oxidation when handling Met- and Cys-containing sequences. Met: sulfoxide [M + 16 units]; Cys: intramolecular disulfide bridge [M – 2 units], intermolecular disulfide bridge [2 M – 2 units].

## Supporting Information Available

General procedures; setup photographs; RP-HPLC data; LC-MS data; LC-MS/MS data; and HRMS and NMR spectral data for peptides (PDF)

## ABBREVIATIONS

*endo*BP: endogenous bioactive peptide
GLP-1: glucagon-like peptide-1
ECD: extracellular domain
GLP-1R: glucagon-like peptide-1 receptor
GPCR: G protein-coupled receptor
SAR: structure–activity relationship
DPP-IV: dipeptidyl peptidase IV
NEP-24.11: neutral endopeptidase 24.11
T2DM: type-2 diabetes mellitus
*exo*BP: exogenous bioactive peptide
SPPS: solid-phase peptide synthesis
LLS: longest linear sequence
