# Supplementary material for: Selective Native N(in)–H Bond Activation in Peptides with Metallaphotocatalysis
Source: JACS Au. 2025 Mar 28;5(4):2040–6. doi: 10.1021/jacsau.5c00119 (PMC12042047; doi:10.1021/jacsau.5c00119)
Supplement: Supplementary file 1 — au5c00119_si_001.pdf [file au5c00119_si_001.pdf]

Supporting Information

Selective Native $N_{(in)}$-H Bond Activation in Peptides with Metallaphotocatalysis

José A. C. Delgado,[†*] Jéssica C. Amaral,[ϕ,¶] Paula S. Penteado,[¶] Antonio G. Ferreira,[¶] Maria Fátima G. F. da Silva,[¶] Burkhard König,[§*] and Márcio W. Paixão.[†*]

[†] Laboratory for Sustainable Organic Synthesis and Catalysis, Department of Chemistry, Federal University of São Carlos – UFSCar, Rodovia Washington Luís, km 235 - SP-310 - São Carlos - São Paulo - Brazil -13565-905.

[ϕ] Department of Plant Pathology and Nematology, University of São Paulo (USP)/ Luiz de Queiroz College of Agriculture (ESALQ), Av. Pádua Dias, 11 – Piracicaba – São Paulo – Brazil - 13418-900.

[¶] Department of Chemistry, Federal University of São Carlos – UFSCar, Rodovia Washington Luís, km 235 - SP-310 - São Carlos - São Paulo - Brazil -13565-905.

[§] Institute of Organic Chemistry, University of Regensburg, 93040 Regensburg, Germany.

To whom the correspondence should be addressed
**E-mail: joseacd1990@gmail.com*
burkhard.koenig@chemie.uni-regensburg.de
mwpaixao@ufscar.br

Supporting Information....1
1. General Information 2
2. General procedure (A) for solid-phase peptide synthesis....4
3. General procedure (B) for peptide cleavage, side chain deprotection, and work-up 6
4. General procedure (C) for Fmoc loading determination 6
5. General procedure (D) for on-resin metallaphotoredox catalyzed peptide arylation....8
6. Analytical RP-HPLC Data of peptide crudes....11
7. LC-MS Data of peptide crudes 14
8. Analytical RP-HPLC, LC-MS/MS and HRMS Data of pure peptides .38
9. NMR data....44
10. NMR Spectra....47
11. References 59
12. Reagents....59

13. Equipment and Materials 60

1. General Information

All solvents were dried and distilled before use by standard procedures and reagents were of the highest commercially available grade purchased from Oakwood Chemical, AmBeed, Nfinitu Tech LLC, ÊXODO científica, Biograde, Sigma-Aldrich, TCI Chemicals, or Merck Millipore and used as received or purified according to the procedures outlined in *Purification of Common Laboratory Chemicals*[1]. Laboratory Glasswares were dried in an oven or flame under a vacuum and cooled under an inert atmosphere. Unless otherwise noted, yields refer to chromatographically and spectroscopically pure compounds.

NMR spectroscopy: All NMR spectra were recorded at 298 K using a Bruker Avance III NMR spectrometer (Bruker-Biospin®, Ettlingen, Germany) operating at 11.4 T (600 MHz for ^{1}H and 150 MHz for ^{13}C) equipped with a 5 mm TCI cryoprobe. For all experiments, a ^{1}H 90° pulse length of 10 μs was used. Chemical shifts (δ) are reported in parts per million (ppm) relative to the residual solvent signals, and coupling constants (J) are reported in Hertz (Hz). The following abbreviations indicate the multiplicity of each signal: ^{1}H-NMR: br = broad, s = singlet, d = doublet, t = triplet, q = quartet, dd = doublet of doublets, dt = doublet of triplets, and m = multiplet. NMR spectra were processed using the Bruker TopSpin® 3.6.2 software package with apodization function lb=0.3.

For 1D-ROESY experiments, the parameters used were as follows: relaxation delay (2 s), number of scans (64), spectral width (20 ppm), number of FID points (32k), acquisition time (1.36 s), and mixing time (250 ms). Before processing, the data were zero-filled to 32k points and apodization function lb=1 Hz.

For 2D experiments—^{1}H-^{15}N HMBC—a standard pulse sequence was used with the following parameters: relaxation delay (1 s), acquisition time (0.17 s), spectral width F2 (20 ppm) and F1 (400 ppm), number of FID points (4k), number of experiments (64), number of spectra (64), and ^{1}H-^{15}N coupling constants for one-bond and long-range interactions (80 Hz and 8 Hz, respectively).

For ^{1}H-^{13}C HMBC, the parameters were as follows: relaxation delay (1 s), acquisition time (0.17 s), spectral width F2 (20 ppm) and F1 (230 ppm), number

of FID points (4k), number of experiments (256), number of spectra (96), and ^1H-^{13}C coupling constants for one-bond and long-range interactions (145 Hz and 8 Hz, respectively). For ^1H-^{13}C HSQC, the same parameters were used, except for the number of spectra, which was 16 and used the pulse sequence with INEPT block.

For TOCSY, a mlev standard pulse sequence was used with HDO presaturation. The parameters were as follows: relaxation delay (1 s), acquisition time (0.11 s), spectral width (15 ppm), number of FID points (2k), number of experiments (256), number of spectra (16), and mixing time (800 ms). For COSY, the parameters were: relaxation delay (1 s), acquisition time (0.17 s), spectral width (20 ppm), number of FID points (4k), number of experiments (256), and number of spectra (16).

Mass spectrometry: High-resolution mass spectrometry (HRMS) was performed using a UHPLC-QTOF/HRMS Agilent® - Infinity II 1290 UHPLC - 6545 QTOF system at the Laboratory of Natural Products, Federal University of São Carlos. Chromatographic separation was performed with an Agilent®, Zorbax Eclipse SB C-18 column (3,0 x 50 mm, 1,8 μm), using a gradient elution method. The mobile phases consisted of: H_2O + 0.1% formic acid and CH_3CN + 0.1% formic acid. The gradient program was as follows: 97:3 to 0:100 over the 20 min. The flow rate was set to 0.3 $mL.min^{-1}$, with an injection volume of 3 μL, and the column temperature was maintained at 40°C. Mass spectra were acquired in positive ionization mode, using MS *full scan* over the m/z range 50−1700. Instrumental settings were optimized as follows: capillary voltage at 3000 V, nozzle voltage 1000V, gas temperature 350ºC, drying gás 12 $L.min^{-1}$, nebulizer 35 psi, sheaf gas 300ºC, fragmentor 110V, skimmer 60V, and collision energy voltage 20 V. Additionally, MS/MS spectra were acquired in auto MS/MS mode for the 8 most intense ions over the range of *m/z* 50−3200, using the same conditions as for MS full scan. A linear collision energy ramp was applied based on the *m/z* range: 250 = 20eV, 1000 = 25eV, 1500 = 30eV, 2500 = 35eV and 3200 = 38eV. Data acquisition and data processing were performed using the MassHunter Data Acquisition and MassHunter Qualitative Navigator B.08.00 softwares, respectively.

Analytical RP-HPLC: All analytical RP-HPLC chromatograms were measured on a Shimadzu Prominence-*i* LC-2030C 3D Plus System (column:

Inertsil ODS-3V (250 x 4.6 mm, 5µm)) (λ= 226 nm) (Linear gradient of 5 to 95% of solvent B for 35 min, at 0.8 mL/min flow) (Solvent A: H_2O, 0.1 % TFA; solvent B: CH_3CN) to determine purity.

Purifications were performed on a Prominence UFLC (SPD-M20A (PDA detector), SIL-10AF (autosampler), DGU-20A5R (degasser), LC-20AT (pump), and CBM-20A (system controller)) system (column: Inertsil ODS-3V (250 x 4.6 mm, 5µm)) (λ= 226 nm) (Linear gradient of 5 to 95% of solvent B for 27 min, at 0.8 mL/min flow) (Solvent A: H_2O, 0.1% TFA; solvent B: CH_3CN, 0.1% TFA)—Autosampler configurations: injection volume: 50 µL; excess volume: 10 µL.

Photochemical setups and light sources (LEDs): The photochemical experiments were performed using a 446 nm LED (20 V, 1372 mW) array placed under a metal cooling block, in sealed reaction vials (This reactor setup is a custom-made device (University of Regensburg workshop) and is not a commercially available product). The distance between the reaction vial and the LED is 6.5 mm.

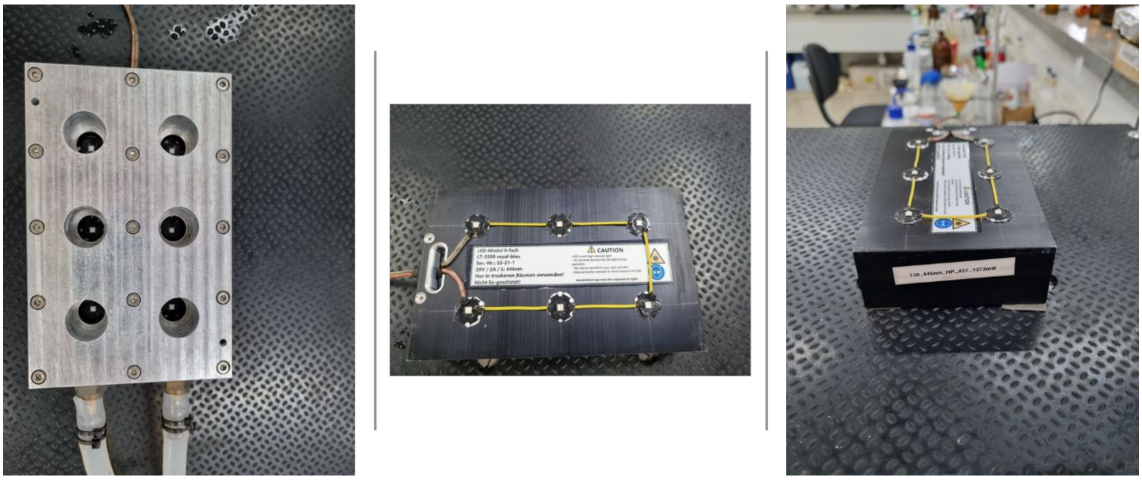

Figure 1. Photographs of the photochemical device (Cooling block and LED array plate).

2. General procedure (A) for solid-phase peptide synthesis

Coupling reactions were performed manually on the H-Rink amide ChemMatrix® resin (35-100 mesh) (400 mg, 0.45 mmol/g) by a stepwise Fmoc/tBu strategy.

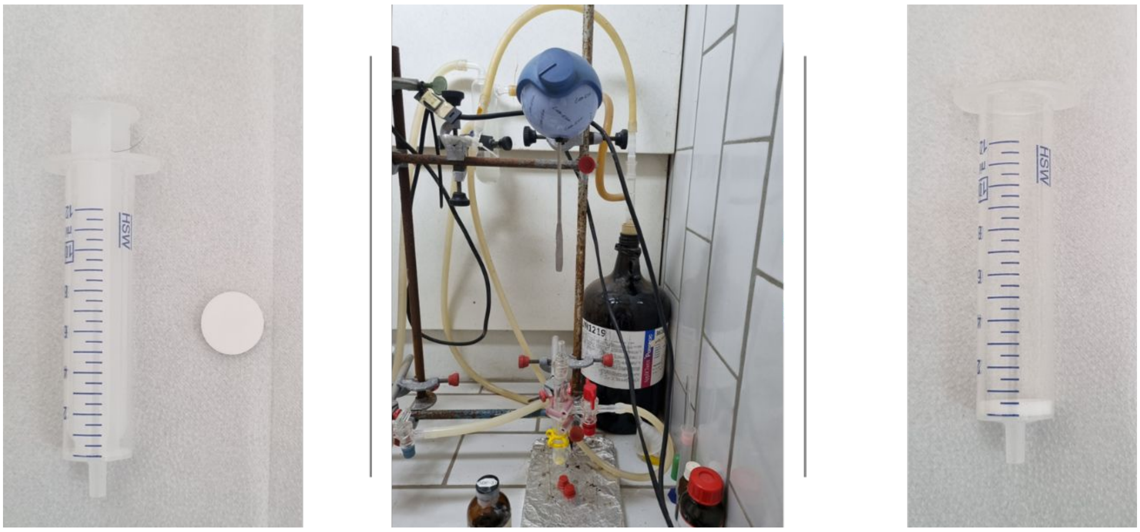

Figure 2. Photographs of the SPPS setup and SPPS reactor vessel.

The following protecting groups were used for the side chains: **Trt** for Asn, Gln, and His; **Boc** for Lys; ***t*-Bu** for Ser, Thr, Asp, Glu, and Tyr; and **Pbf** for Arg. Couplings were performed using 4 equivalents considering a resin's extended loading of 0.60 mmol/g according to the supplier. Each amino acid was coupled using N,N'-Diisopropylcarbodiimide (DIC)/1-Hydroxybenzotriazole hydrate (HOBt hydrate) activation and the completion of the coupling reaction was monitored by the Kaiser test.[3] Fmoc-deprotection was carried out using a 20% piperidine solution in DMF. Capping was performed using a 20% acetic anhydride solution in DMF containing 2% Diisopropylethylamine (DIPEA). After the analog peptide sequences were elongated, the resin was washed with DMF, MeOH, and Et_2O and dried to afford 1.140 g of resin with **GLP-1 (7-37)** attached and 792 mg of resin with **Flufirvitide-3** attached respectively. Unless otherwise noted, each sample mass was partitioned into four parts and subjected to general procedure D.

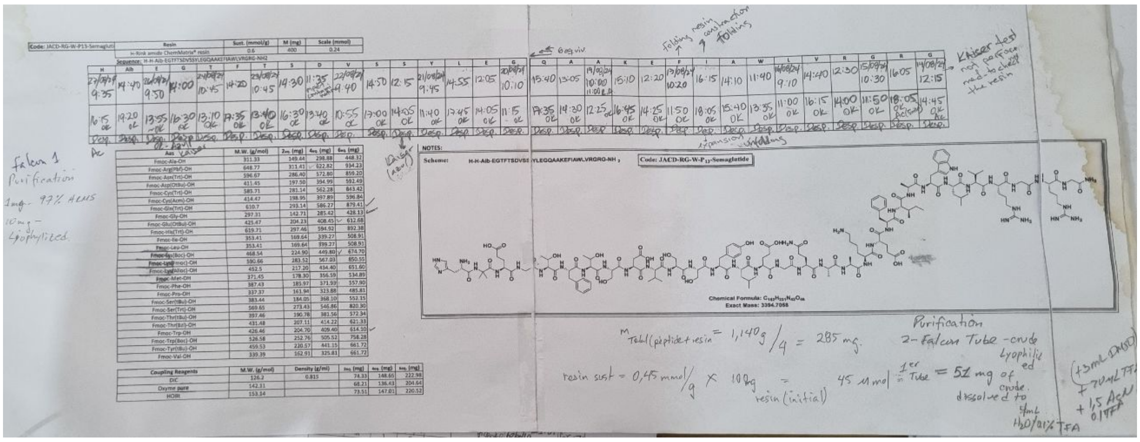

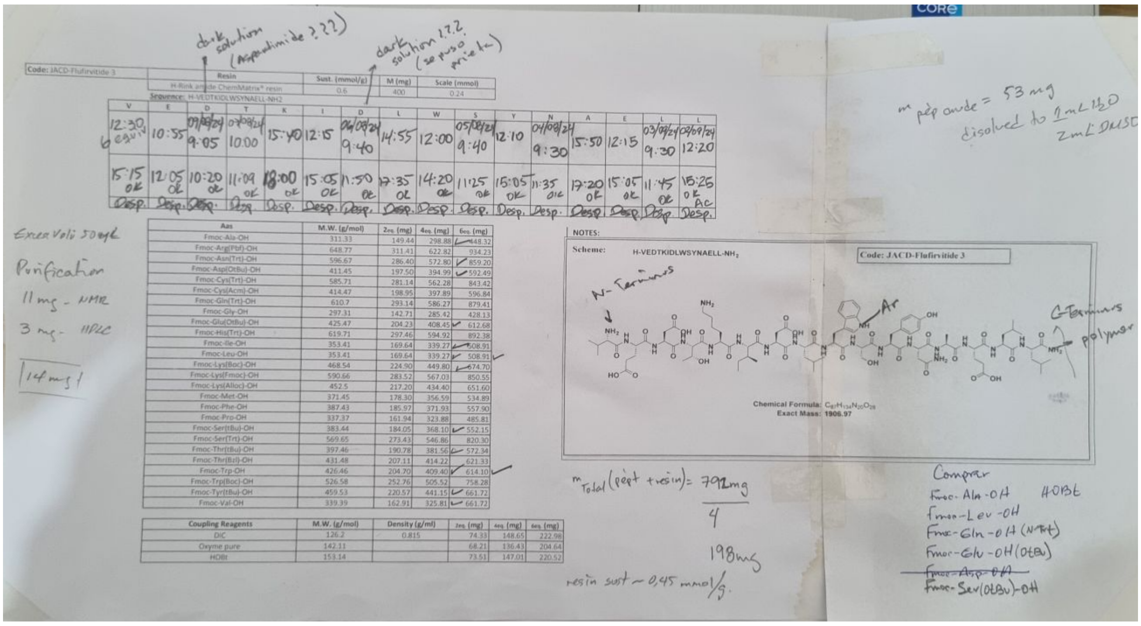

Figure 3. Photographs of the experimental SPPS sheets.

3. General procedure (B) for peptide cleavage, side chain deprotection, and work-up

Peptides were released entirely unprotected from the resin by treatment with a cocktail of trifluoroacetic acid (TFA)/Triisopropylsilane (TIPS)/water (95:2.5:2.5) (*ca.,* V = 22 mL/g) for 2 hours at room temperature. When the time has elapsed, precipitation in diethyl ether at -18 ^{0}C was carried out and the precipitates were centrifuged. Two cycles of precipitate washing with diethyl ether followed by centrifugation were carried out. Finally, the precipitated peptides were dissolved in water/acetonitrile (40%) and lyophilized. Arylated crude peptide analogs were analyzed by RP-HPLC to determine purity and LC-MS characterization. Unless otherwise noted, the products were purified by semi-preparative RP-HPLC, and the pure products were characterized by RP-HPLC, NMR, and ESI-HRMS analysis.

4. General procedure (C) for Fmoc loading determination

To 100 mg of pre-swelling H-Rink amide ChemMatrix® resin (35-100 mesh) was attached Fmoc-*L*-Gly-OH following the general procedure A for 24 hours. Then the resin was washed with DMF, MeOH, and Et_2O and dried for 24 hours to give a negative Kaiser test. In triplicate, 10 mg of resin with peptide attached was placed in a polypropylene 2.0 mL Eppendorf tube, then 200 μL of a 20% piperidine solution in DMF was added and shaken for 10 min. The solution

was then transferred with a Pasteur pipette with cotton wrapped at the tip to a 3 mL polypropylene syringe equipped with a syringe filter PTFE, 0.45 μm, 25 mm and filtered into a 25 mL volumetric flask (another 200 μL was added to repeat this step). Finally, the solutions were made up to 25 mL with EtOH.

Standard Solution Preparation: About 4 mg of the corresponding/same Fmoc-amino acid being analyzed was transferred to a 25 mL volumetric flask; 400 μL of the deprotection solution (20% piperidine-DMF) was added, and the volume was made up to 25 mL with EtOH.

Blank Solution Preparation: 400 μL of the deprotection solution (20% piperidine-DMF) was transferred to a 25 mL volumetric flask, and the volume was made up to 25 mL with EtOH.

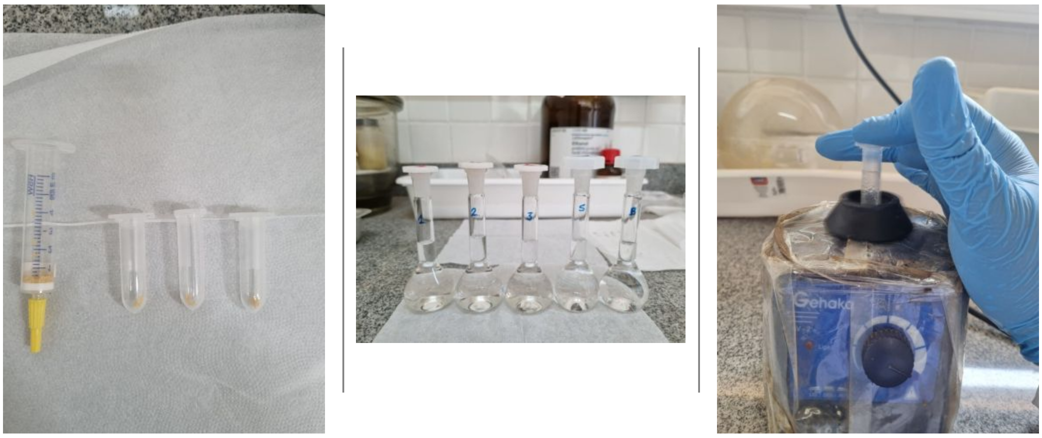

Figure 4. Photographs of the Fmoc loading determination procedure (resin and solution samples).

The UV absorbance of the samples and the standard was recorded versus the blank solution and the loading was calculated as follows considering the absorbance value at 301 nm:[4]

$$Resin\ loading = \frac{Sample\ (UV\ Abs)\ x\ Stand\ mass(g) x\ 1000}{Stand\ (UV\ Abs) x\ loading\ resin\ mass\ (g) x\ MW\ (Fmoc\ Aas)}$$

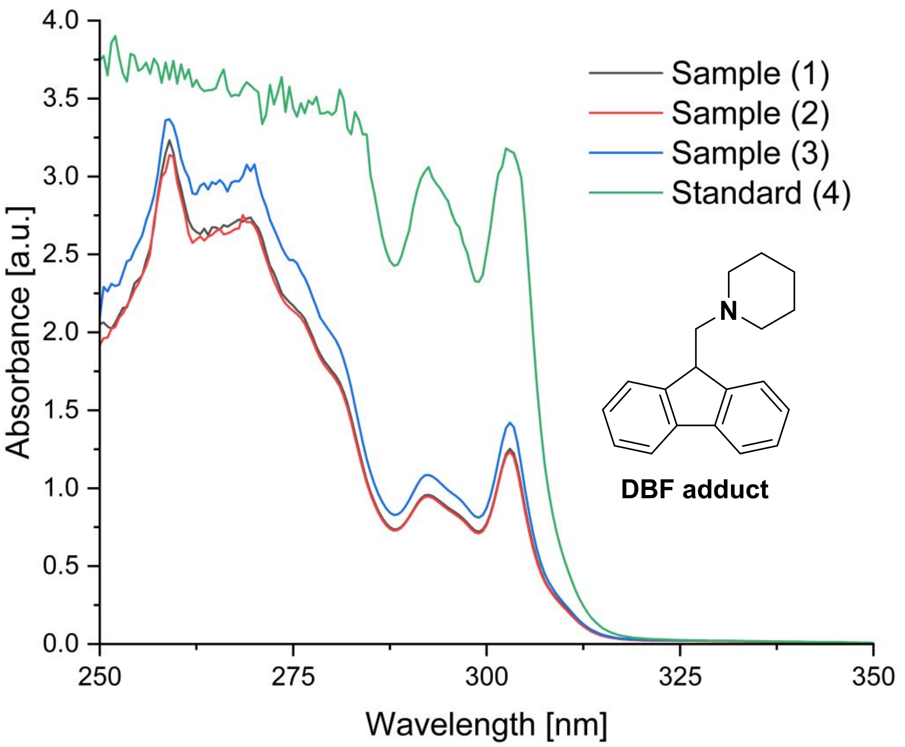

Figure 5. UV-Visible spectra of the samples and the internal standard. Resin loading of 0.45 mmol/g.

5. General procedure (D) for on-resin metallaphotoredox catalyzed peptide arylation.

A 5 mL crimp reaction vial was charged with dry resin with peptide attached (45 μmol, *ca.*, 285 mg and 198 mg for the **GLP-1 (7-37)** and **Flufirvitide-3** analogs respectively), 4-Bromobenzonitrile (74 mg, 9 equiv.), and K_2HPO_4 (141 mg, 18 equiv.). Noted that for the acetylated **GLP-1 (7-37)** analog sequence *L*-Alanine methyl ester hydrochloride (6 mg, 1 equiv.) was used as additive. Separately, 4-CzIPN (1 mL stock solution 17.8 mg/mL in DMF), $NiCl_2$-Glyme (1 mL stock solution 19.8 mg/mL in DMF), and 4,4'-Di-tert-butyl-2,2'-dipyridyl (1 mL stock solution 24.2 mg/mL in DMF) were prepared. Then, DMF (3.7 mL) and 100 μL of each stock solution were added, and the crimp vial was capped and stored at -18 ^{0}C overnight to ensure good resin swelling.

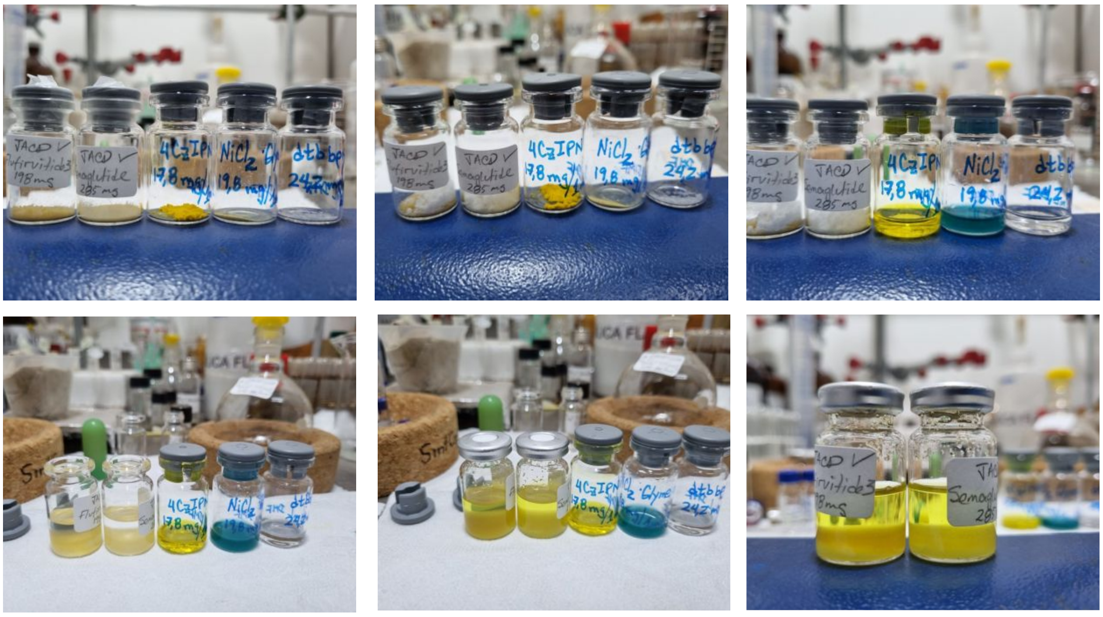

Figure 6. Photographs of the metallaphotoredox procedure (Step by step visualization for the preparation of photoreactor vials and stock solutions).

Thereafter, the crimp vial was kept at room temperature and bubbled with N_2 for 15 min. Then, the vial was placed in a thermostatic cooling block (25 ^{0}C), shaken, and irradiated through the panel bottom side of the vial by a 446 nm LED (20 V, 1372 mW) for 3 hours.

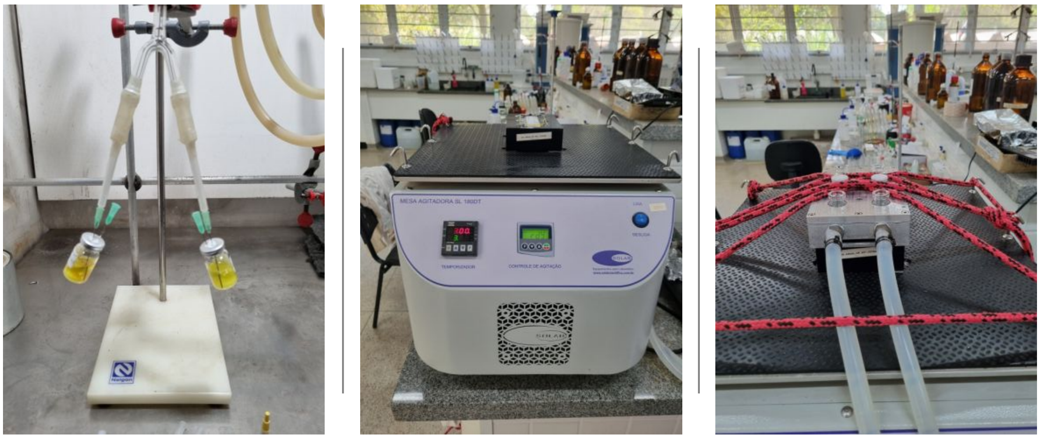

Figure 7. Photographs of the metallaphotoredox procedure (visualization of the degassing procedure and the configurations for solid-phase photochemical reactions).

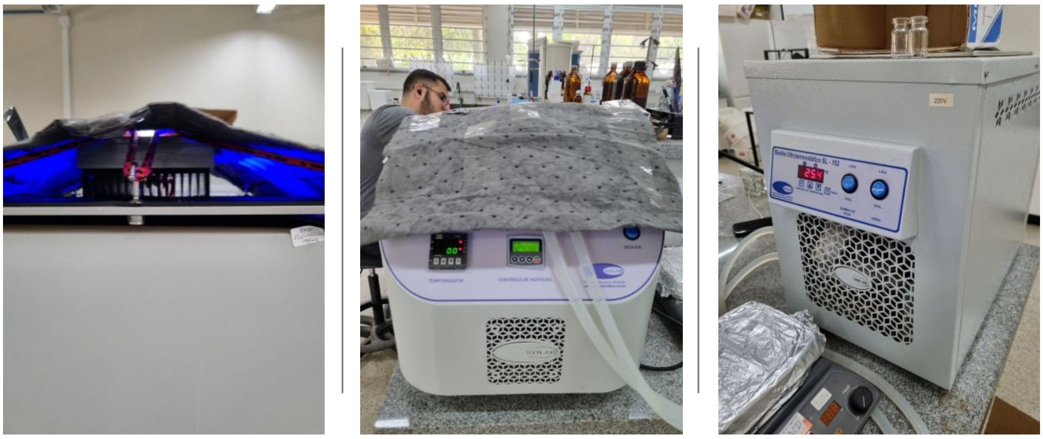

Figure 8. Photographs of the metallaphotoredox procedure (visualization of the configurations for solid-phase photochemical reactions).

Once time passed, the resin beads were transferred to a polypropylene reaction vessel with a frit at the bottom, washed with DMF, MeOH, and Et_2O, and dried. Finally, all crude peptides were obtained by general procedure B (104 mg and 53 mg of lyophilized ***N*$_{(in)}$-arylated GLP-1 (7-37)** and **Flufirvitide-3** crudes respectively). Crude products were dissolved in DMSO and purified by semi-preparative RP-HPLC. Unless otherwise noted, the pure products were characterized by RP-HPLC, NMR, and ESI-HRMS analysis.

6. Analytical RP-HPLC Data of peptide crudes

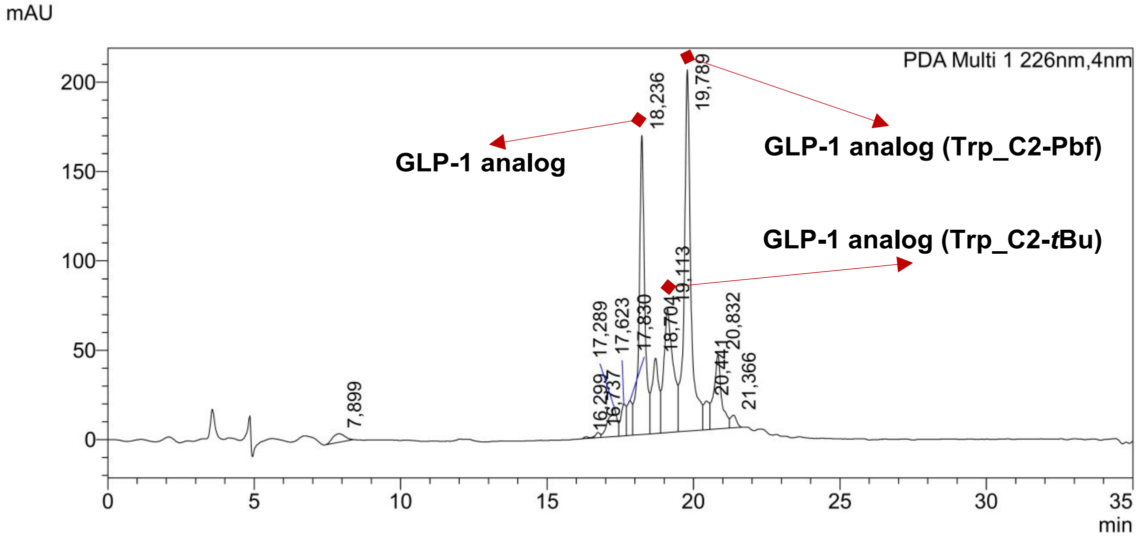

Figure 9. RP-HPLC chromatogram of the **GLP-1 (7-37)** analog. (72% purity of the crude peptide combining peaks with retention time 18.236, 19.113, and 19.789 min).

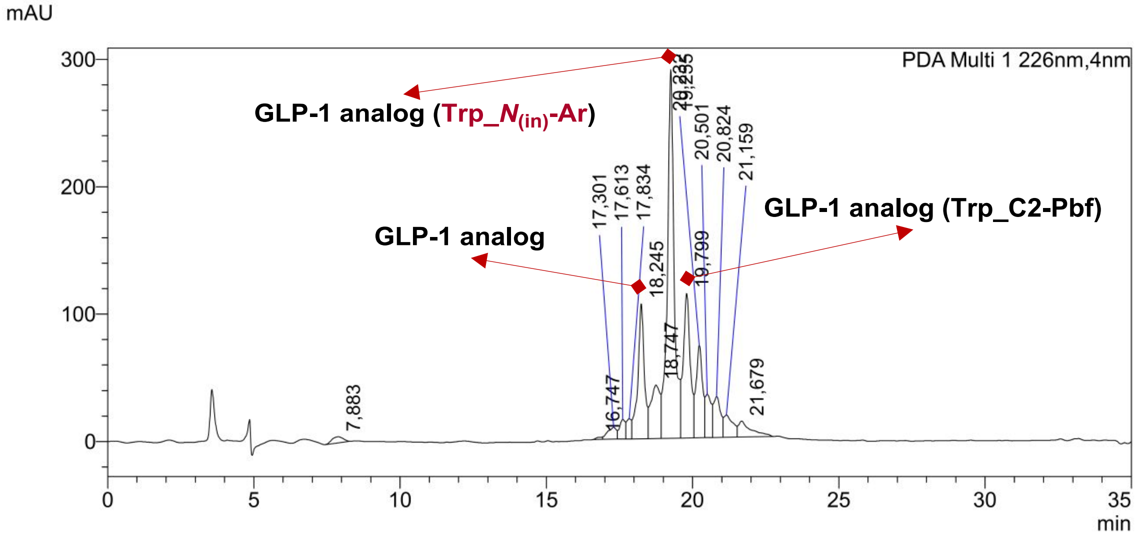

Figure 10. RP-HPLC chromatogram of the ***N*(in)-arylated GLP-1 (7-37)** analog. (57% conversion, 37% purity).

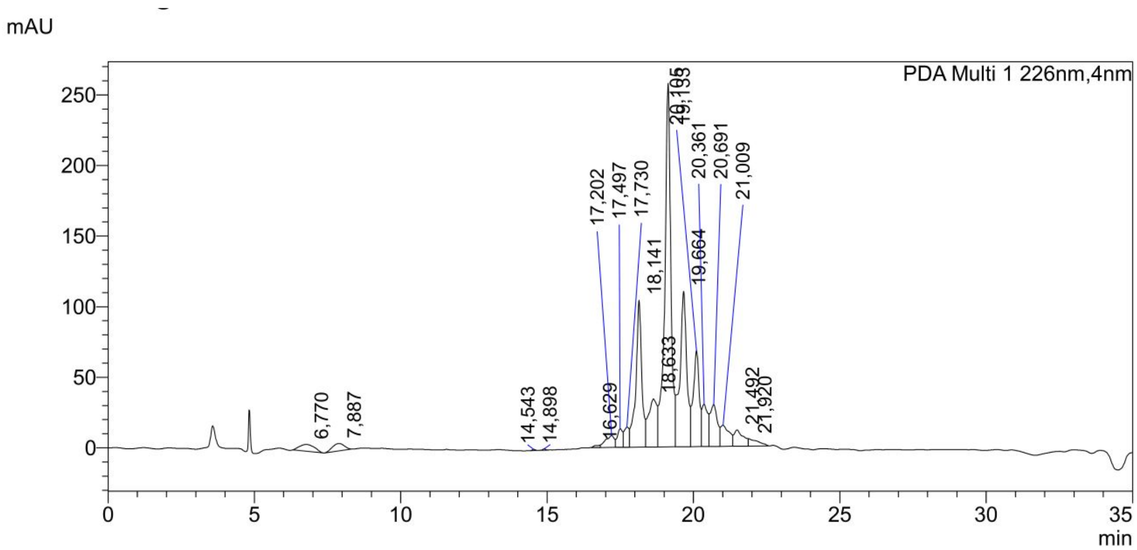

Figure 11. RP-HPLC chromatogram of the ***N*$_{(in)}$-arylated GLP-1 (7-37)** analog. Reaction performed using 3 equivalents of 4-Bromobenzonitrile (53% conversion, 33% purity).

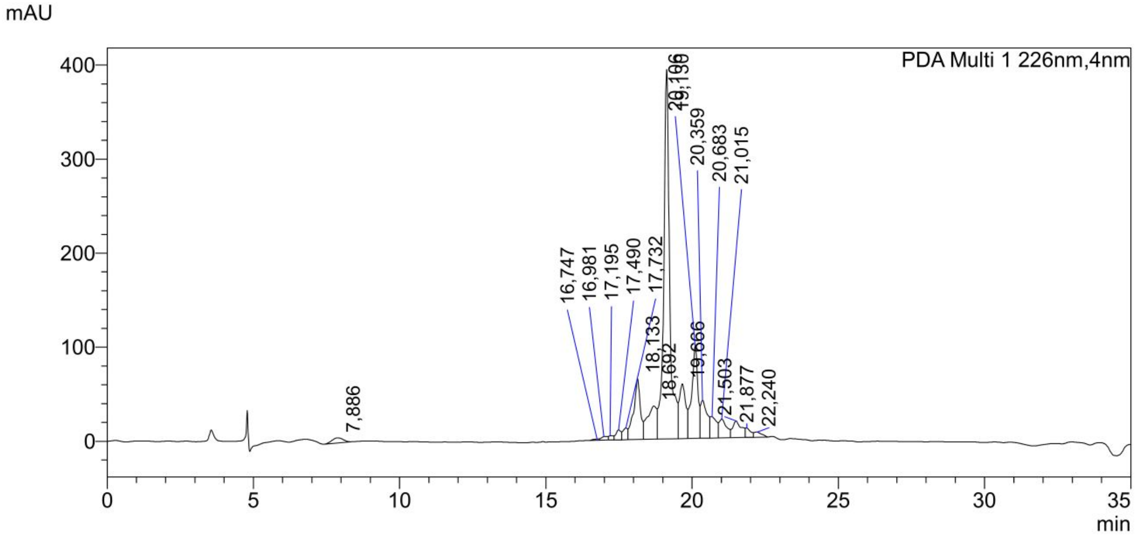

Figure 12. RP-HPLC chromatogram of the ***N*$_{(in)}$-arylated GLP-1 (7-37)** analog. Reaction performed using 3 equivalents of 4-Bromobenzonitrile over 2 rounds (74% conversion, 45% purity).

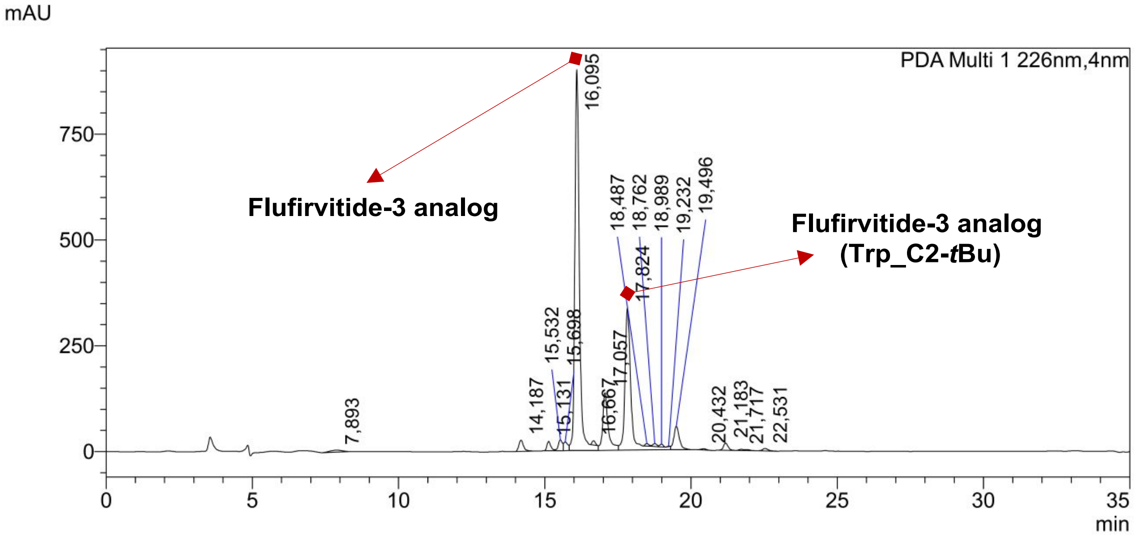

Figure 13. RP-HPLC chromatogram of the **Flufirvitide-3** analog. (78% purity of the crude peptide combining peaks with retention time 16.095 and 17.824 min).

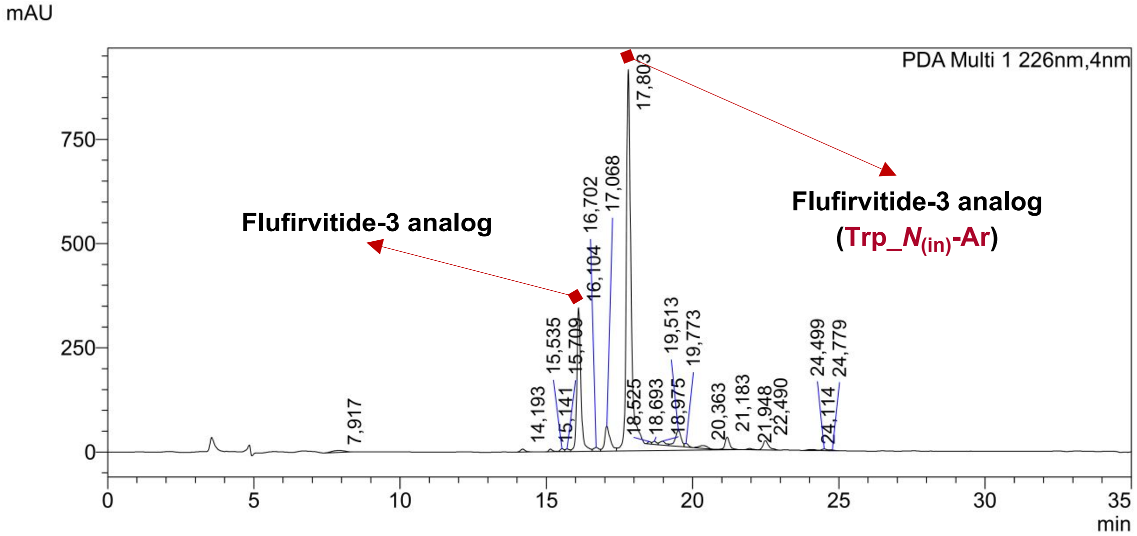

Figure 14. RP-HPLC chromatogram of the ***N*$_{(in)}$-arylated Flufirvitide-3** analog. (78% conversion, 65% purity). Note that the *tert*-butylated impurity coming from unreacted Flufirvitide-3 analog was detected by LC-MS analysis and it overlaps with the product in the shown chromatogram.

7. LC-MS Data of peptide crudes

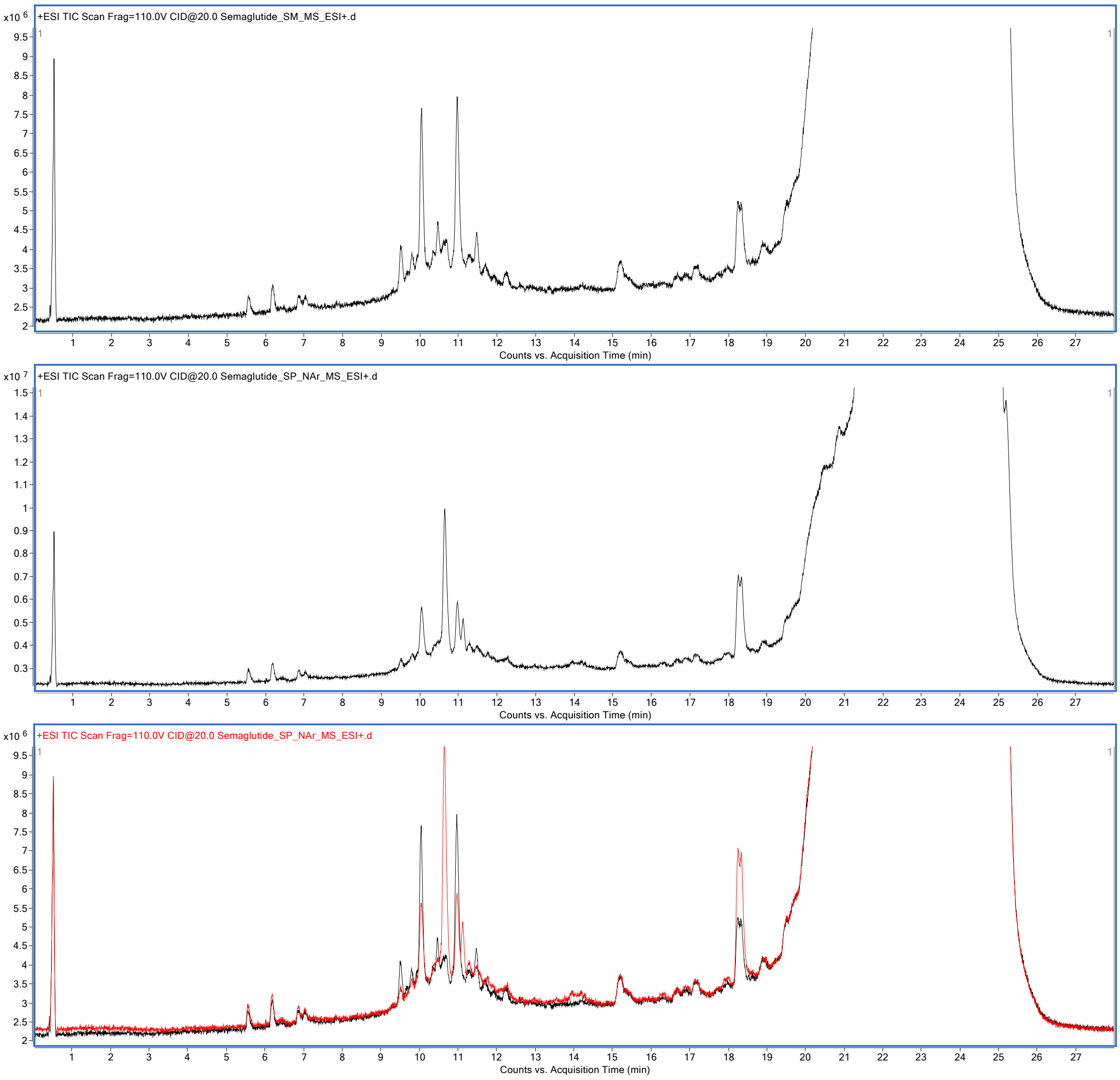

Figure 15. From top to bottom, LC-MS chromatograms (TIC) of the **GLP-1 (7-37)** analog, ***N*$_{\mathbf{(in)}}$-arylated GLP-1 (7-37)** analog, and overlapped TIC chromatograms.

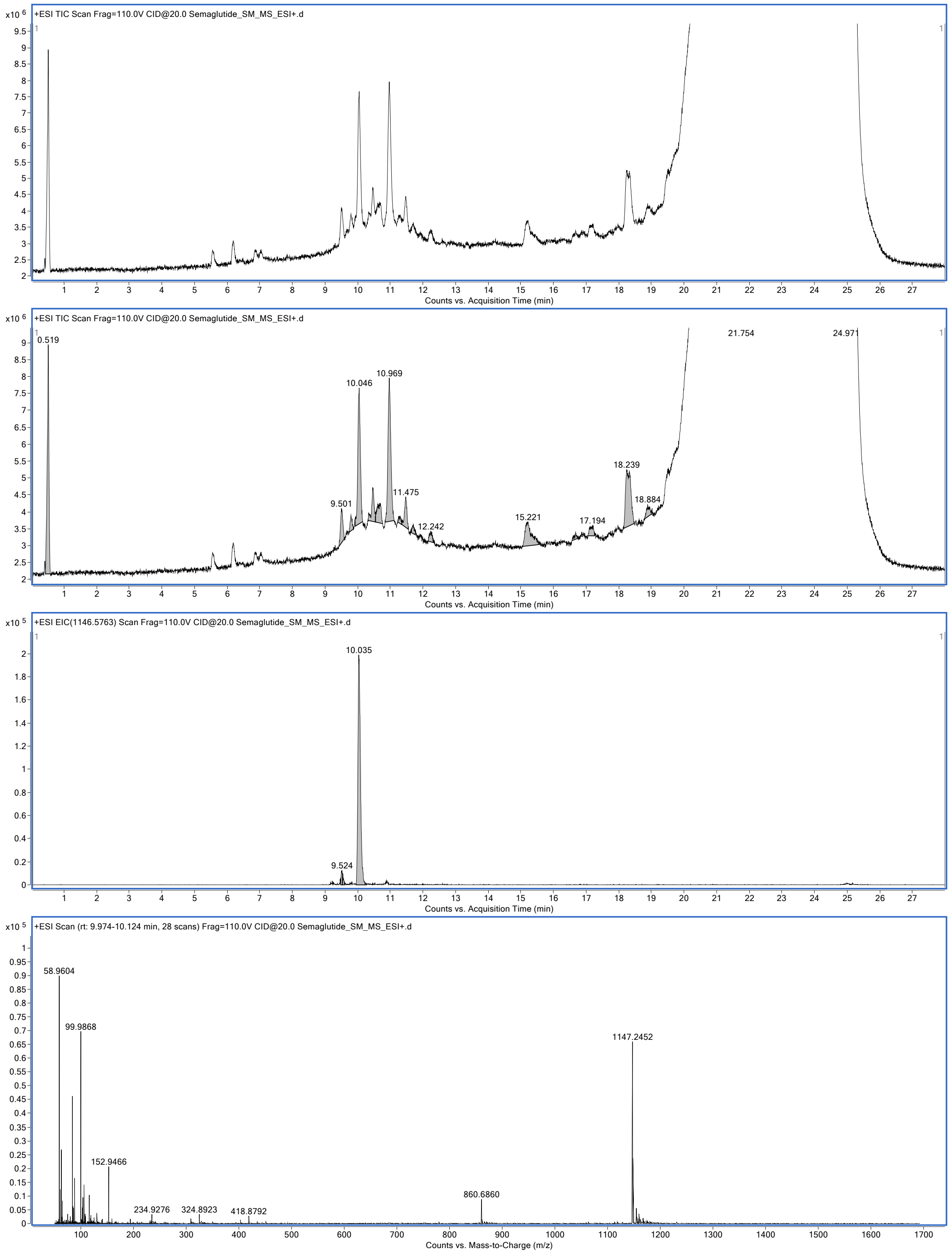
+ESI TIC Scan Frag=110.0V CID@20.0 Semaglutide_SM_MS_ESI+.d
Counts vs. Acquisition Time (min)
+ESI TIC Scan Frag=110.0V CID@20.0 Semaglutide_SM_MS_ESI+.d
0.519
10.046
10.969
9.501
11.475
12.242
15.221
17.194
18.239
18.884
21.754
24.971
Counts vs. Acquisition Time (min)
+ESI EIC(1146.5763) Scan Frag=110.0V CID@20.0 Semaglutide_SM_MS_ESI+.d
10.035
9.524
Counts vs. Acquisition Time (min)
+ESI Scan (rt: 9.974-10.124 min, 28 scans) Frag=110.0V CID@20.0 Semaglutide_SM_MS_ESI+.d
58.9604
99.9868
152.9466
234.9276
324.8923
418.8792
860.6860
1147.2452
Counts vs. Mass-to-Charge (m/z)

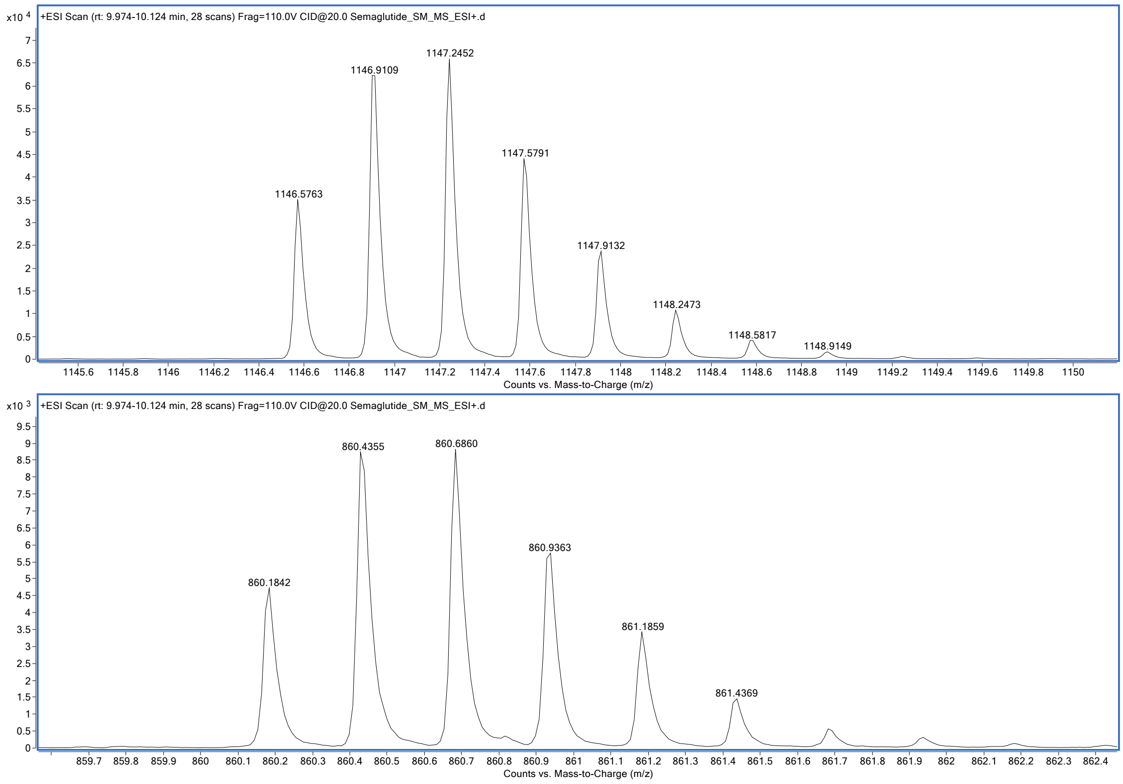

Figure 16. From top to bottom, LC-MS chromatograms (TIC, EIC) and HRMS spectra (m/z: $[M+3H]^{3+}$ Calcd. for $C_{154}H_{233}N_{43}O_{47}$ 1146.5794; Found: 1146.5763, error: -2.71 ppm, and $[M+4H]^{4+}$ Calcd. for $C_{154}H_{233}N_{43}O_{47}$ 860.1864; Found: 860.1842, error: -2.53 ppm) of the **GLP-1 (7-37)** analog.

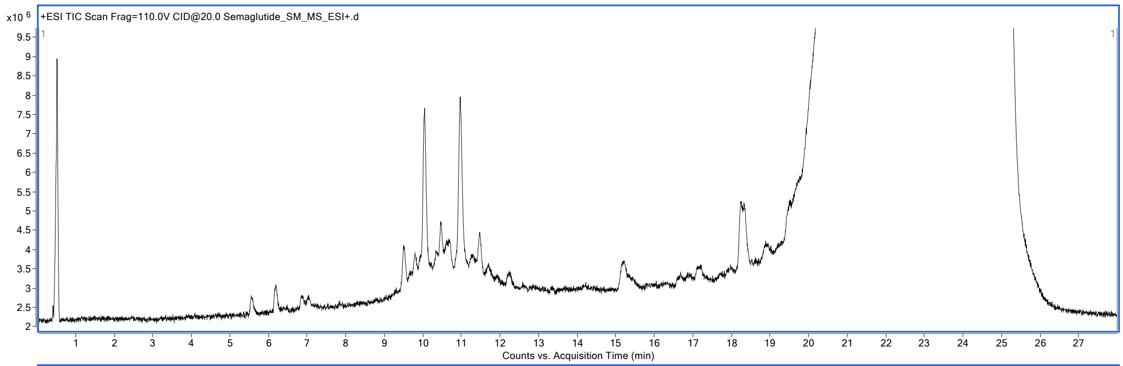
+ESI TIC Scan Frag=110.0V CID@20.0 Semaglutide_SM_MS_ESI+.d
Counts vs. Acquisition Time (min)

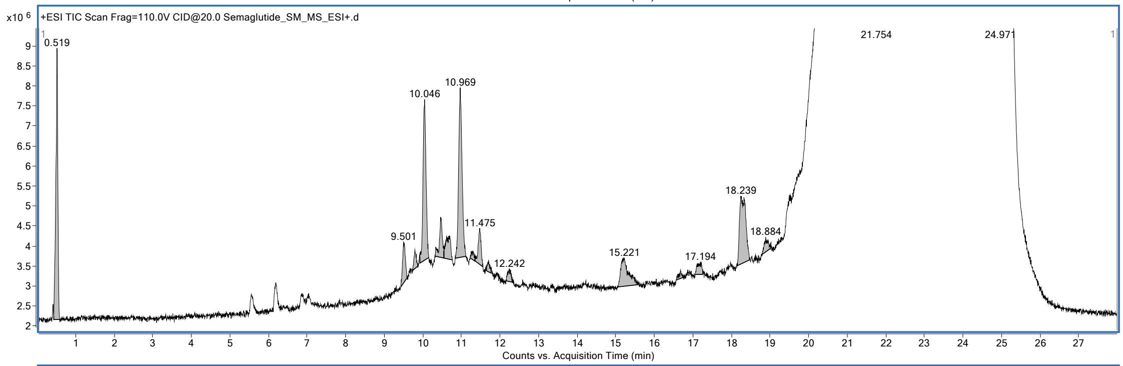
+ESI TIC Scan Frag=110.0V CID@20.0 Semaglutide_SM_MS_ESI+.d
0.519
10.046
10.969
9.501
11.475
12.242
15.221
17.194
18.239
18.884
21.754
24.971
Counts vs. Acquisition Time (min)

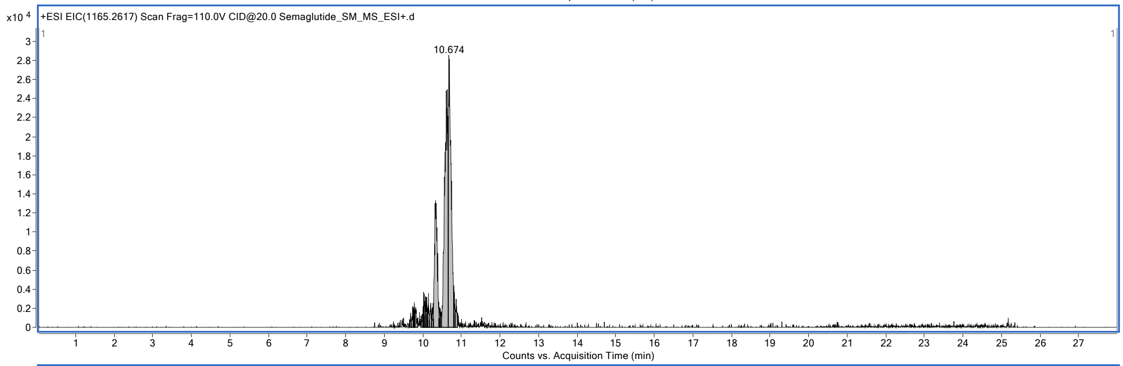
+ESI EIC(1165.2617) Scan Frag=110.0V CID@20.0 Semaglutide_SM_MS_ESI+.d
10.674
Counts vs. Acquisition Time (min)

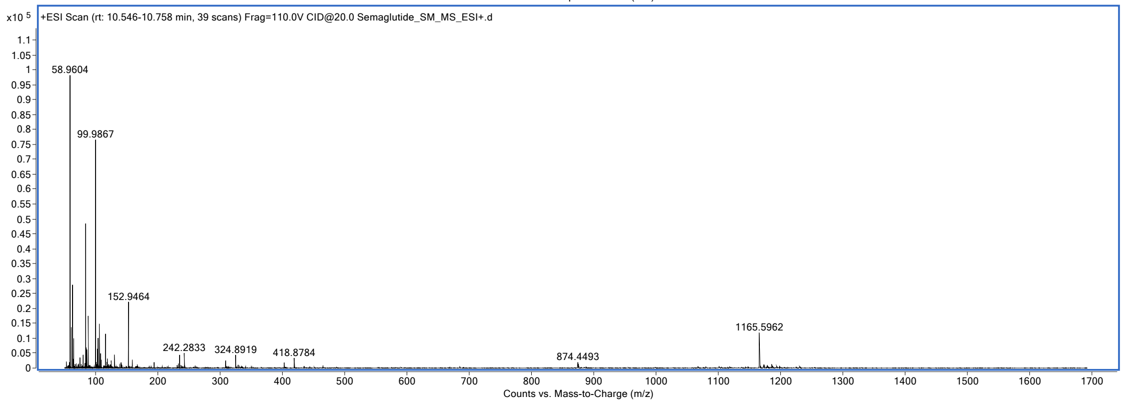
+ESI Scan (rt: 10.546-10.758 min, 39 scans) Frag=110.0V CID@20.0 Semaglutide_SM_MS_ESI+.d
58.9604
99.9867
152.9464
242.2833
324.8919
418.8784
874.4493
1165.5962
Counts vs. Mass-to-Charge (m/z)

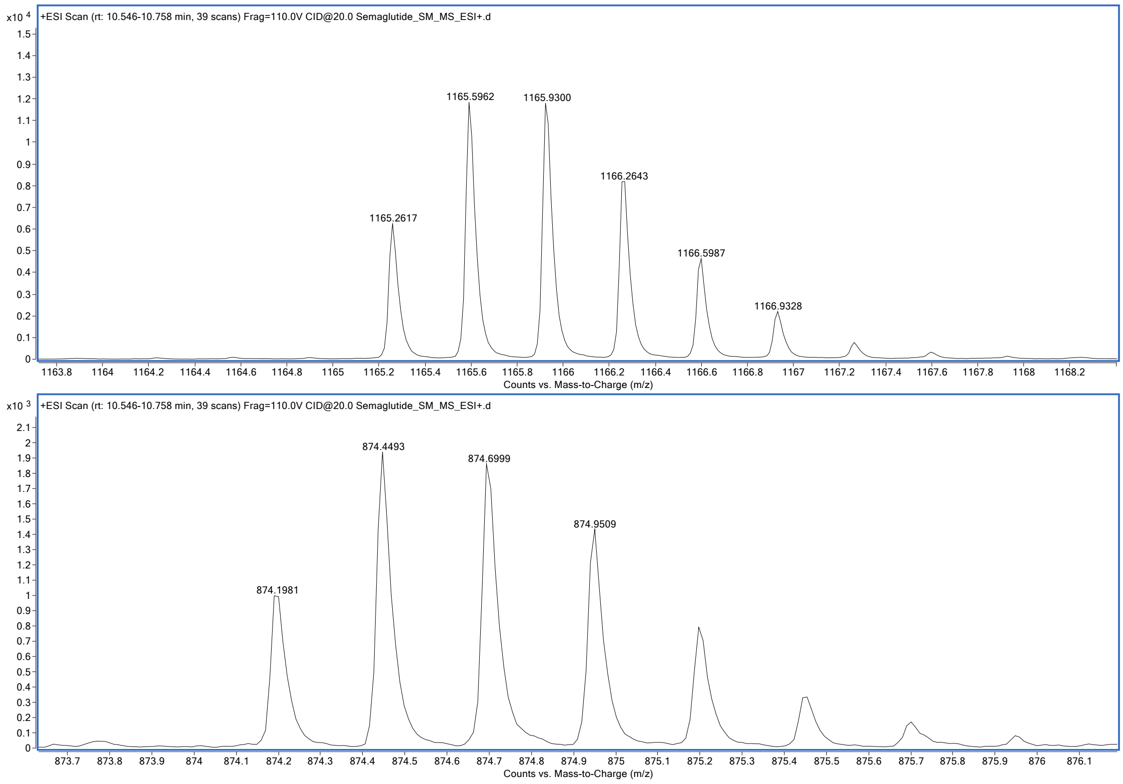

Figure 17. From top to bottom, LC-MS chromatograms (TIC, EIC) and HRMS spectra (m/z: $[M+3H]^{3+}$ Calcd. for $C_{158}H_{241}N_{43}O_{47}$ 1165.2669; Found: 1165.2617, error: -4.50 ppm, and $[M+4H]^{4+}$ Calcd. for $C_{158}H_{241}N_{43}O_{47}$ 874.2020; Found: 874.1981, error: -4.49 ppm) of the **(Trp_C2-*t*Bu) GLP-1 (7-37)** analog.

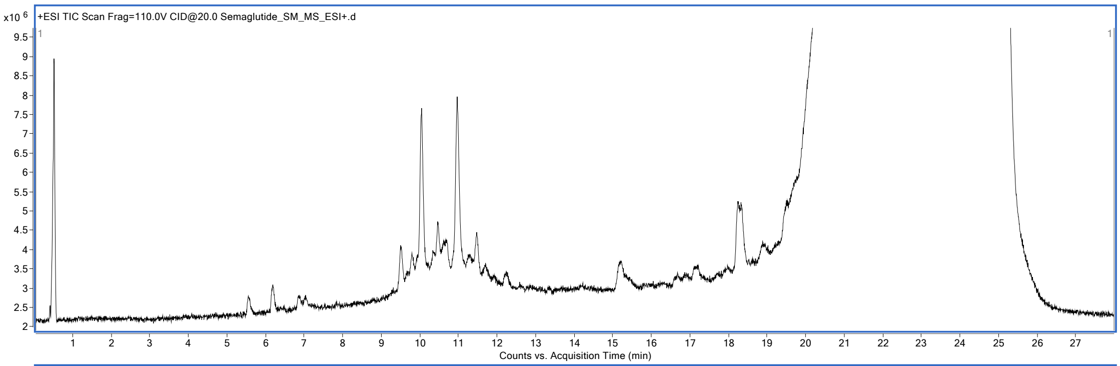
x10 6
+ESI TIC Scan Frag=110.0V CID@20.0 Semaglutide_SM_MS_ESI+.d
Counts vs. Acquisition Time (min)

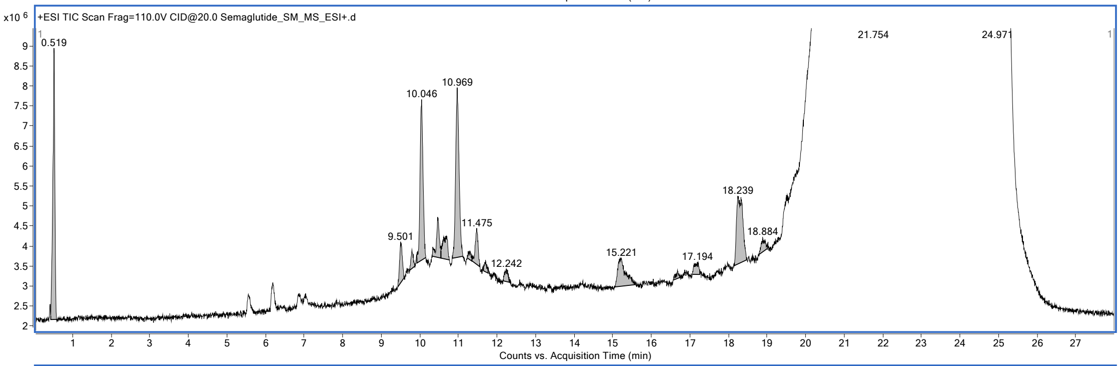
x10 6
+ESI TIC Scan Frag=110.0V CID@20.0 Semaglutide_SM_MS_ESI+.d
0.519
10.046
10.969
9.501
11.475
12.242
15.221
17.194
18.239
18.884
21.754
24.971
Counts vs. Acquisition Time (min)

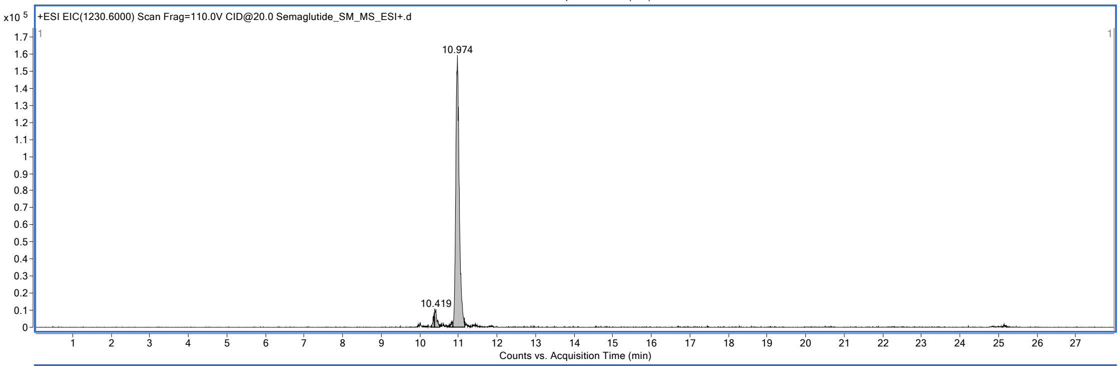
x10 5
+ESI EIC(1230.6000) Scan Frag=110.0V CID@20.0 Semaglutide_SM_MS_ESI+.d
10.974
10.419
Counts vs. Acquisition Time (min)

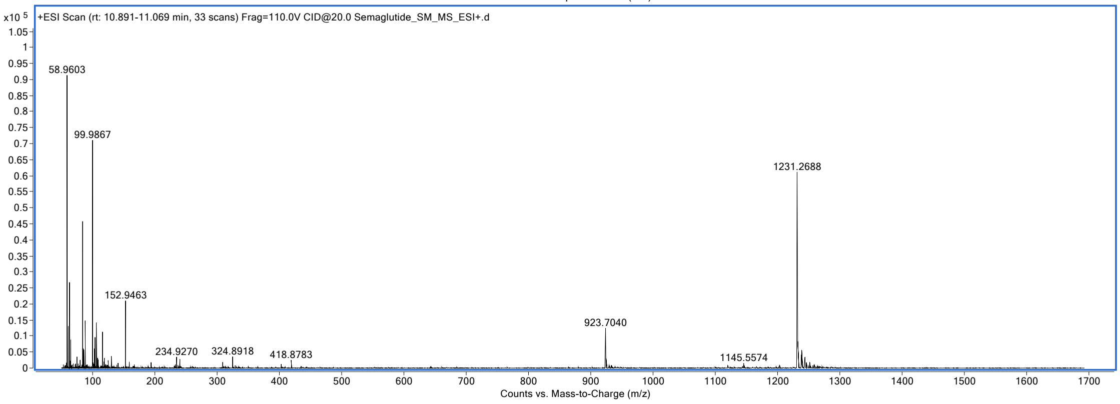
x10 5
+ESI Scan (rt: 10.891-11.069 min, 33 scans) Frag=110.0V CID@20.0 Semaglutide_SM_MS_ESI+.d
58.9603
99.9867
152.9463
234.9270
324.8918
418.8783
923.7040
1145.5574
1231.2688
Counts vs. Mass-to-Charge (m/z)

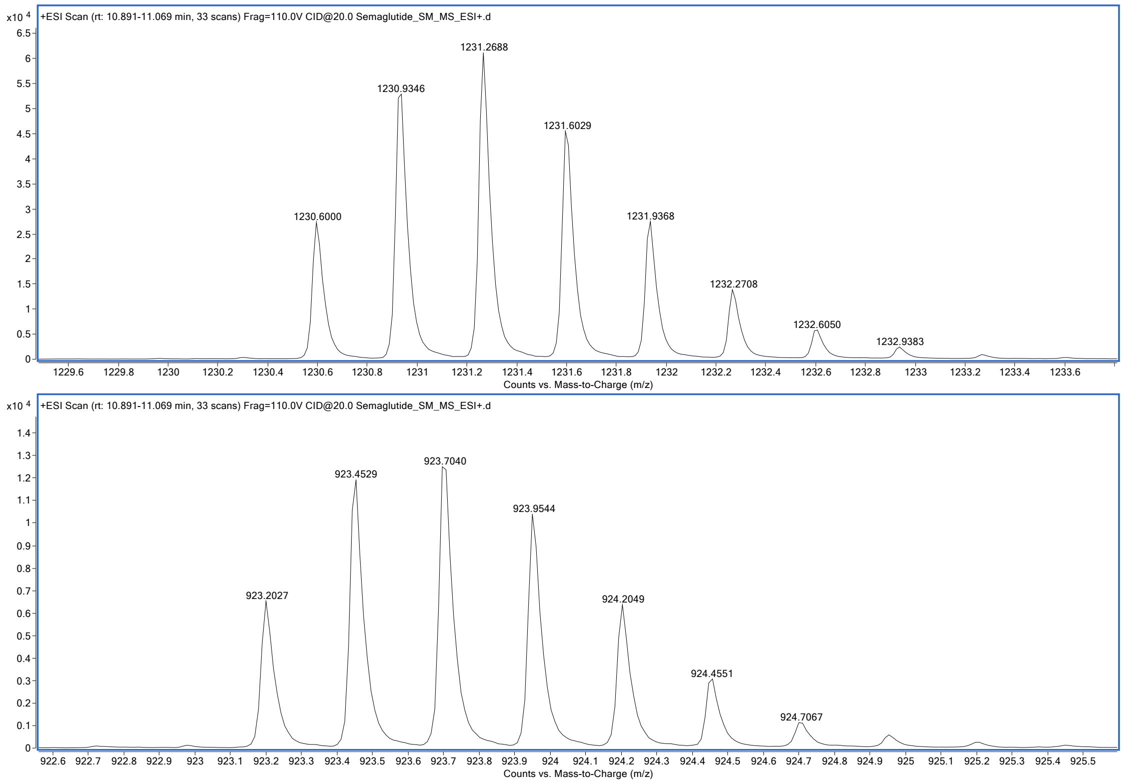

Figure 18. From top to bottom, LC-MS chromatograms (TIC, EIC) and HRMS spectra (m/z: $[M+3H]^{3+}$ Calcd. for $C_{167}H_{249}N_{43}O_{50}S$ 1230.6067; Found: 1230.6000, error: -5.48 ppm, and $[M+4H]^{4+}$ Calcd. for $C_{167}H_{249}N_{43}O_{50}S$ 923.2069; Found: 923.2027, error: -4.53 ppm) of the **(Trp_C2-Pbf) GLP-1 (7-37)** analog.

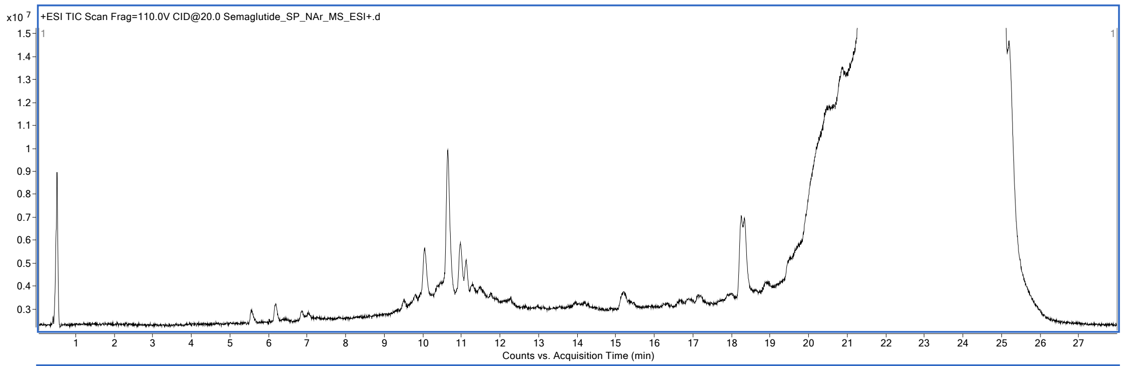
+ESI TIC Scan Frag=110.0V CID@20.0 Semaglutide_SP_NAr_MS_ESI+.d
x10 7
Counts vs. Acquisition Time (min)

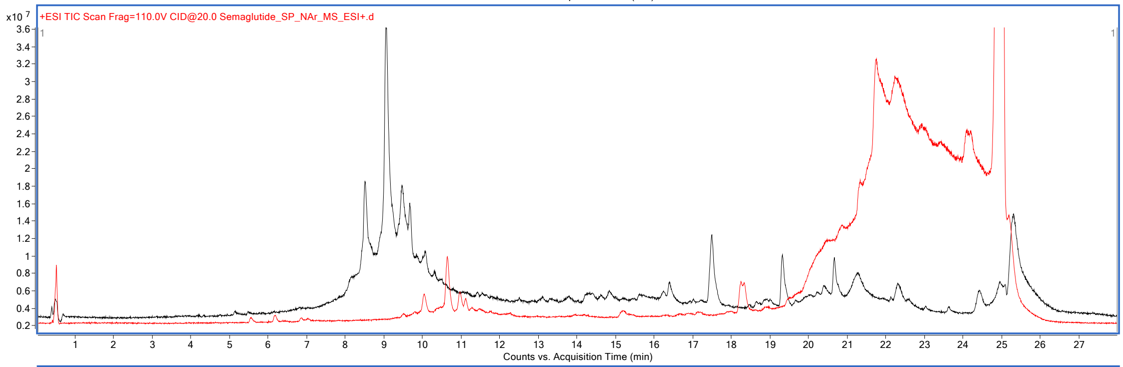
+ESI TIC Scan Frag=110.0V CID@20.0 Semaglutide_SP_NAr_MS_ESI+.d
x10 7
Counts vs. Acquisition Time (min)

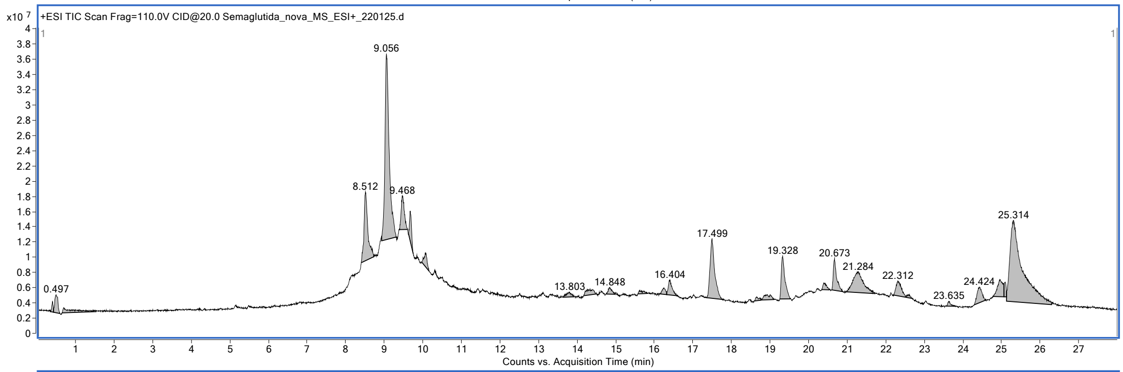
+ESI TIC Scan Frag=110.0V CID@20.0 Semaglutida_nova_MS_ESI+_220125.d
x10 7
9.056
8.512
9.468
0.497
13.803
14.848
16.404
17.499
19.328
20.673
21.284
22.312
23.635
24.424
25.314
Counts vs. Acquisition Time (min)

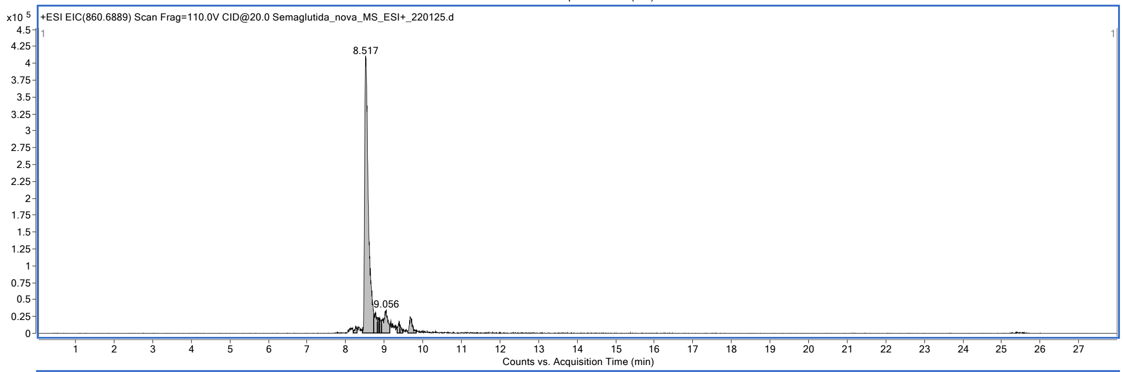
+ESI EIC(860.6889) Scan Frag=110.0V CID@20.0 Semaglutida_nova_MS_ESI+_220125.d
x10 5
8.517
9.056
Counts vs. Acquisition Time (min)

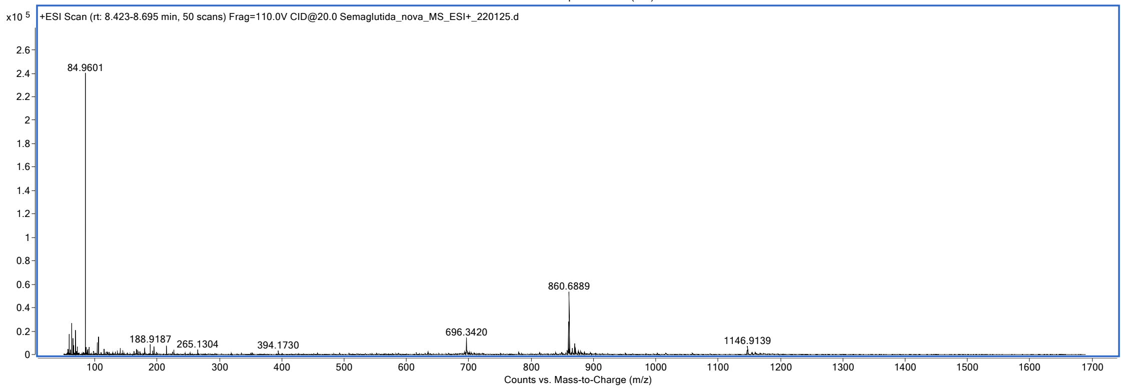
+ESI Scan (rt: 8.423-8.695 min, 50 scans) Frag=110.0V CID@20.0 Semaglutida_nova_MS_ESI+_220125.d
x10 5
84.9601
188.9187
265.1304
394.1730
696.3420
860.6889
1146.9139
Counts vs. Mass-to-Charge (m/z)

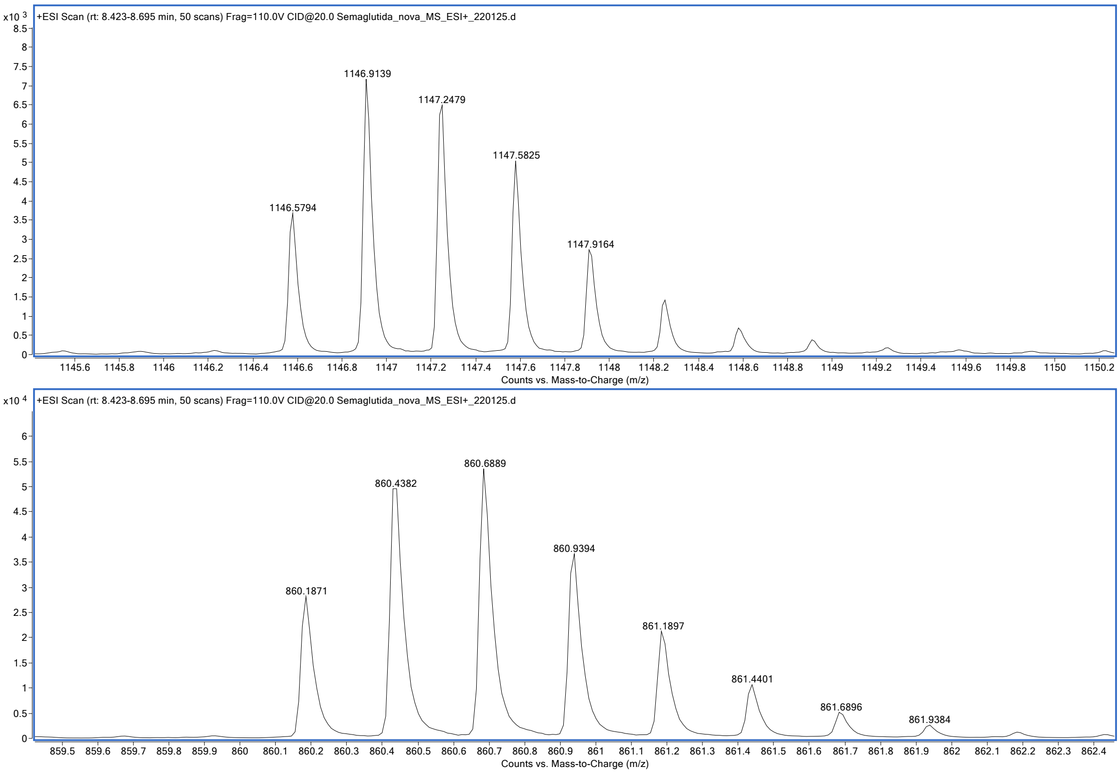

Figure 19. From top to bottom, LC-MS chromatograms (TIC, EIC) and HRMS spectra (m/z: $[M+3H]^{3+}$ Calcd. for $C_{154}H_{233}N_{43}O_{47}$ 1146.5794; Found: 1146.5794, error: -0.01 ppm, and $[M+4H]^{4+}$ Calcd. for $C_{154}H_{233}N_{43}O_{47}$ 860.1864; Found: 860.1871, error: 0.84 ppm) of the **GLP-1 (7-37)** analog.

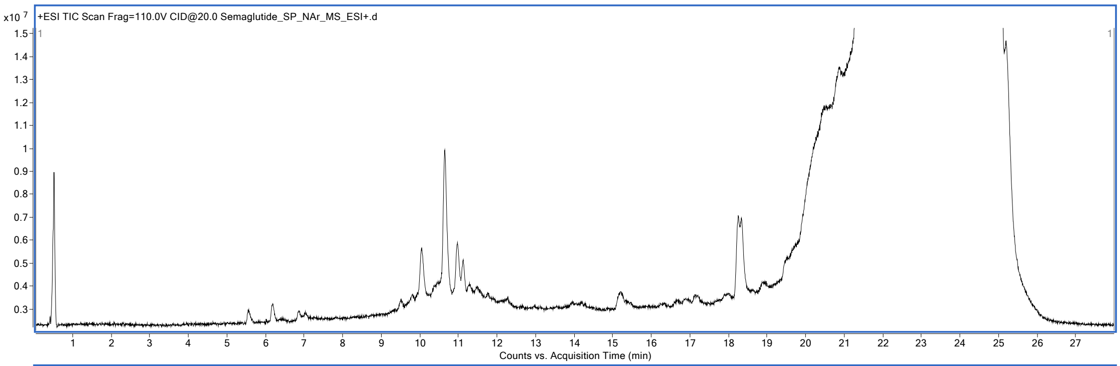
x10 7
+ESI TIC Scan Frag=110.0V CID@20.0 Semaglutide_SP_NAr_MS_ESI+.d
Counts vs. Acquisition Time (min)

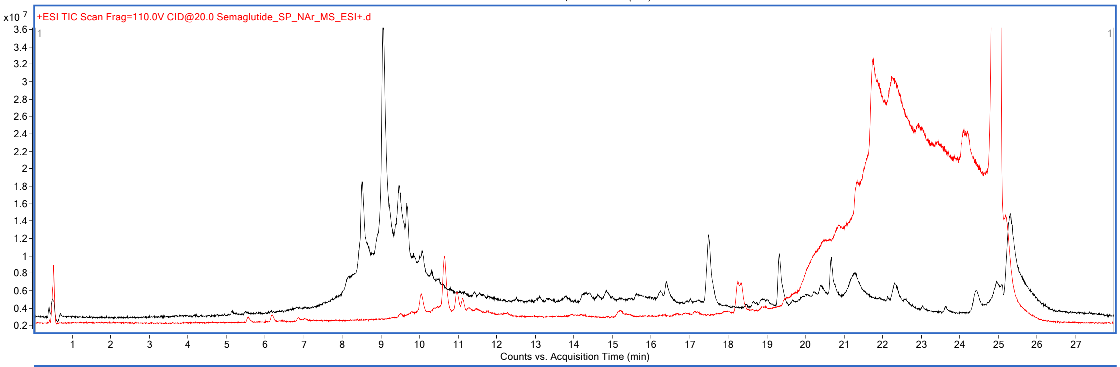
x10 7
+ESI TIC Scan Frag=110.0V CID@20.0 Semaglutide_SP_NAr_MS_ESI+.d
Counts vs. Acquisition Time (min)

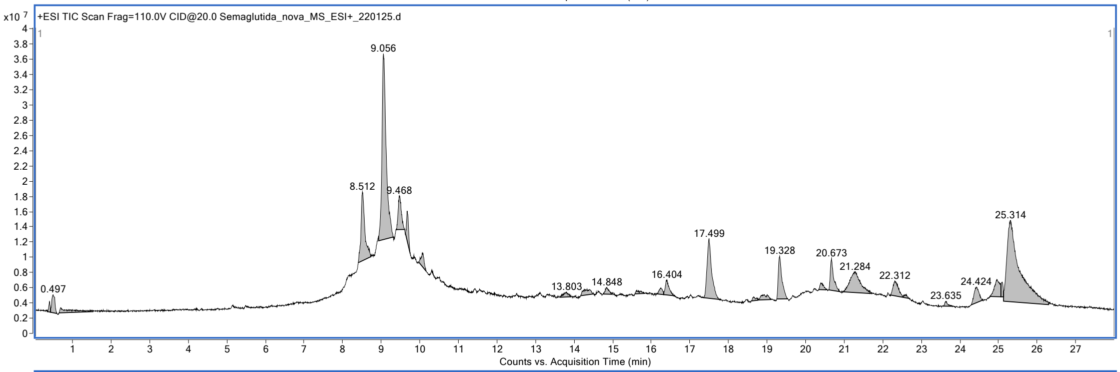
x10 7
+ESI TIC Scan Frag=110.0V CID@20.0 Semaglutida_nova_MS_ESI+_220125.d
9.056
8.512
9.468
0.497
13.803
14.848
16.404
17.499
19.328
20.673
21.284
22.312
23.635
24.424
25.314
Counts vs. Acquisition Time (min)

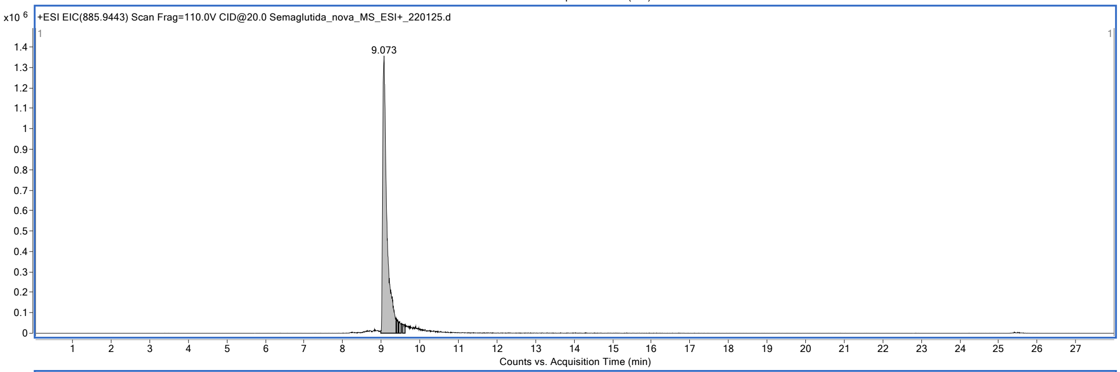
x10 6
+ESI EIC(885.9443) Scan Frag=110.0V CID@20.0 Semaglutida_nova_MS_ESI+_220125.d
9.073
Counts vs. Acquisition Time (min)

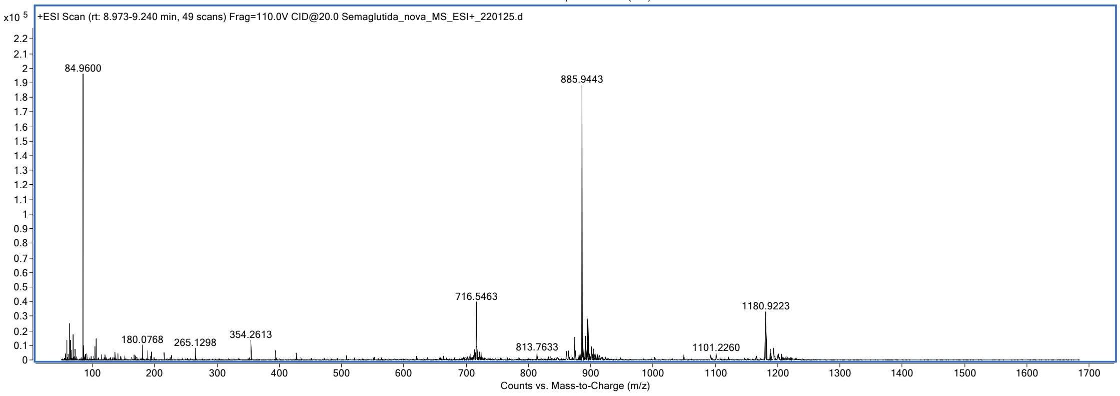
x10 5
+ESI Scan (rt: 8.973-9.240 min, 49 scans) Frag=110.0V CID@20.0 Semaglutida_nova_MS_ESI+_220125.d
84.9600
885.9443
180.0768
265.1298
354.2613
716.5463
813.7633
1101.2260
1180.9223
Counts vs. Mass-to-Charge (m/z)

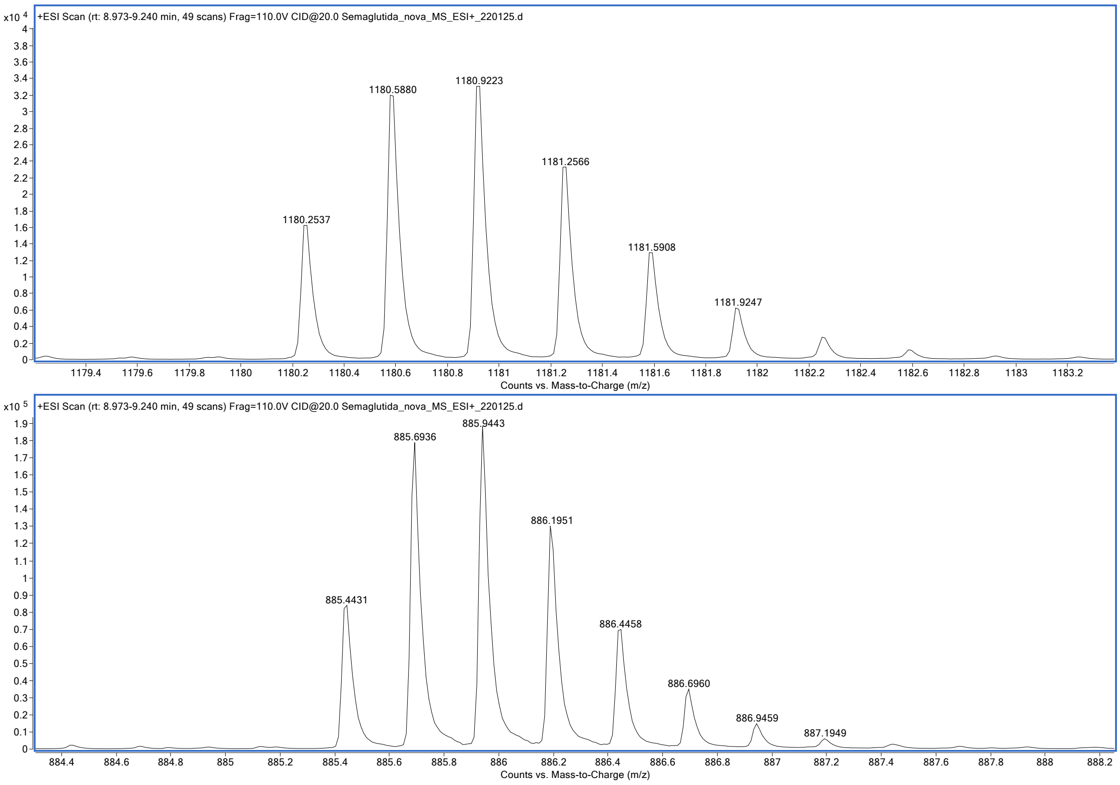

Figure 20. From top to bottom, LC-MS chromatograms (TIC, EIC) and HRMS spectra (m/z: $[M+3H]^{3+}$ Calcd. for $C_{161}H_{236}N_{44}O_{47}$ 1180.2549; Found: 1180.2537, error: -1.04 ppm, and $[M+4H]^{4+}$ Calcd. for $C_{161}H_{236}N_{44}O_{47}$ 885.4430; Found: 885.4431, error: 0.10 ppm) of the ***N***$_{\mathbf{(in)}}$**-arylated GLP-1 (7-37)** analog.

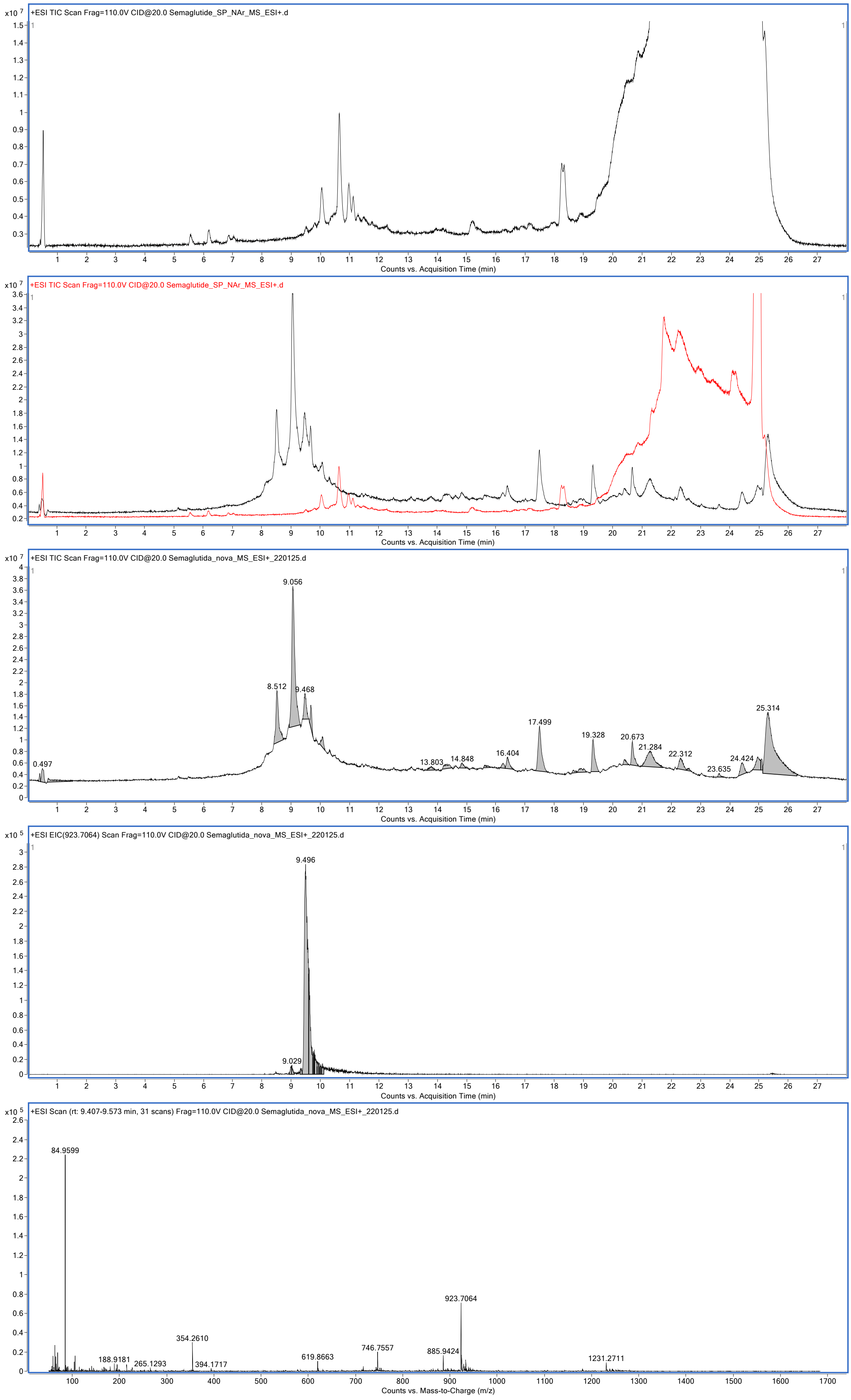
+ESI TIC Scan Frag=110.0V CID@20.0 Semaglutide_SP_NAr_MS_ESI+.d
x10 7
Counts vs. Acquisition Time (min)
+ESI TIC Scan Frag=110.0V CID@20.0 Semaglutide_SP_NAr_MS_ESI+.d
x10 7
Counts vs. Acquisition Time (min)
+ESI TIC Scan Frag=110.0V CID@20.0 Semaglutida_nova_MS_ESI+_220125.d
x10 7
9.056
8.512
9.468
0.497
13.803
14.848
16.404
17.499
19.328
20.673
21.284
22.312
23.635
24.424
25.314
Counts vs. Acquisition Time (min)
+ESI EIC(923.7064) Scan Frag=110.0V CID@20.0 Semaglutida_nova_MS_ESI+_220125.d
x10 5
9.496
9.029
Counts vs. Acquisition Time (min)
+ESI Scan (rt: 9.407-9.573 min, 31 scans) Frag=110.0V CID@20.0 Semaglutida_nova_MS_ESI+_220125.d
x10 5
84.9599
188.9181
265.1293
354.2610
394.1717
619.8663
746.7557
885.9424
923.7064
1231.2711
Counts vs. Mass-to-Charge (m/z)

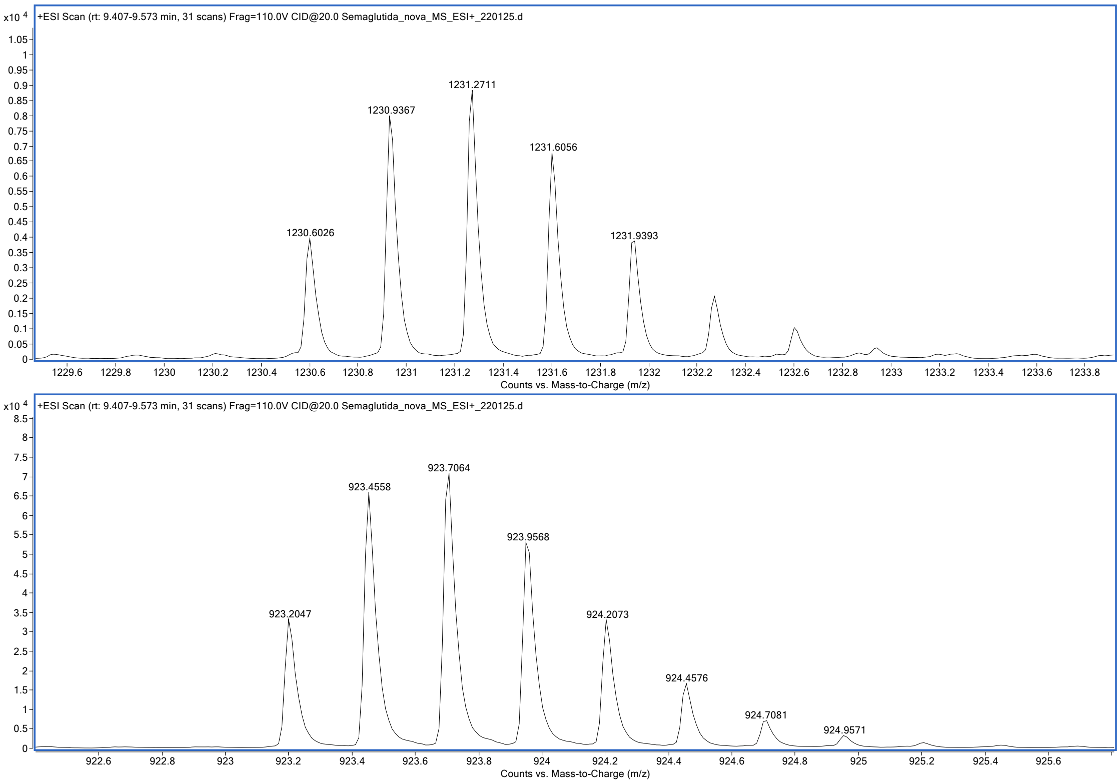

Figure 21. From top to bottom, LC-MS chromatograms (TIC, EIC) and HRMS spectra (m/z: $[M+3H]^{3+}$ Calcd. for $C_{167}H_{249}N_{43}O_{50}S$ 1230.6067; Found: 1230.6026, error: -3.37 ppm, and $[M+4H]^{4+}$ Calcd. for $C_{167}H_{249}N_{43}O_{50}S$ 923.2069; Found: 923.2047, error: -2.36 ppm) of the **(Trp_C2-Pbf) GLP-1 (7-37)** analog.

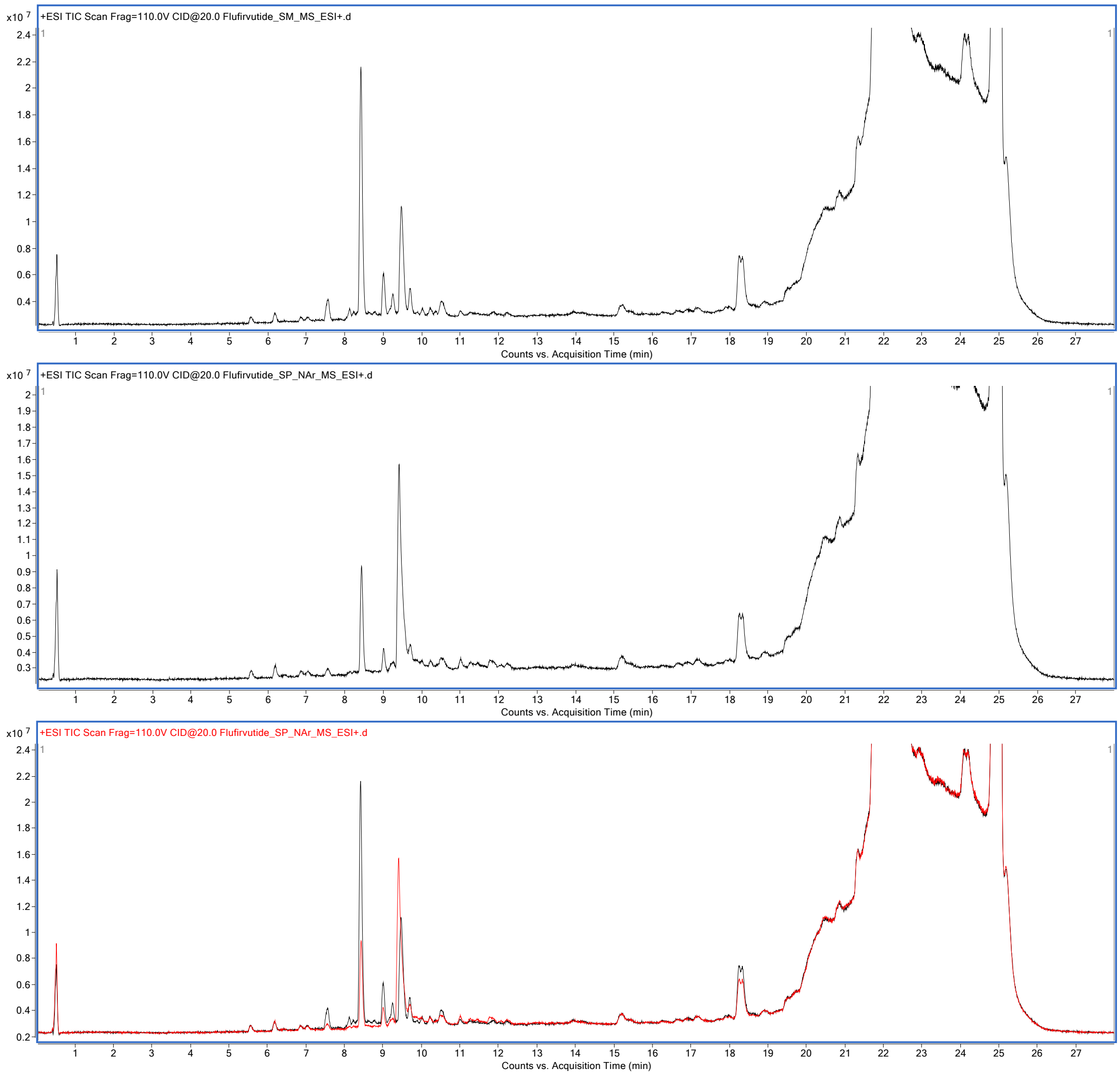

Figure 22. From top to bottom, LC-MS chromatograms (TIC) of the **Flufirvitide-3** analog, $\boldsymbol{N_{(in)}}$**-arylated Flufirvitide-3** analog, and overlapped TIC chromatograms.

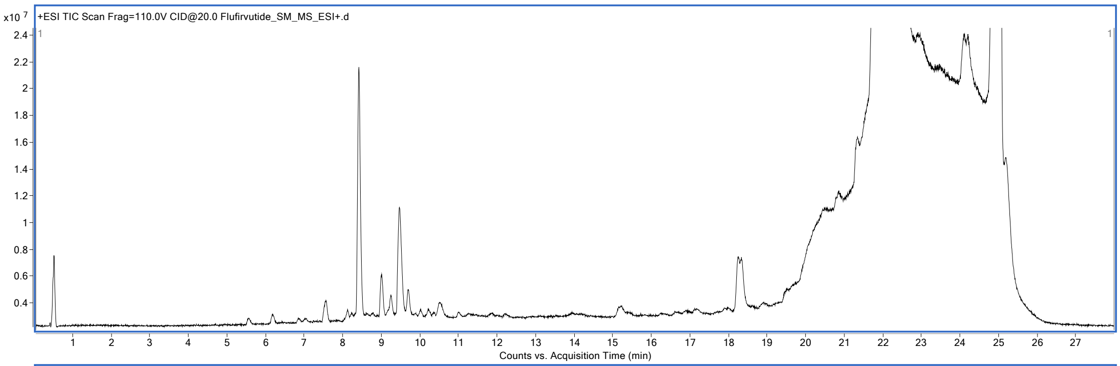
+ESI TIC Scan Frag=110.0V CID@20.0 Flufirvutide_SM_MS_ESI+.d
Counts vs. Acquisition Time (min)

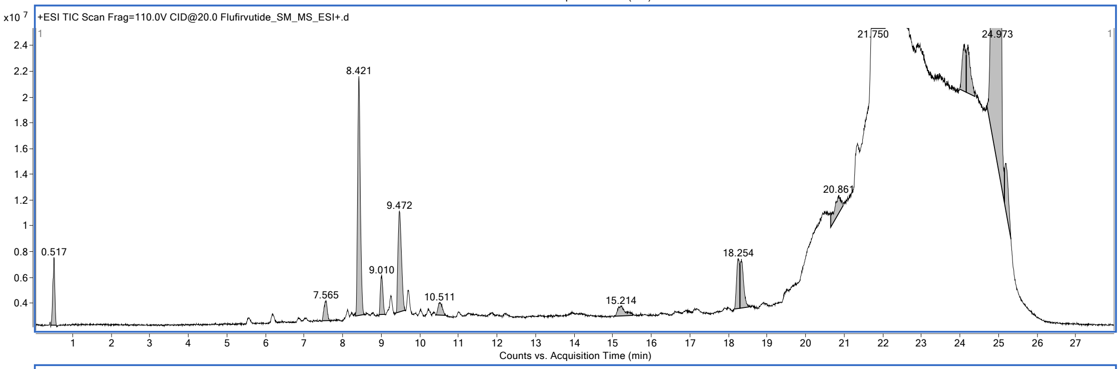
+ESI TIC Scan Frag=110.0V CID@20.0 Flufirvutide_SM_MS_ESI+.d
0.517
7.565
8.421
9.010
9.472
10.511
15.214
18.254
20.861
21.750
24.973
Counts vs. Acquisition Time (min)

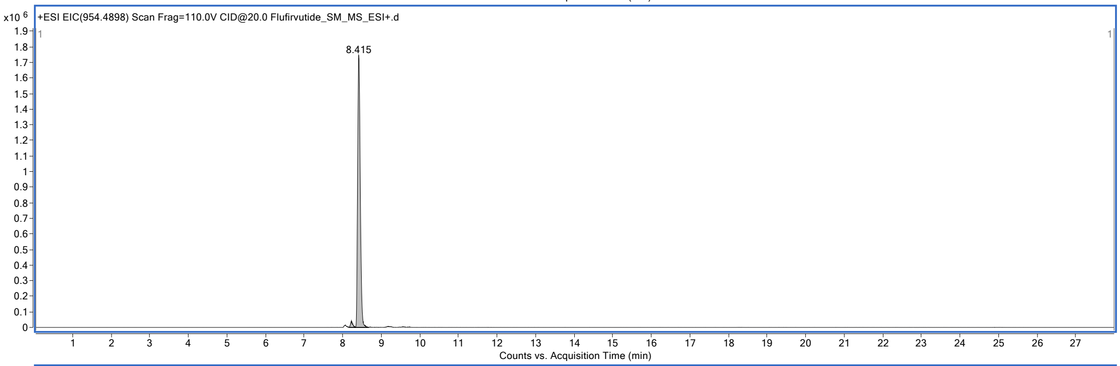
+ESI EIC(954.4898) Scan Frag=110.0V CID@20.0 Flufirvutide_SM_MS_ESI+.d
8.415
Counts vs. Acquisition Time (min)

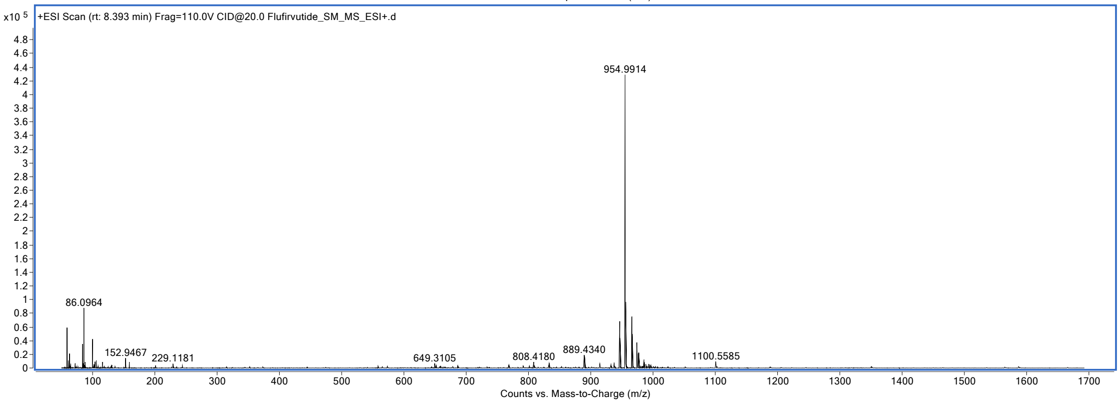
+ESI Scan (rt: 8.393 min) Frag=110.0V CID@20.0 Flufirvutide_SM_MS_ESI+.d
954.9914
86.0964
152.9467
229.1181
649.3105
808.4180
889.4340
1100.5585
Counts vs. Mass-to-Charge (m/z)

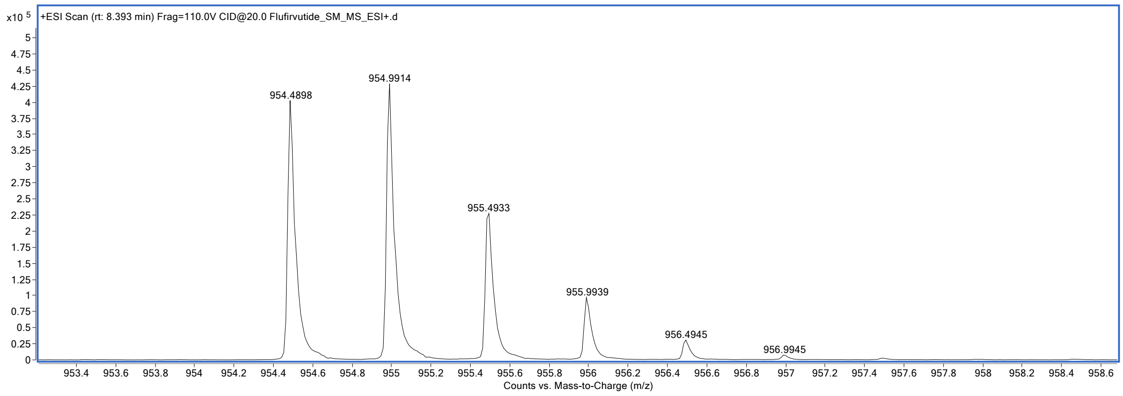

Figure 23. From top to bottom, LC-MS chromatograms (TIC, EIC) and HRMS spectra (m/z: $[M+2H]^{2+}$ Calcd. for $C_{87}H_{134}N_{20}O_{28}$ 954.4911; Found: 954.4898, error: -1.36 ppm) of the **Flufirvitide-3** analog.

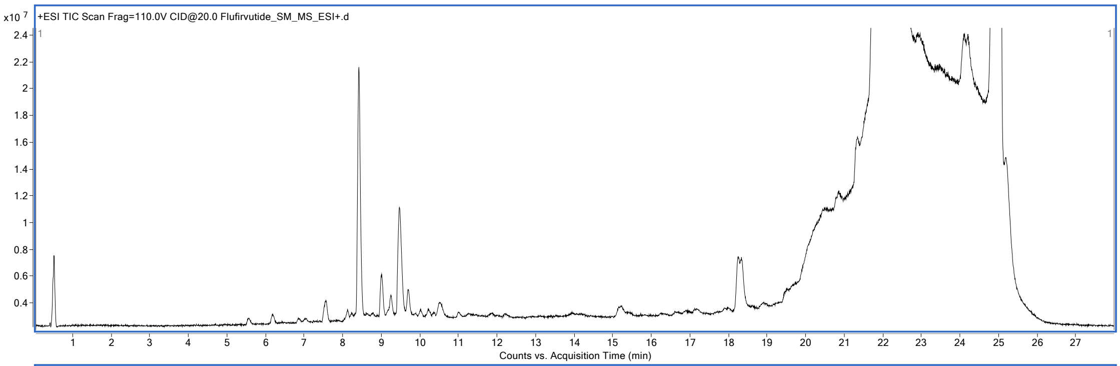
x10 7
+ESI TIC Scan Frag=110.0V CID@20.0 Flufirvutide_SM_MS_ESI+.d
Counts vs. Acquisition Time (min)

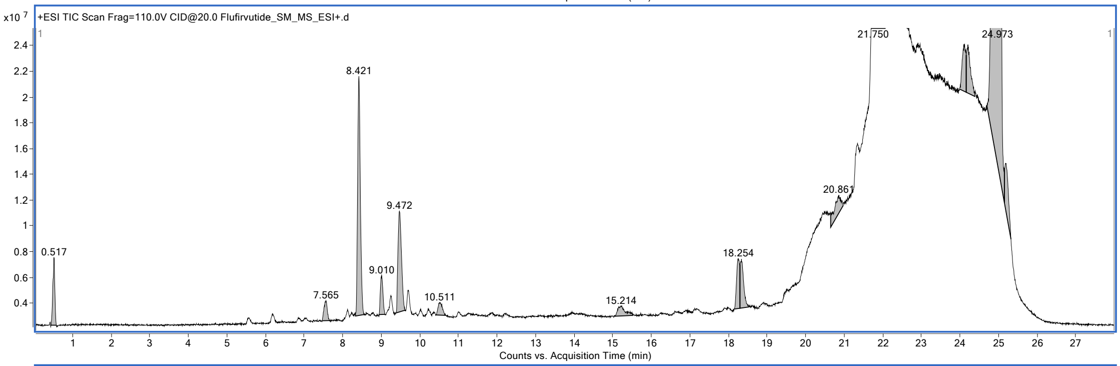
x10 7
+ESI TIC Scan Frag=110.0V CID@20.0 Flufirvutide_SM_MS_ESI+.d
0.517
7.565
8.421
9.010
9.472
10.511
15.214
18.254
20.861
21.750
24.973
Counts vs. Acquisition Time (min)

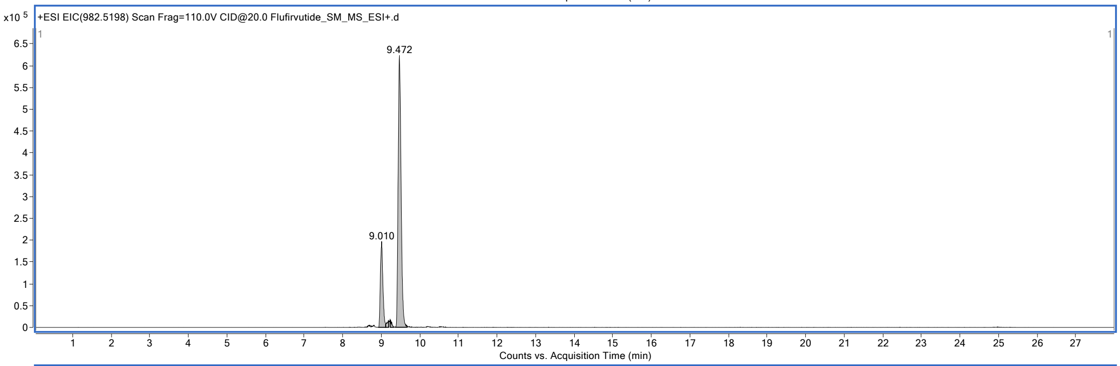
x10 5
+ESI EIC(982.5198) Scan Frag=110.0V CID@20.0 Flufirvutide_SM_MS_ESI+.d
9.472
9.010
Counts vs. Acquisition Time (min)

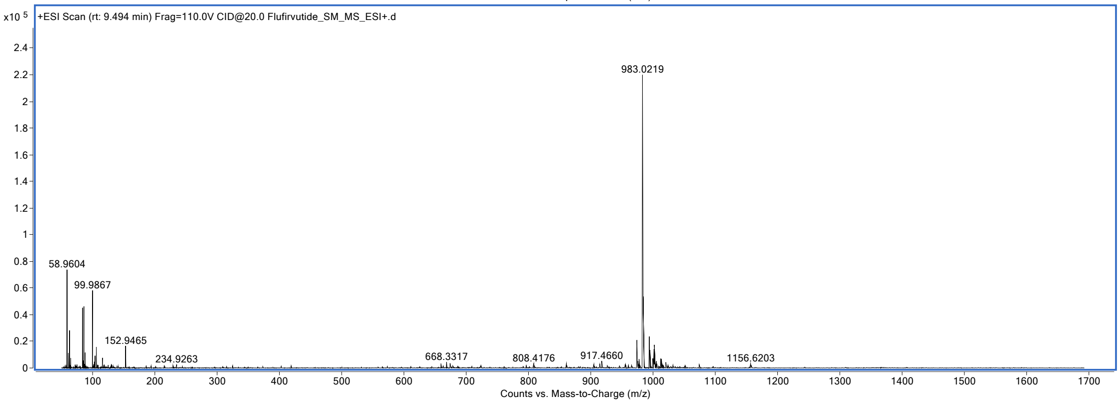
x10 5
+ESI Scan (rt: 9.494 min) Frag=110.0V CID@20.0 Flufirvutide_SM_MS_ESI+.d
983.0219
58.9604
99.9867
152.9465
234.9263
668.3317
808.4176
917.4660
1156.6203
Counts vs. Mass-to-Charge (m/z)

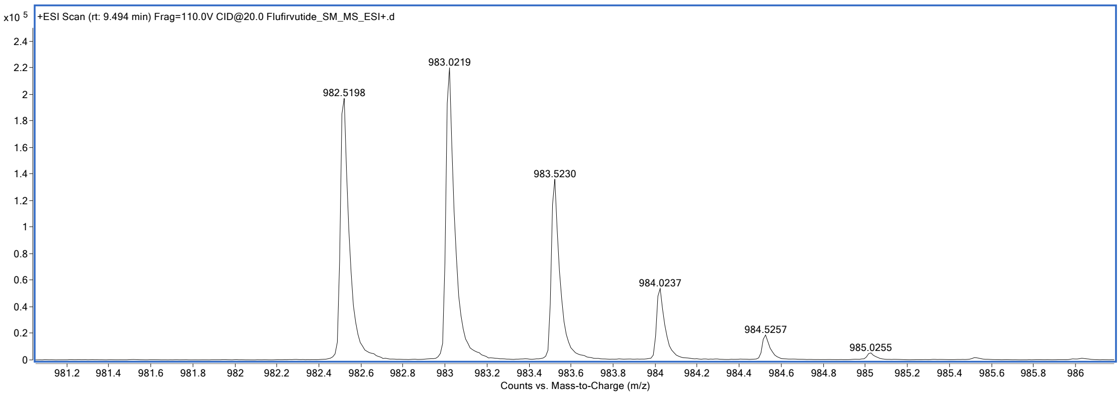

Figure 24. From top to bottom, LC-MS chromatograms (TIC, EIC) and HRMS spectra (m/z: $[M+2H]^{2+}$ Calcd. for $C_{91}H_{142}N_{20}O_{28}$ 982.5224; Found: 982.5198, error: -2.64 ppm) of the **(Trp_C2-*t*Bu) Flufirvitide-3** analog.

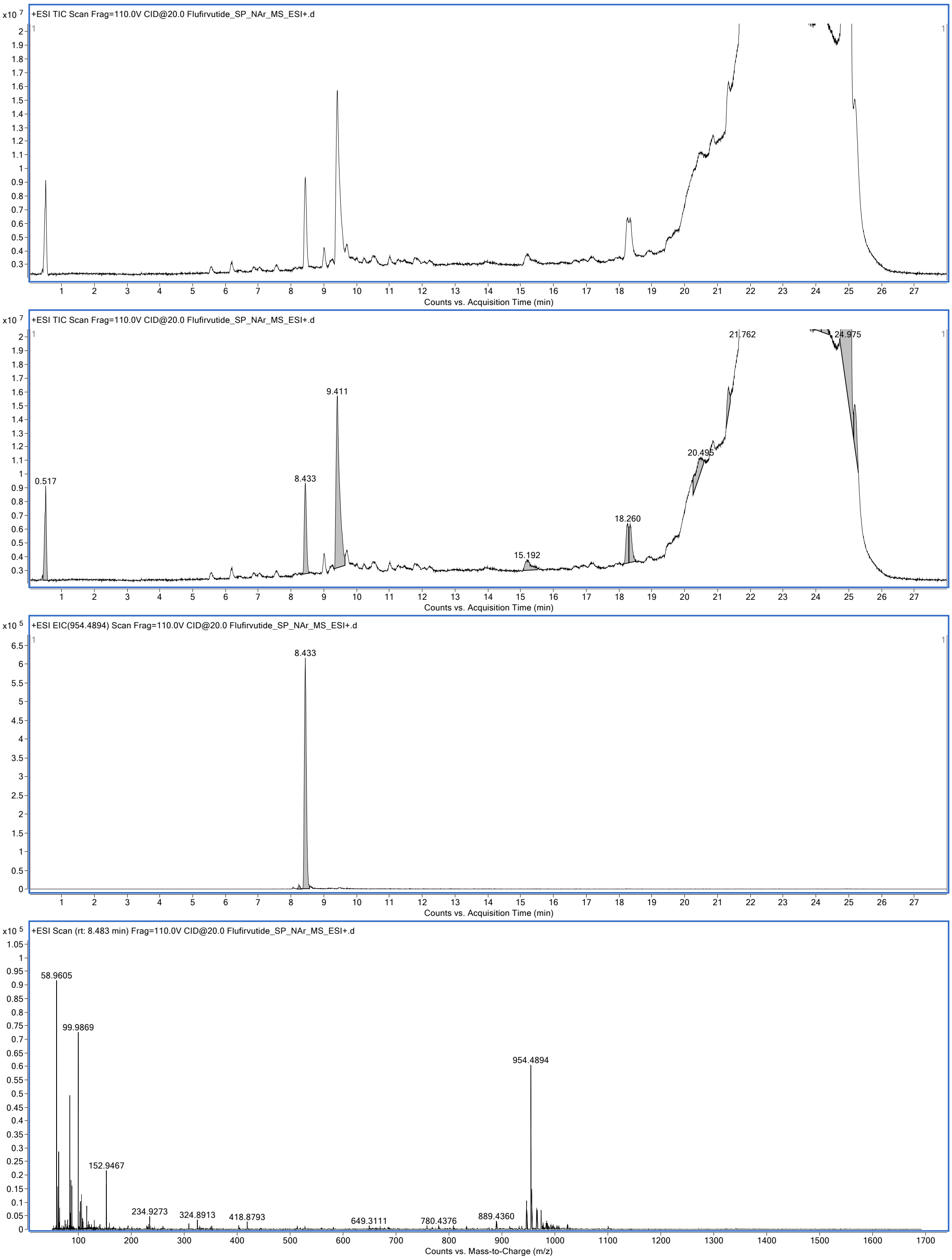
+ESI TIC Scan Frag=110.0V CID@20.0 Flufirvutide_SP_NAr_MS_ESI+.d
Counts vs. Acquisition Time (min)
+ESI TIC Scan Frag=110.0V CID@20.0 Flufirvutide_SP_NAr_MS_ESI+.d
0.517
8.433
9.411
15.192
18.260
20.495
21.762
24.975
Counts vs. Acquisition Time (min)
+ESI EIC(954.4894) Scan Frag=110.0V CID@20.0 Flufirvutide_SP_NAr_MS_ESI+.d
8.433
Counts vs. Acquisition Time (min)
+ESI Scan (rt: 8.483 min) Frag=110.0V CID@20.0 Flufirvutide_SP_NAr_MS_ESI+.d
58.9605
99.9869
152.9467
234.9273
324.8913
418.8793
649.3111
780.4376
889.4360
954.4894
Counts vs. Mass-to-Charge (m/z)

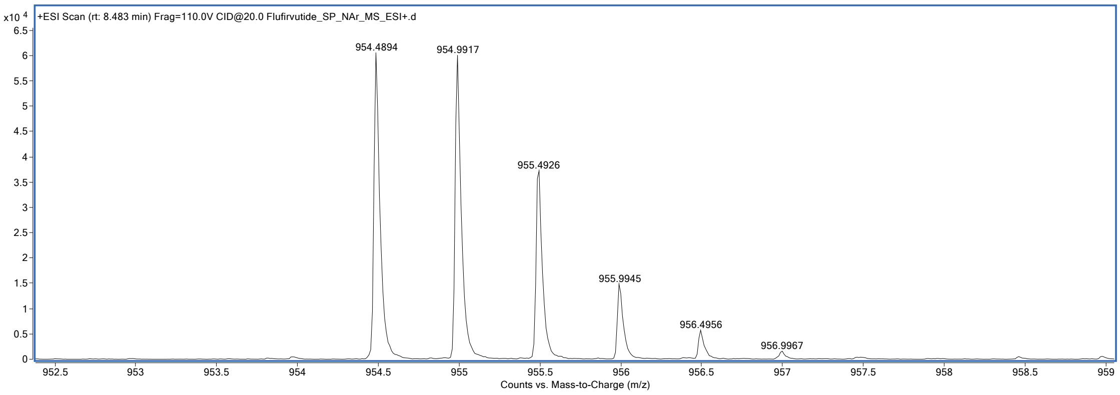

Figure 25. From top to bottom, LC-MS chromatograms (TIC, EIC) and HRMS spectra (m/z: $[M+2H]^{2+}$ Calcd. for $C_{87}H_{134}N_{20}O_{28}$ 954.4911; Found: 954.4894, error: -1.78 ppm) of the **Flufirvitide-3** analog.

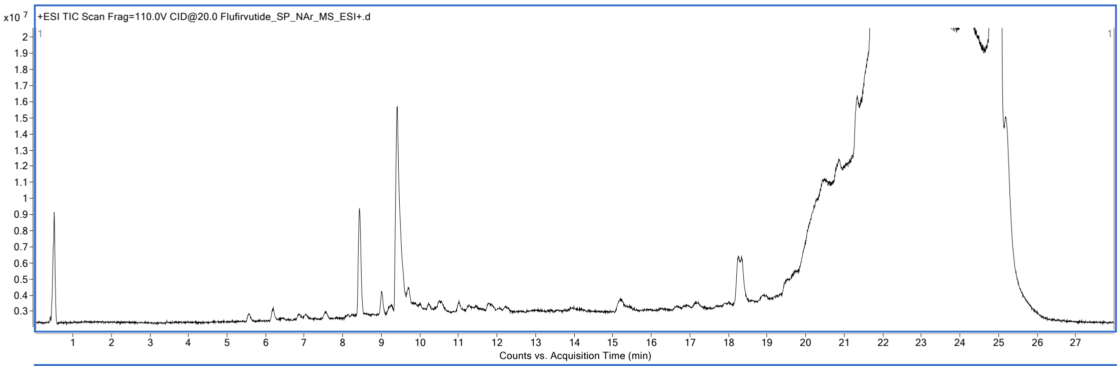
x10 7
+ESI TIC Scan Frag=110.0V CID@20.0 Flufirvutide_SP_NAr_MS_ESI+.d
Counts vs. Acquisition Time (min)

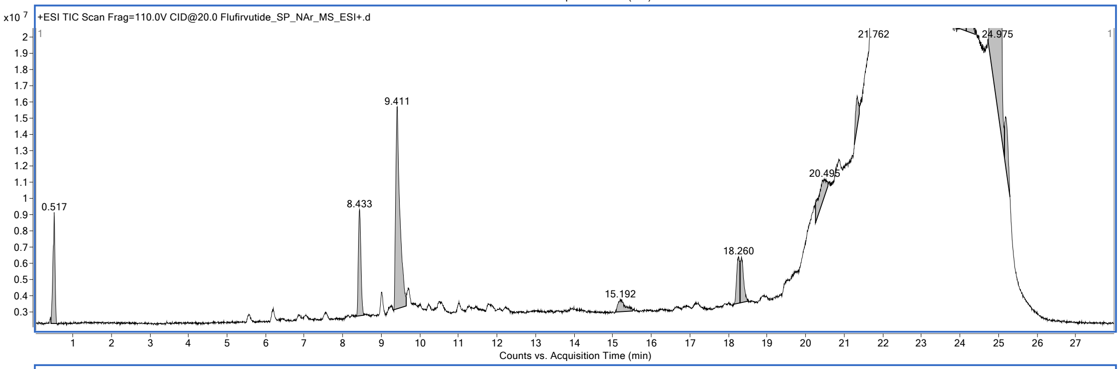
x10 7
+ESI TIC Scan Frag=110.0V CID@20.0 Flufirvutide_SP_NAr_MS_ESI+.d
0.517
8.433
9.411
15.192
18.260
20.495
21.762
24.975
Counts vs. Acquisition Time (min)

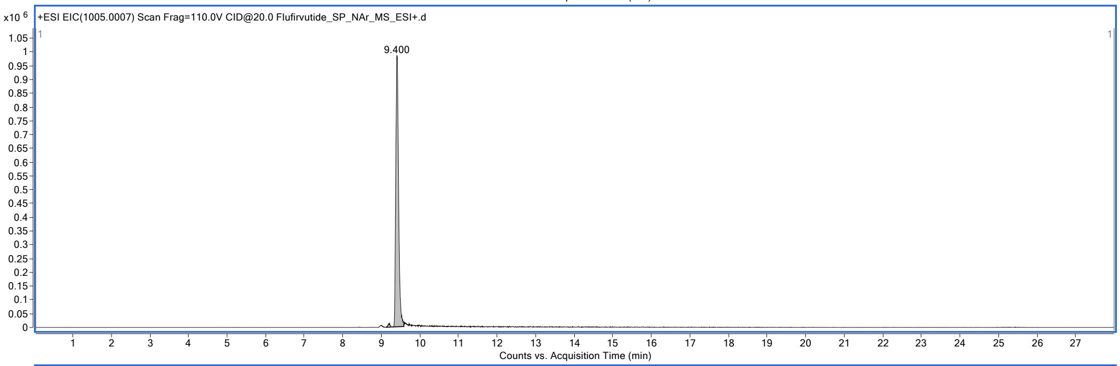
x10 6
+ESI EIC(1005.0007) Scan Frag=110.0V CID@20.0 Flufirvutide_SP_NAr_MS_ESI+.d
9.400
Counts vs. Acquisition Time (min)

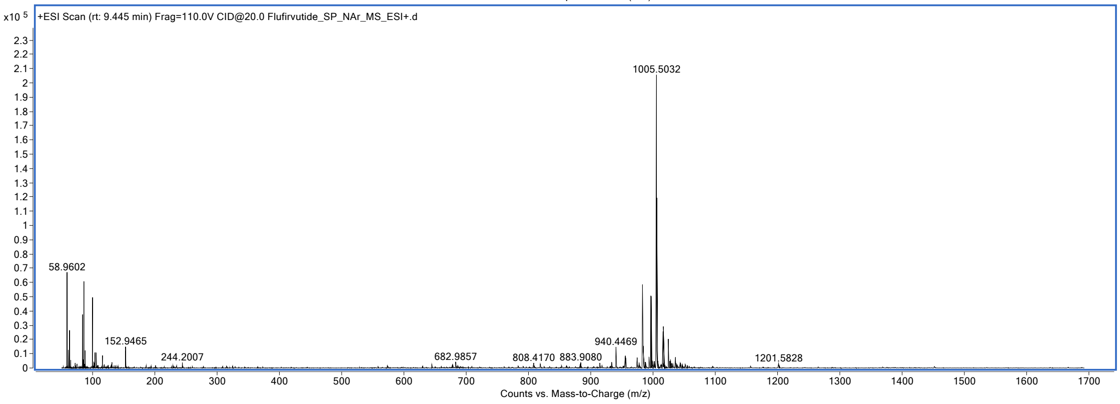
x10 5
+ESI Scan (rt: 9.445 min) Frag=110.0V CID@20.0 Flufirvutide_SP_NAr_MS_ESI+.d
1005.5032
58.9602
152.9465
244.2007
682.9857
808.4170
883.9080
940.4469
1201.5828
Counts vs. Mass-to-Charge (m/z)

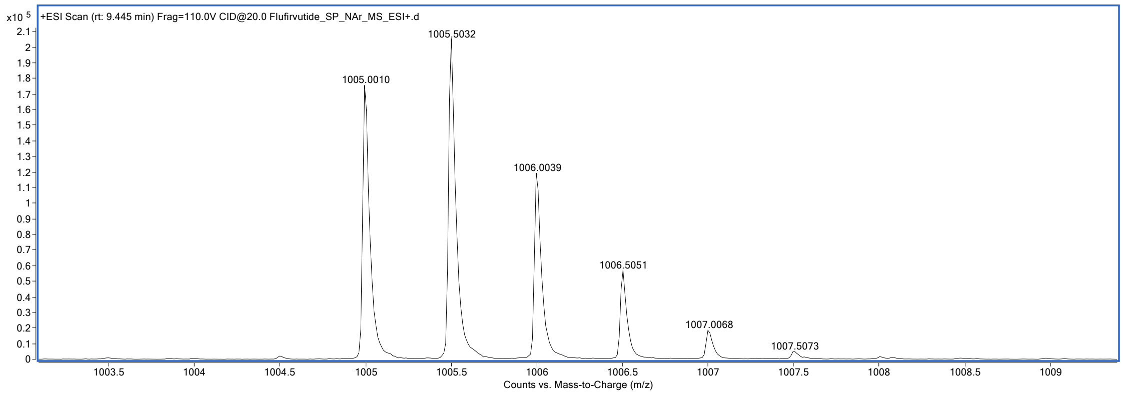

Figure 26. From top to bottom, LC-MS chromatograms (TIC, EIC) and HRMS spectra (m/z: $[M+2H]^{2+}$ Calcd. for $C_{94}H_{137}N_{21}O_{28}$ 1005.0044; Found: 1005.0010, error: -3.36 ppm) of the ***N*$_{(in)}$-arylated Flufirvitide-3** analog.

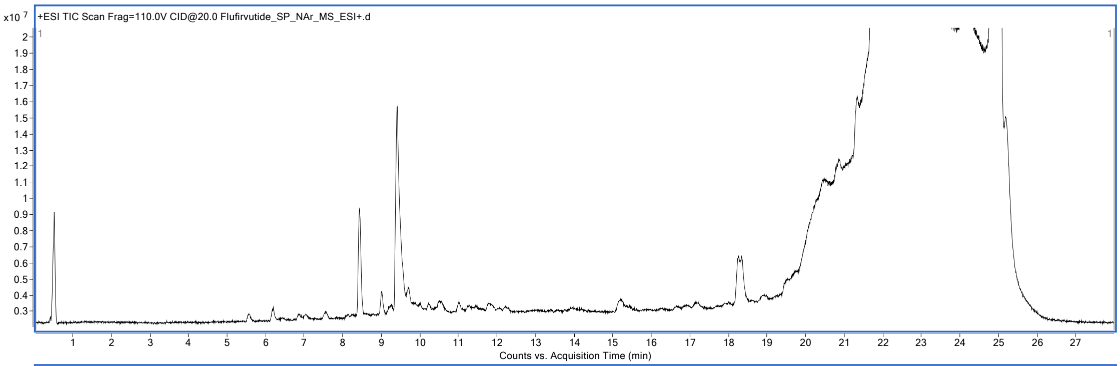
+ESI TIC Scan Frag=110.0V CID@20.0 Flufirvutide_SP_NAr_MS_ESI+.d
x10 7
Counts vs. Acquisition Time (min)

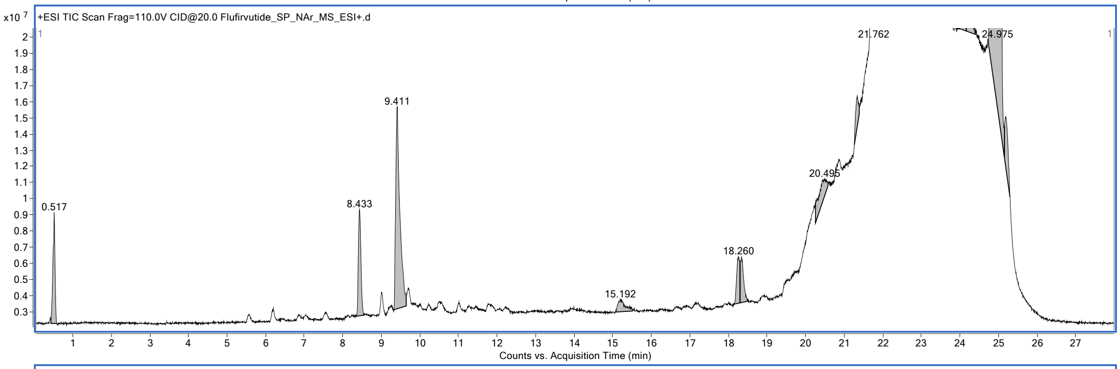
+ESI TIC Scan Frag=110.0V CID@20.0 Flufirvutide_SP_NAr_MS_ESI+.d
x10 7
0.517
8.433
9.411
15.192
18.260
20.495
21.762
24.975
Counts vs. Acquisition Time (min)

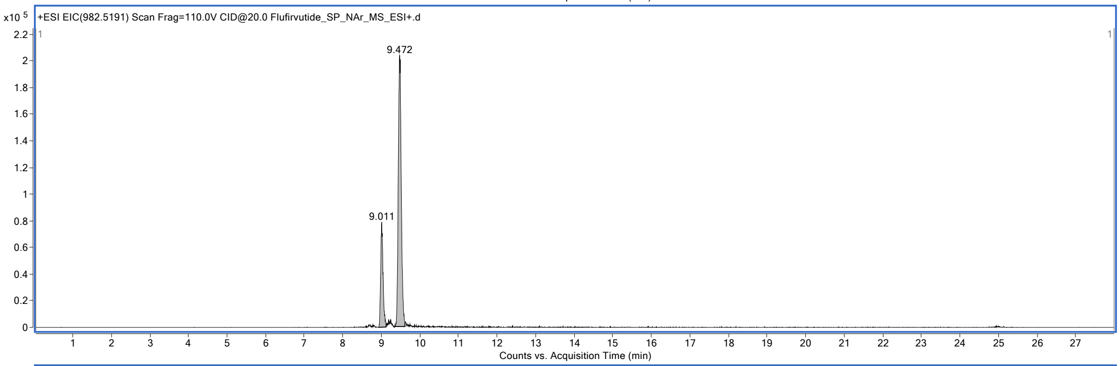
+ESI EIC(982.5191) Scan Frag=110.0V CID@20.0 Flufirvutide_SP_NAr_MS_ESI+.d
x10 5
9.472
9.011
Counts vs. Acquisition Time (min)

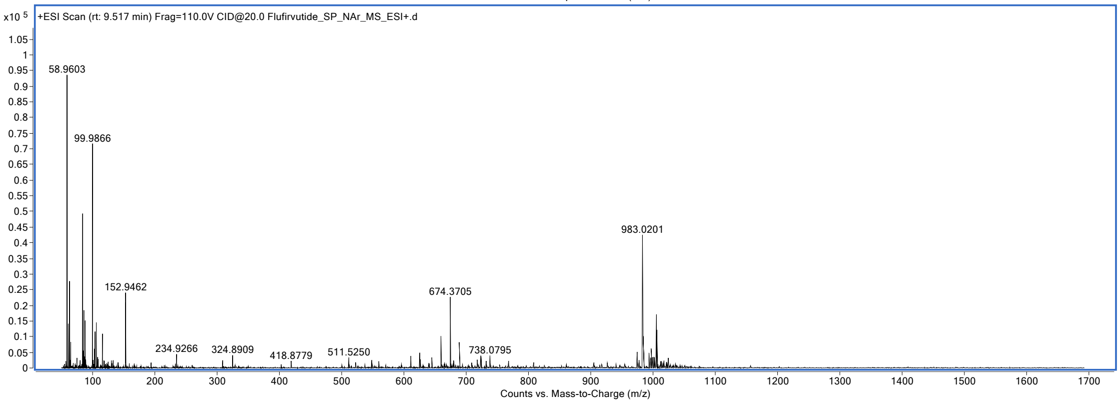
+ESI Scan (rt: 9.517 min) Frag=110.0V CID@20.0 Flufirvutide_SP_NAr_MS_ESI+.d
x10 5
58.9603
99.9866
152.9462
234.9266
324.8909
418.8779
511.5250
674.3705
738.0795
983.0201
Counts vs. Mass-to-Charge (m/z)

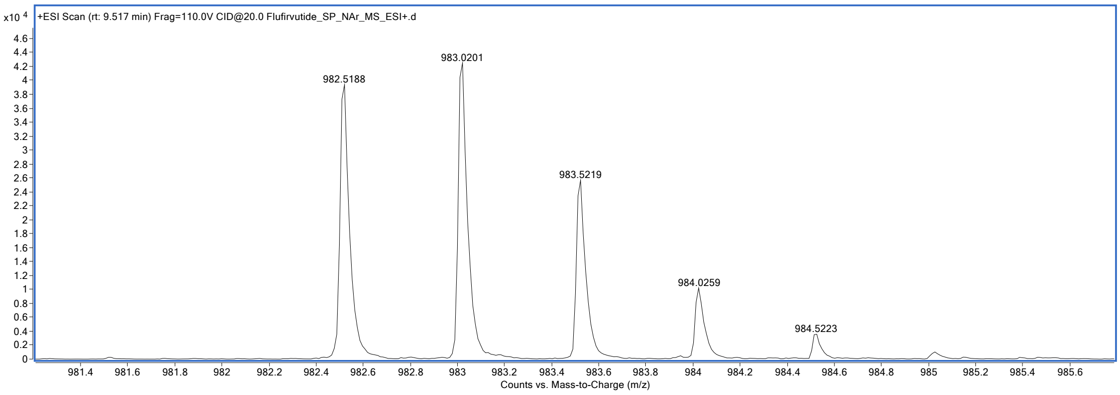

Figure 27. From top to bottom, LC-MS chromatograms (TIC, EIC) and HRMS spectra (m/z: $[M+2H]^{2+}$ Calcd. for $C_{91}H_{142}N_{20}O_{28}$ 982.5224; Found: 982.5188, error: -3.66 ppm) of the **(Trp_C2-*t*Bu) Flufirvitide-3** analog.

8. Analytical RP-HPLC, LC-MS/MS and HRMS Data of pure peptides

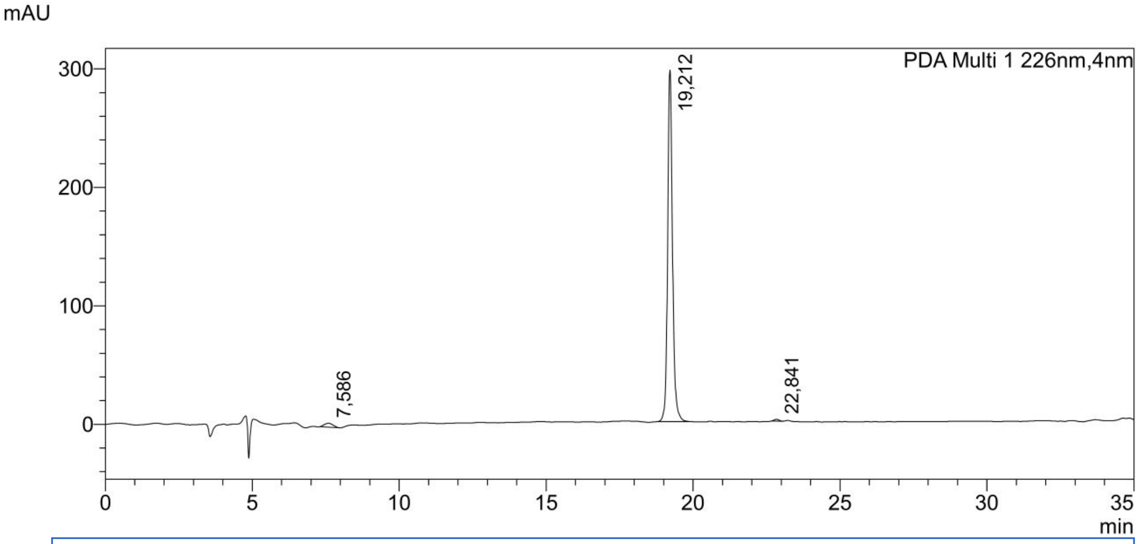

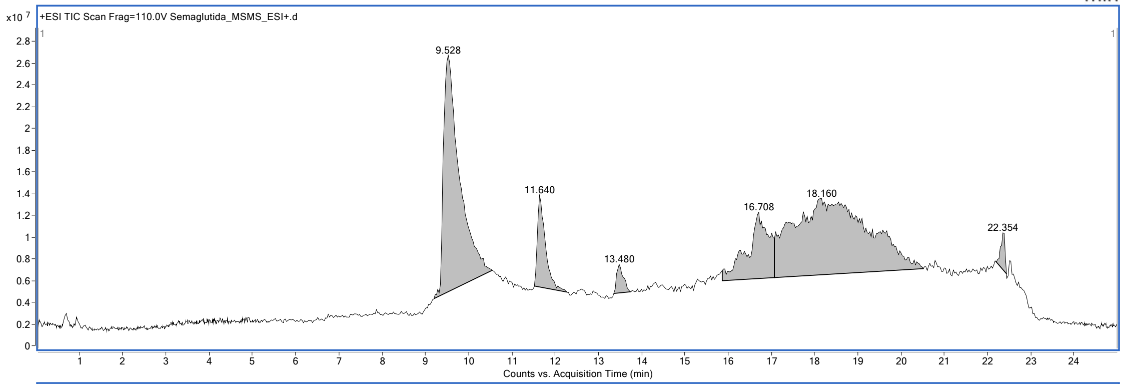

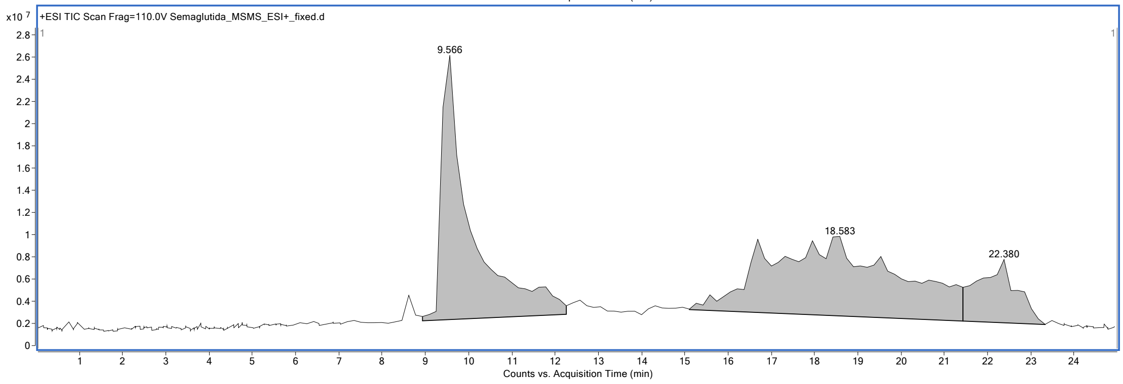

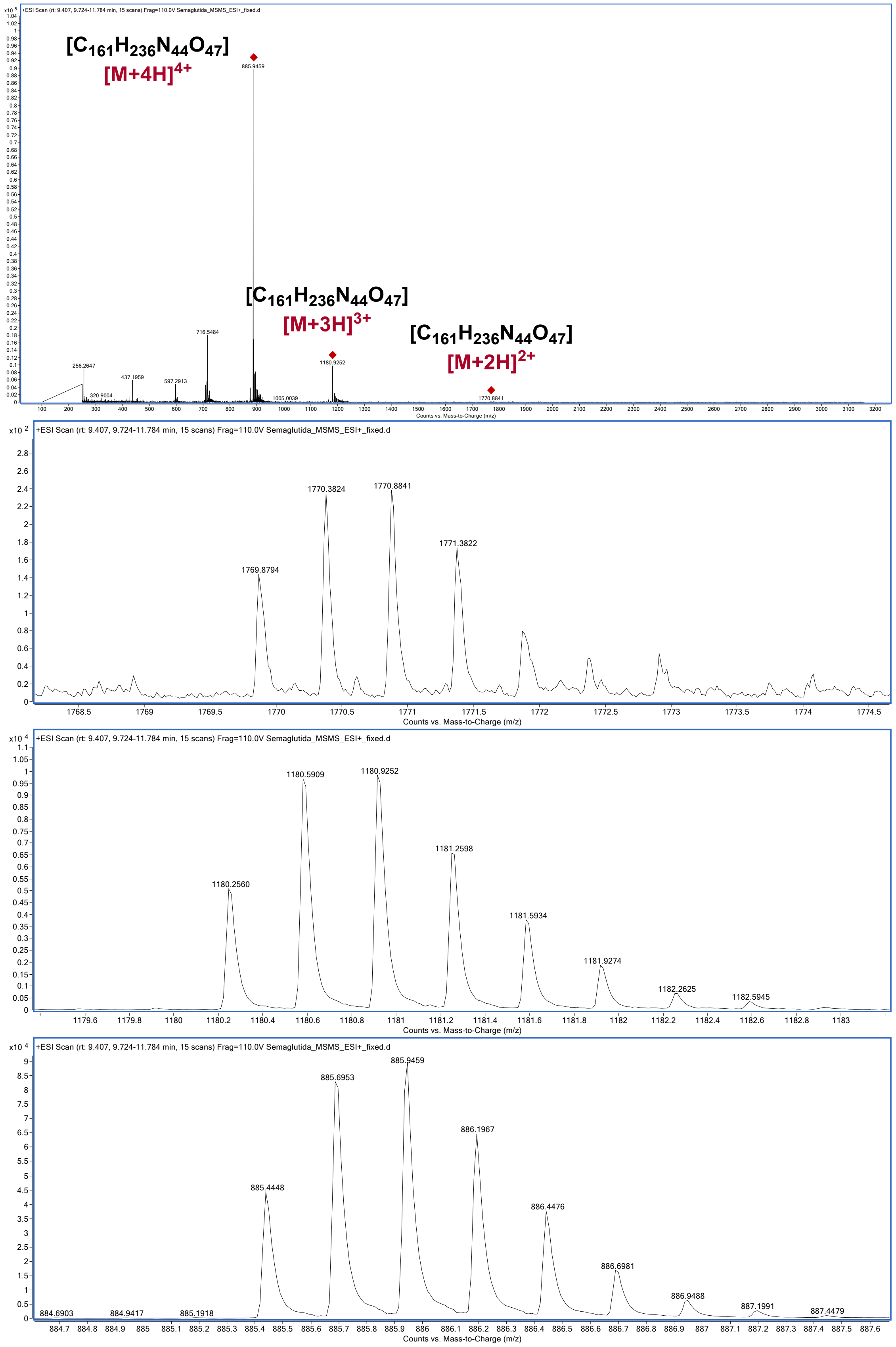

Figure 28. From top to bottom, RP-HPLC chromatogram (97% purity, retention time 19.212 min), LC-MS (TIC, full scan) chromatogram, LC-MS/MS chromatogram and HRMS spectra (m/z: $[M+2H]^{2+}$ Calcd. for $C_{161}H_{236}N_{44}O_{47}$ 1769.8788; Found: 1769.8794, error: 0.37 ppm, $[M+3H]^{3+}$ Calcd. for $C_{161}H_{236}N_{44}O_{47}$ 1180.2549; Found: 1180.2560, error: 0.91 ppm, and $[M+4H]^{4+}$ Calcd.

for $C_{161}H_{236}N_{44}O_{47}$ 885.4430; Found: 885.4448, error: 2.02 ppm) of the ***N*(in)-arylated GLP-1 (7-37)** analog.

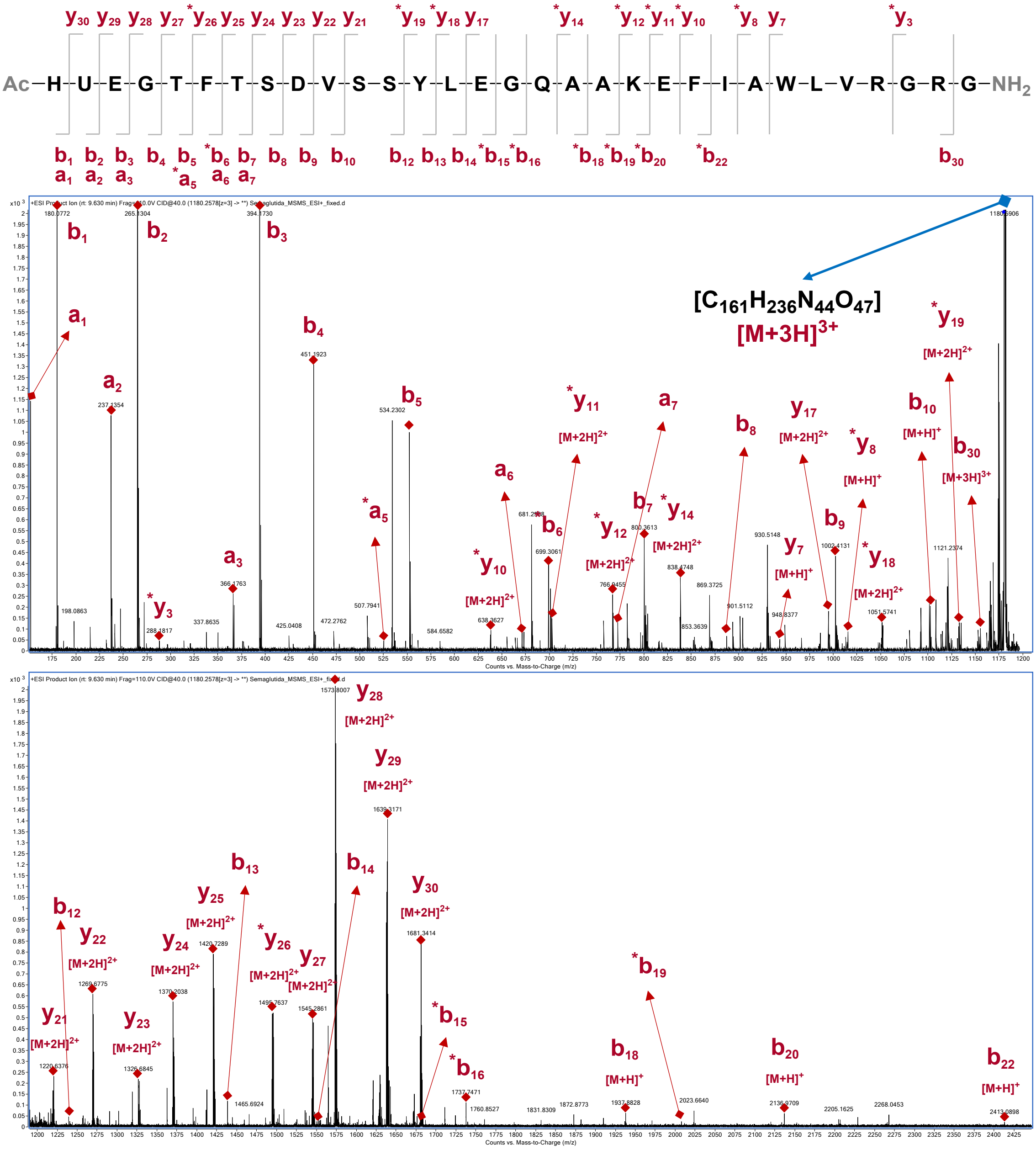

Figure 29. From top to bottom, schematic representation of fragmentation pathways and ions detected, and assigned MS/MS spectra of the ***N*(in)-arylated GLP-1 (7-37)** analog. *** ions detected with a difference of error ≥ 5 ppm.**

$N_{(in)}$-arylated GLP-1 (7-37) analog- b_x Ion Mass	$N_{(in)}$-arylated GLP-1 (7-37) analog- y_x Ion Mass	$N_{(in)}$-arylated GLP-1 (7-37) analog- a_x Ion Mass
b_1-Chemical Formula: C8H10N3O2+<br>Exact Mass: 180.0768 M+ 180.0772	y_{30}-Chemical Formula: C153H228N41O45+<br>Exact Mass: 3359.6808 [M+2H]2+ 1680.8505	a_1-Chemical Formula: C7H10N3O+<br>Exact Mass: 152.0818 M+ 152.0822
b_2-Chemical Formula: C12H17N4O3+<br>Exact Mass: 265.1295 M+ 265.1304	y_{29}-Chemical Formula: C149H221N40O44+<br>Exact Mass: 3274.6280 [M+2H]2+ 1638.3251	a_2-Chemical Formula: C11H17N4O2+<br>Exact Mass: 237.1346 M+ 237.1354
b_3-Chemical Formula: C17H24N5O6+<br>Exact Mass: 394.1721 M+ 394.17300	y_{28}-Chemical Formula: C144H214N39O41+<br>Exact Mass: 3145.5854 [M+2H]2+ 1573.8007	a_3-Chemical Formula: C16H24N5O5+<br>Exact Mass: 366.1772 M+ 366.1763
b_4-Chemical Formula: C19H27N6O7+<br>Exact Mass: 451.1936 M+ 451.1923	y_{27}-Chemical Formula: C142H211N38O40+<br>Exact Mass: 3088.5639 [M+2H]2+ 1545.2861	a_4-Chemical Formula: C18H27N6O6+<br>Exact Mass: 423.1987 ----------------------
b_5-Chemical Formula: C23H34N7O9+<br>Exact Mass: 552.2413 M+ 552.2406	y_{26}-Chemical Formula: C138H204N37O38+<br>Exact Mass: 2987.5163 [M+2H]2+ 1494.7581*	a_5-Chemical Formula: C22H34N7O8+<br>Exact Mass: 524.2463 [M+H]+ 525.2577*
b_6-Chemical Formula: C32H43N8O10+<br>Exact Mass: 699.3097 M+ 699.3061*	y_{25}-Chemical Formula: C129H195N36O37+<br>Exact Mass: 2840.4478 [M+2H]2+ 1421.2312	a_6-Chemical Formula: C31H43N8O9+<br>Exact Mass: 671.3148 M+ 671.3140
b_7-Chemical Formula: C36H50N9O12+<br>Exact Mass: 800.3573 M+ 800.3613	y_{24}-Chemical Formula: C125H188N35O35+<br>Exact Mass: 2739.4002 [M+2H]2+ 1370.7041	a_7-Chemical Formula: C35H50N9O11+<br>Exact Mass: 772.3624 M+ 772.3650
b_8-Chemical Formula: C39H55N10O14+<br>Exact Mass: 887.3894 M+ 887.3905	y_{23}-Chemical Formula: C122H183N34O33+<br>Exact Mass: 2652.3681 [M+2H]2+ 1327.1868	
b_9-Chemical Formula: C43H60N11O17+<br>Exact Mass: 1002.4163 M+ 1002.4131	y_{22}-Chemical Formula: C118H178N33O30+<br>Exact Mass: 2537.3412 [M+2H]2+ 1269.6775	
b_{10}-Chemical Formula: C48H69N12O18+<br>Exact Mass: 1101.4847 [M+H]+ 1102.4887	y_{21}-Chemical Formula: C113H169N32O29+<br>Exact Mass: 2438.2728 [M+2H]2+ 1220.1441	
b_{11}-Chemical Formula: C51H74N13O20+<br>Exact Mass: 1188.5168 ----------------------	y_{20}-Chemical Formula: C110H164N31O27+<br>Exact Mass: 2351.2407 ----------------------	
b_{12}-Chemical Formula: C54H79N14O22+<br>Exact Mass: 1275.5488 M+ 1275.5538	y_{19}-Chemical Formula: C107H159N30O25+<br>Exact Mass: 2264.2087 [M+2H]2+ 1133.1021*	
b_{13}-Chemical Formula: C63H88N15O24+<br>Exact Mass: 1438.6121 M+ 1438.6124	y_{18}-Chemical Formula: C98H150N29O23+<br>Exact Mass: 2101.1454 [M+2H]2+ 1051.5741*	
b_{14}-Chemical Formula: C69H99N16O25+<br>Exact Mass: 1551.6962 M+ 1551.6904	y_{17}-Chemical Formula: C92H139N28O22+<br>Exact Mass: 1988.0613 [M+2H]2+ 995.0360	
b_{15}-Chemical Formula: C74H106N17O28+<br>Exact Mass: 1680.7388 M+ 1680.7676*	y_{16}-Chemical Formula: C87H132N27O19+<br>Exact Mass: 1859.0187 ----------------------	
b_{16}-Chemical Formula: C76H109N18O29+<br>Exact Mass: 1737.7602 M+ 1737.7471*	y_{15}-Chemical Formula: C85H129N26O18+<br>Exact Mass: 1801.9973 ----------------------	
b_{17}-Chemical Formula: C81H117N20O31+<br>Exact Mass: 1865.8188 ----------------------	y_{14}-Chemical Formula: C80H121N24O16+<br>Exact Mass: 1673.9387 [M+2H]2+ 837.9687*	
b_{18}-Chemical Formula: C84H122N21O32+<br>Exact Mass: 1936.8559 [M+H]+ 1937.8828*	y_{13}-Chemical Formula: C77H116N23O15+<br>Exact Mass: 1602.9016 ----------------------	
b_{19}-Chemical Formula: C87H127N22O33+<br>Exact Mass: 2007.8930 M+ 2007.8757*	y_{12}-Chemical Formula: C74H111N22O14+<br>Exact Mass: 1531.8645 [M+2H]2+ 766.9455*	
b_{20}-Chemical Formula: C93H139N24O34<br>Exact Mass: 2135.9886 [M+H]+ 2136.9709*	y_{11}-Chemical Formula: C68H99N20O13+<br>Exact Mass: 1403.7695 [M+2H]2+ 702.88580*	
b_{21}-Chemical Formula: C98H146N25O37<br>Exact Mass: 2265.0311 ----------------------	y_{10}-Chemical Formula: C63H92N19O10+<br>Exact Mass: 1274.7269 [M+2H]2+ 638.3627*	
b_{22}-Chemical Formula: C107H155N26O38+<br>Exact Mass: 2412.0990 [M+H]+ 2413.0898*	y_9-Chemical Formula: C54H83N18O9+<br>Exact Mass: 1127.6585 ----------------------	
b_{23}-Chemical Formula: C113H166N27O39<br>Exact Mass: 2525.1836 ----------------------	y_8-Chemical Formula: C48H72N17O8+<br>Exact Mass: 1014.5744 [M+H]+ 1015.5712*	
b_{24}-Chemical Formula: C116H171N28O40+<br>Exact Mass: 2596.2202 ----------------------	y_7-Chemical Formula: C45H67N16O7+<br>Exact Mass: 943.5373 [M+H]+ 943.5398	
b_{25}-Chemical Formula: C134H184N31O41+<br>Exact Mass: 2883.3261 ----------------------	y_6-Chemical Formula: C27H54N13O6+<br>Exact Mass: 656.4315 ----------------------	
b_{26}-Chemical Formula: C140H195N32O42+<br>Exact Mass: 2996.4101 ----------------------	y_5-Chemical Formula: C21H43N12O5+<br>Exact Mass: 543.3474 ----------------------	
b_{27}-Chemical Formula: C145H204N33O43+<br>Exact Mass: 3095.4785 ----------------------	y_4-Chemical Formula: C16H34N11O4+<br>Exact Mass: 444.2790 ----------------------	
b_{28}-Chemical Formula: C151H216N37O44+<br>Exact Mass: 3251.5796 ----------------------	y_3-Chemical Formula: C10H22N7O3+<br>Exact Mass: 288.1779 M+ 288.1817*	
b_{29}-Chemical Formula: C153H219N38O45+<br>Exact Mass: 3308.6011 ----------------------	y_2-Chemical Formula: C8H19N6O2+<br>Exact Mass: 231.1564 ----------------------	
b_{30}-Chemical Formula: C159H231N42O46+<br>Exact Mass: 3464.7022 [M+3H]3+ 1155.9042	y_1-Chemical Formula: C2H7N2O+<br>Exact Mass: 75.0553 ----------------------	

Table 1. Summarized data values for detected ions type b_x-y_x, and a_x ions. *** ions detected with a difference of error ≥ 5 ppm.**

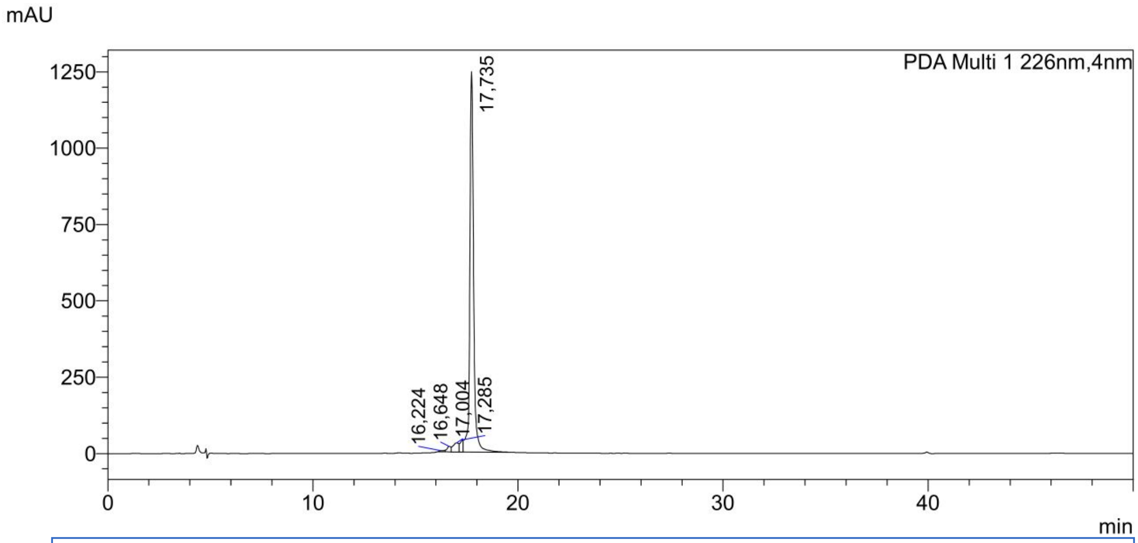
mAU
PDA Multi 1 226nm,4nm
1250
1000
750
500
250
0
17,735
16,224
16,648
17,004
17,285
0
10
20
30
40
min

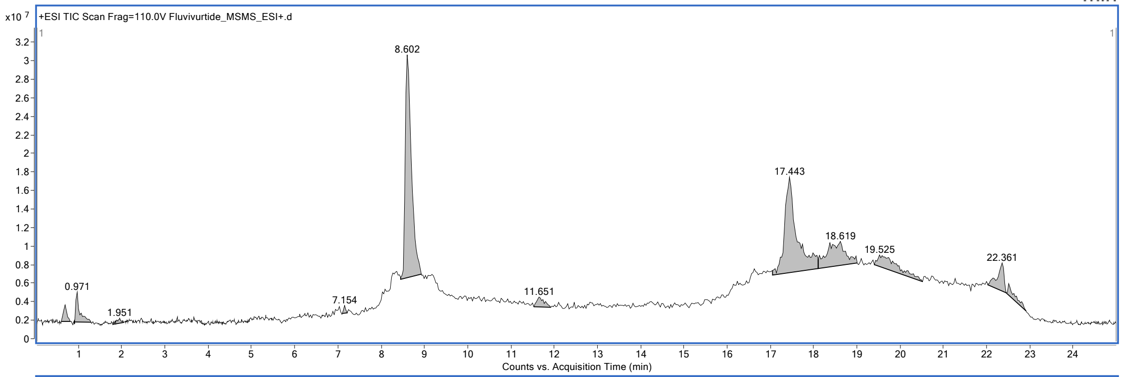
x10 7
+ESI TIC Scan Frag=110.0V Fluvivurtide_MSMS_ESI+.d
8.602
17.443
18.619
19.525
22.361
0.971
1.951
7.154
11.651
Counts vs. Acquisition Time (min)

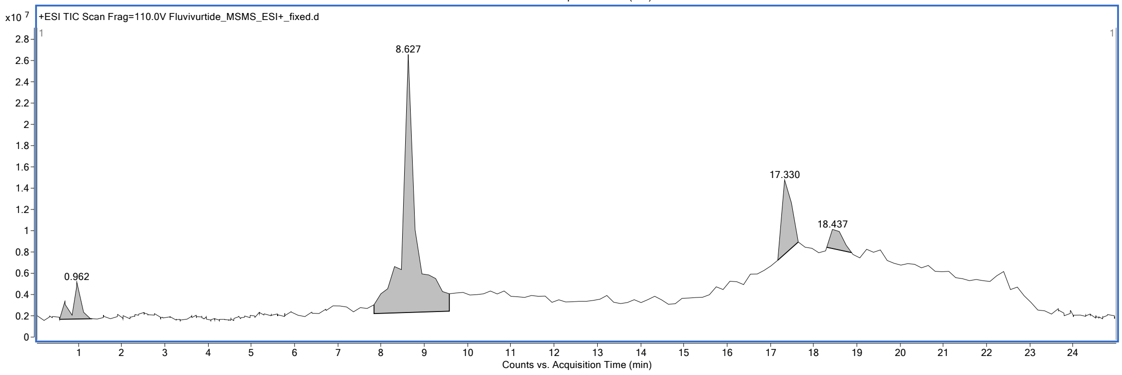
x10 7
+ESI TIC Scan Frag=110.0V Fluvivurtide_MSMS_ESI+_fixed.d
8.627
17.330
18.437
0.962
Counts vs. Acquisition Time (min)

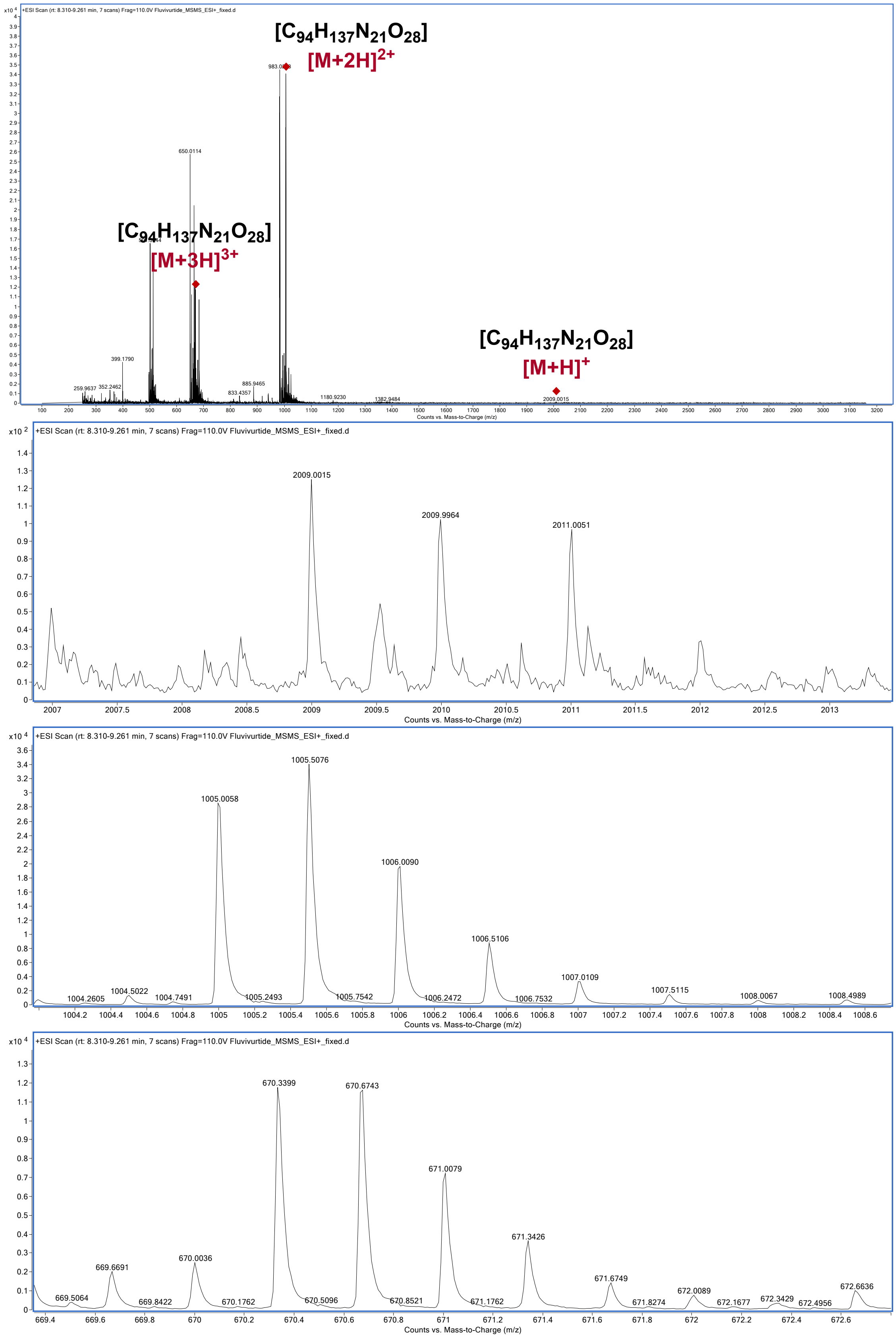

Figure 30. From top to bottom, RP-HPLC chromatogram (93% purity, retention time 17.735 min), LC-MS (TIC, full scan) chromatogram, LC-MS/MS chromatogram and HRMS spectra (m/z: $[M+H]^+$ Calcd. for $C_{94}H_{137}N_{21}O_{28}$ 2009.0015; Found: 2009.0015, error: 0.02 ppm, $[M+2H]^{2+}$ Calcd. for $C_{161}H_{236}N_{44}O_{47}$ 1005.0044; Found: 1005.0058, error: 1.42 ppm, and $[M+3H]^{3+}$ Calcd. for $C_{161}H_{236}N_{44}O_{47}$ 670.3387; Found: 670.3399, error: 1.83 ppm) of the ***N*$_{(in)}$-arylated Flufirvitide-3** analog.

9. NMR data.

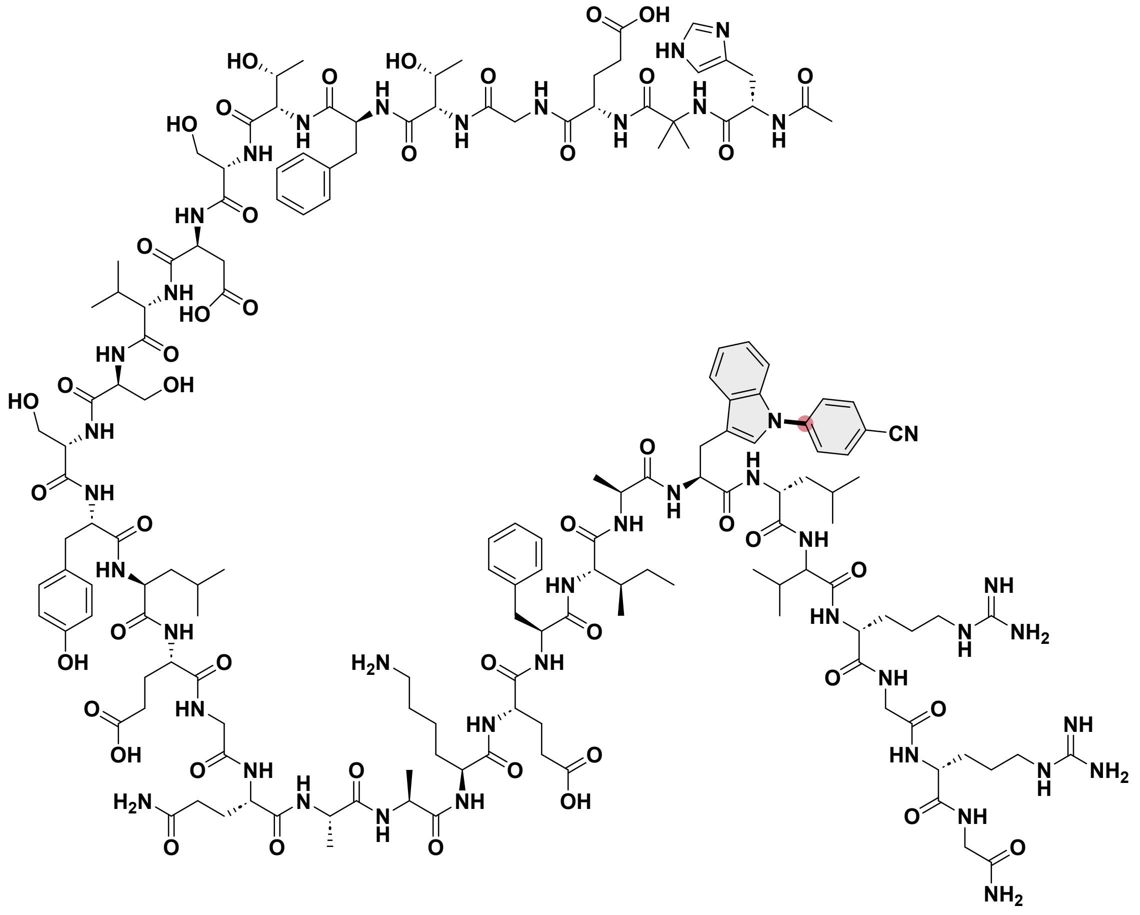

Peptide ***N*****(in)-arylated GLP-1 (7-37)** analog was synthesized according to the general procedure D. Purification by analytical RP-HPLC over several injections and lyophilization afforded the title compound in 16% (25.0 mg) as a white solid. **NMR ^{1}H (600 MHz, DMSO-*d*$_6$)** δ 14.25 (bs, 1H), 12.11 (bs, 2H), 8.91 (s, 1H), 8.30 (d, *J* = 7.7 Hz, 1H), 8.27 (s, 1H), 8.19 (d, *J* = 7.0 Hz, 1H), 8.14 – 8.11 (m, 1H), 8.09 – 8.05 (m, 3H), 8.05 – 8.00 (m, 6H), 7.99 – 7.90 (m, 9H), 7.90 – 7.84 (m, 5H), 7.78 (d, *J* = 8.4 Hz, 3H), 7.74 – 7.70 (m, 2H), 7.68 (bs, 3H), 7.63 – 7.56 (m, 4H), 7.52 – 7.48 (m, 2H), 7.32 (s, 1H), 7.23 (d, *J* = 8.1 Hz, 2H), 7.22 – 7.18 (m, 5H), 7.17 – 7.13 (m, 5H), 7.13 – 7.09 (m, 3H), 7.02 (s, 2H), 6.96 (d, *J* = 8.2 Hz, 3H), 6.77 (s, 1H), 6.58 (d, *J* = 8.1 Hz, 2H), 5.03 (bs, 2H), 4.62 – 4.55 (m, 2H), 4.52 – 4.48 (m, 1H), 4.43 (q, *J* = 7.1 Hz, 1H), 4.35 – 4.28 (m, 3H), 4.27 – 4.24 (m, 2H), 4.22 – 4.14 (m, 13H), 4.11 – 4.07 (m, 2H), 4.03 – 3.99 (m, 1H), 3.95 – 3.92 (m, 1H), 3.87 – 3.82 (m, 1H), 3.76 (td, *J* = 17.2, 5.5 Hz, 3H), 3.71 – 3.63 (m, 4H), 3.61 (d, *J* = 5.7 Hz, 2H), 3.59 – 3.55 (m, 2H), 3.54 – 3.50 (m, 4H), 3.17 – 3.12 (m, 1H), 3.07 – 2.99 (m, 7H), 2.98 – 2.94 (m, 1H), 2.92 – 2.85 (m, 2H), 2.82 – 2.74 (m, 1H), 2.73 – 2.62 (m, 4H), 2.27 – 2.20 (m, 3H), 2.19 – 2.11 (m, 3H), 2.11 –

2.04 (m, 2H), 1.98 – 1.84 (m, 6H), 1.79 (s, 3H), 1.76 – 1.57 (m, 6H), 1.54 – 1.37 (m, 18H), 1.27 (d, J = 6.5 Hz, 7H), 1.20 – 1.18 (m, 15H), 1.15 (d, J = 6.9 Hz, 3H), 0.99 (d, J = 6.3 Hz, 4H), 0.93 (d, J = 6.2 Hz, 3H), 0.84 (d, J = 6.4 Hz, 3H), 0.81 – 0.77 (m, 12H), 0.75 (d, J = 6.4 Hz, 9H), 0.72 (d, J = 6.3 Hz, 6H), 0.69 (d, J = 6.9 Hz, 3H). **NMR ^{13}C (150 MHz, DMSO-d_6)** δ 174.2, 174.0, 172.7, 172.6, 172.4, 172.0, 171.8, 171.6, 171.53, 171.49, 171.3, 171.2, 171.11, 171.07, 171.0, 170.93, 170.86, 170.4, 170.3, 170.24, 170.20, 170.16, 170.0, 169.74, 169.67, 169.1, 168.9, 168.7, 156.8, 155.8, 143.0, 137.7, 137.5, 134.6, 134.1, 133.8, 130.1, 129.8, 129.7, 129.3, 129.2, 128.1, 128.0, 127.6, 126.3, 125.9, 123.4, 123.1, 120.8, 119.4, 118.7, 118.1, 117.0, 116.1, 114.9, 114.4, 110.6, 107.7, 69.8, 66.8, 66.7, 61.9, 61.6, 61.5, 58.0, 57.9, 57.8, 57.7, 57.3, 56.1, 55.6, 55.0, 54.8, 54.0, 53.9, 52.7, 52.39, 52.38, 52.30, 52.0, 51.4, 49.7, 48.8, 48.6, 42.2, 42.0, 41.94, 41.85, 40.6, 40.4, 40.3, 40.1, 38.8, 37.22, 37.18, 36.3, 36.2, 35.7, 35.2, 31.4, 31.3, 31.0, 30.8, 30.4, 30.2, 30.1, 30.0, 29.11, 29.05, 29.01, 28.91, 28.86, 28.8, 28.7, 28.6, 27.7, 27.4, 27.1, 26.7, 26.6, 25.5, 25.2, 25.0, 24.6, 24.2, 24.11, 24.06, 23.2, 23.1, 22.4, 22.3, 22.1, 21.6, 21.4, 19.30, 19.29, 19.2, 18.1, 17.9, 17.8, 17.64, 17.57, 15.2, 14.0, 10.9. **HRMS (ESI)** m/z: $[M+2H]^{2+}$ Calcd. for $C_{161}H_{236}N_{44}O_{47}$ 1769.8788; Found: 1769.8794, $[M+3H]^{3+}$ Calcd. for $C_{161}H_{236}N_{44}O_{47}$ 1180.2549; Found: 1180.2560, and $[M+4H]^{4+}$ Calcd. for $C_{161}H_{236}N_{44}O_{47}$ 885.4430; Found: 885.4448.

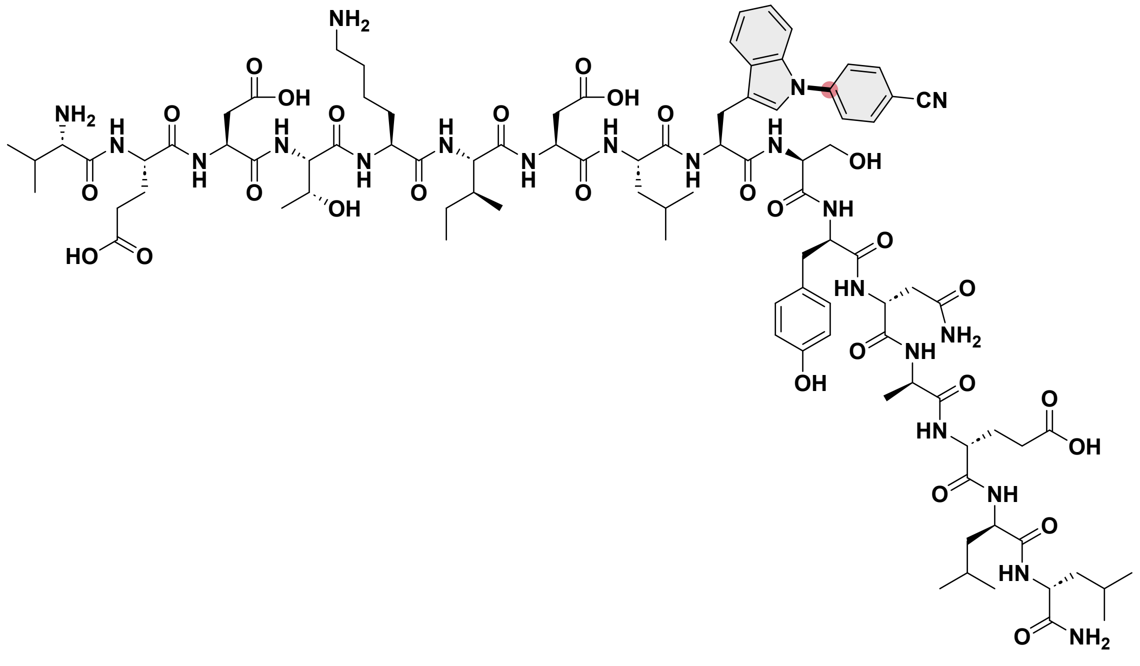

Peptide ***N***$_{\textbf{(in)}}$**-arylated Flufirvitide-3** analog was synthesized according to the general procedure D. Purification by analytical RP-HPLC over several injections and lyophilization afforded the title compound in 15% (14.0 mg) as a white solid. **NMR ^{1}H (600 MHz, DMSO-*d*$_6$)** δ 12.23 (bs, 3H), 9.11 (s, 1H), 8.51 (d, *J* = 8.1 Hz, 1H), 8.39 (d, *J* = 7.3 Hz, 1H), 8.27 (d, *J* = 7.2 Hz, 1H), 8.23 (d, *J* = 7.7 Hz, 1H), 8.18 – 8.15 (m, 1H), 8.05 (s, 2H), 8.00 (d, *J* = 7.3 Hz, 2H), 7.97 (d, *J* = 8.5 Hz, 2H), 7.88 (d, *J* = 7.5 Hz, 1H), 7.84 – 7.81 (m, 2H), 7.77 (d, *J* = 8.6 Hz, 2H), 7.74 (d, *J* = 8.2 Hz, 1H), 7.69 – 7.65 (m, 3H), 7.61 (d, *J* = 8.4 Hz, 1H), 7.57 (d, *J* = 7.7 Hz, 1H), 7.53 (d, *J* = 8.2 Hz, 1H), 7.48 (s, 1H), 7.42 (s, 1H), 7.19 (t, *J* = 7.5 Hz, 1H), 7.09 (t, *J* = 7.5 Hz, 1H), 7.01 (s, 1H), 6.97 (s, 1H), 6.93 (d, *J* = 8.6 Hz, 3H), 6.55 (d, *J* = 8.4 Hz, 2H), 4.96 – 4.84 (m, 1H), 4.64 (dt, *J* = 7.5, 7.1 Hz, 1H), 4.58 (q, *J* = 7.1 Hz, 1H), 4.53 – 4.46 (m, 2H), 4.40 (q, *J* = 7.2 Hz, 1H), 4.36 (td, *J* = 8.1, 5.5 Hz, 1H), 4.30 – 4.23 (m, 2H), 4.19 – 4.13 (m, 4H), 4.12 – 4.07 (m, 3H), 3.96 – 3.91 (m, 1H), 3.62 – 3.58 (m, 1H), 3.57 – 3.51 (m, 1H), 3.50 – 3.47 (m, 1H), 3.47 (s, 2H), 2.73 – 2.68 (m, 3H), 2.67 – 2.61 (m, 3H), 2.60 – 2.53 (m, 2H), 2.49 – 2.43 (m, 2H), 2.43 – 2.37 (m, 1H), 2.31 – 2.17 (m, 4H), 2.02 – 1.96 (m, 1H), 1.94 – 1.82 (m, 2H), 1.81 – 1.69 (m, 2H), 1.67 – 1.59 (m, 2H), 1.56 – 1.43 (m, 8H), 1.42 – 1.36 (m, 2H), 1.34 – 1.25 (m, 6H), 1.21 – 1.19 (m, 5H), 1.01 – 0.95 (m, 4H), 0.88 (d, *J* = 6.8 Hz, 6H), 0.83 (d, *J* = 6.5 Hz, 3H), 0.82 (d, *J* = 6.7 Hz, 3H), 0.77 – 0.76 (m, 6H), 0.75 – 0.73 (m, 6H), 0.72 – 0.69 (m, 6H). **NMR ^{13}C (150 MHz, DMSO-*d*$_6$)** δ 174.1, 174.0, 173.9, 172.9, 171.92, 171.85, 171.8, 171.7, 171.6, 171.5, 171.3, 171.2, 171.1, 170.8, 170.7, 170.53, 170.46, 169.9, 169.6, 167.7, 155.9, 143.0, 134.5, 134.1, 130.2, 129.8, 129.7, 127.3, 126.0, 123.3, 123.1, 120.8, 119.7, 118.8, 114.9, 114.5, 110.5, 107.6, 69.8, 66.5, 61.7, 58.0, 57.3, 56.6, 55.1, 54.3, 52.8, 52.34, 52.29, 51.7, 51.39, 51.36, 51.1, 49.7, 49.5, 49.3, 40.7, 40.4, 40.1, 38.8, 36.94, 36.90, 36.6, 35.8, 35.7, 31.2, 30.2, 30.0, 29.9, 29.1, 28.9, 28.7, 27.8, 26.6, 26.5, 24.18, 24.17, 24.0, 23.1, 22.2, 21.41, 21.35, 19.2, 18.4, 17.8, 17.5, 15.1, 11.0. **HRMS (ESI)** m/z: $[M+H]^+$ Calcd. for $C_{94}H_{137}N_{21}O_{28}$ 2009.0015; Found: 2009.0015, $[M+2H]^{2+}$ Calcd. for $C_{161}H_{236}N_{44}O_{47}$ 1005.0044; Found: 1005.0058, and $[M+3H]^{3+}$ Calcd. for $C_{161}H_{236}N_{44}O_{47}$ 670.3387; Found: 670.3399.

10. NMR Spectra.

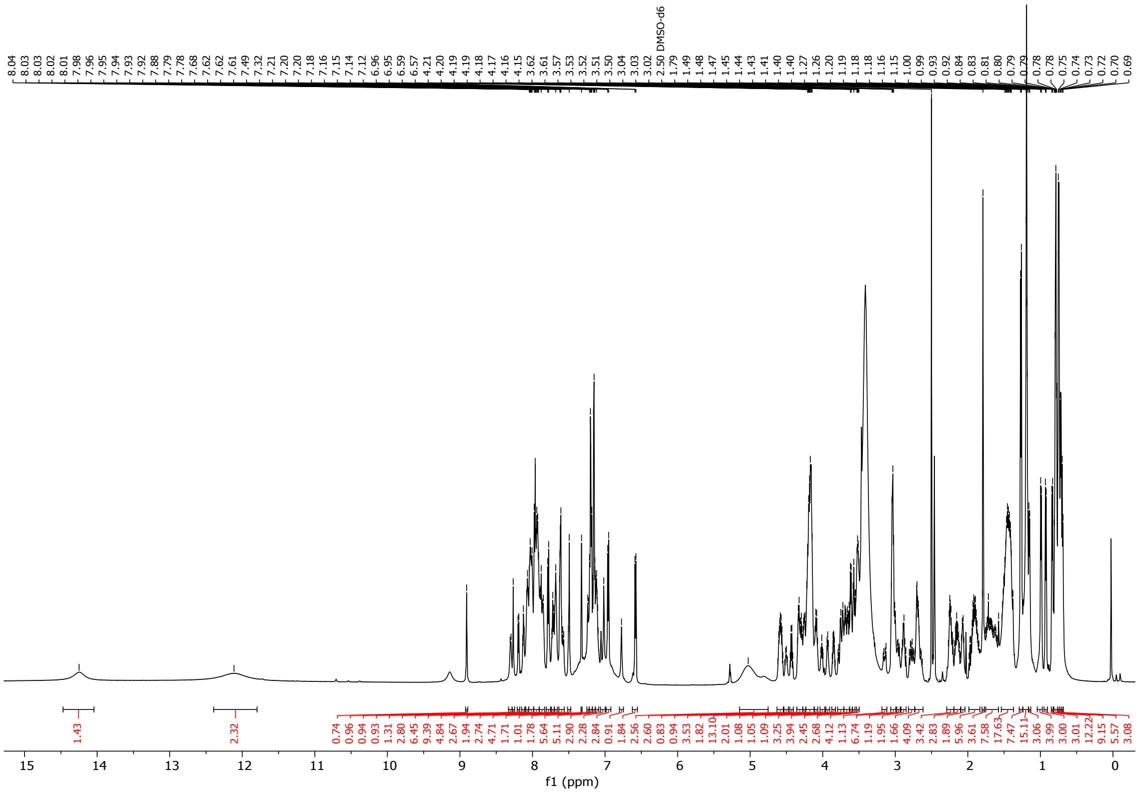

Figure 31. ^{1}H NMR of ***N*$_{(in)}$-arylated GLP-1 (7-37)** analog (600 MHz, DMSO-d_6).

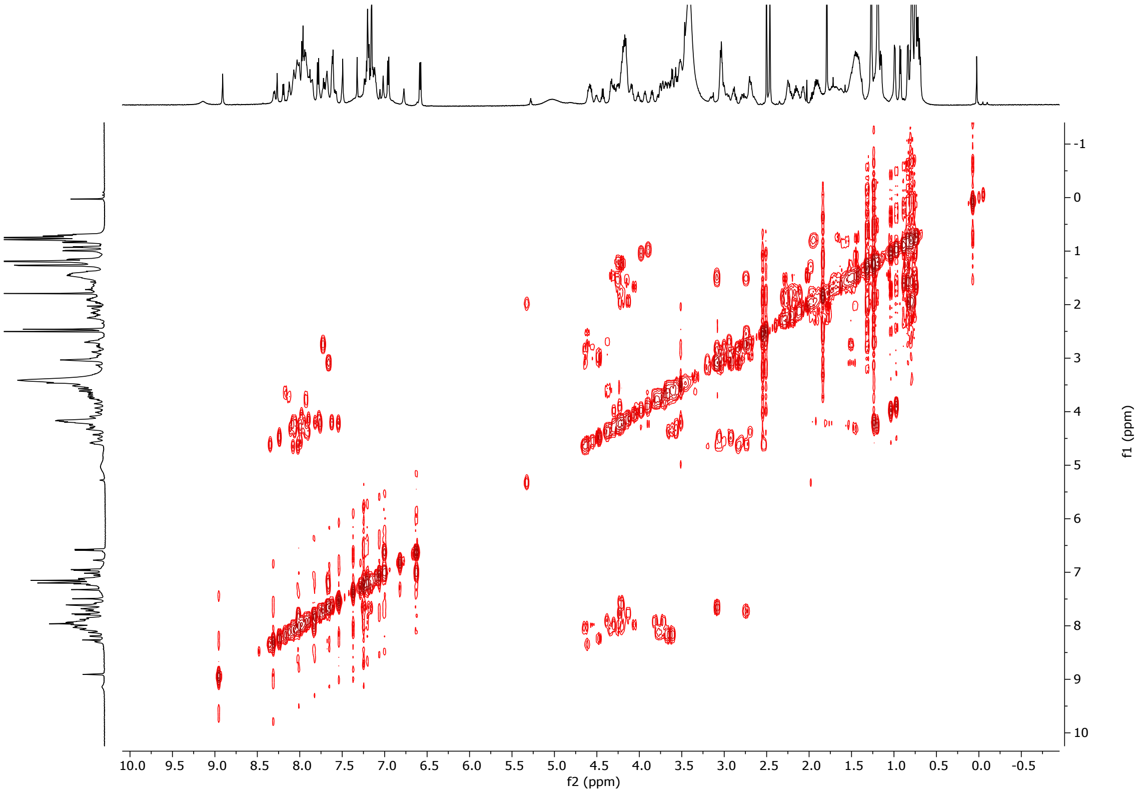

Figure 32. COSY NMR of ***N*$_{(in)}$-arylated GLP-1 (7-37)** analog (600 MHz, DMSO-d_6).

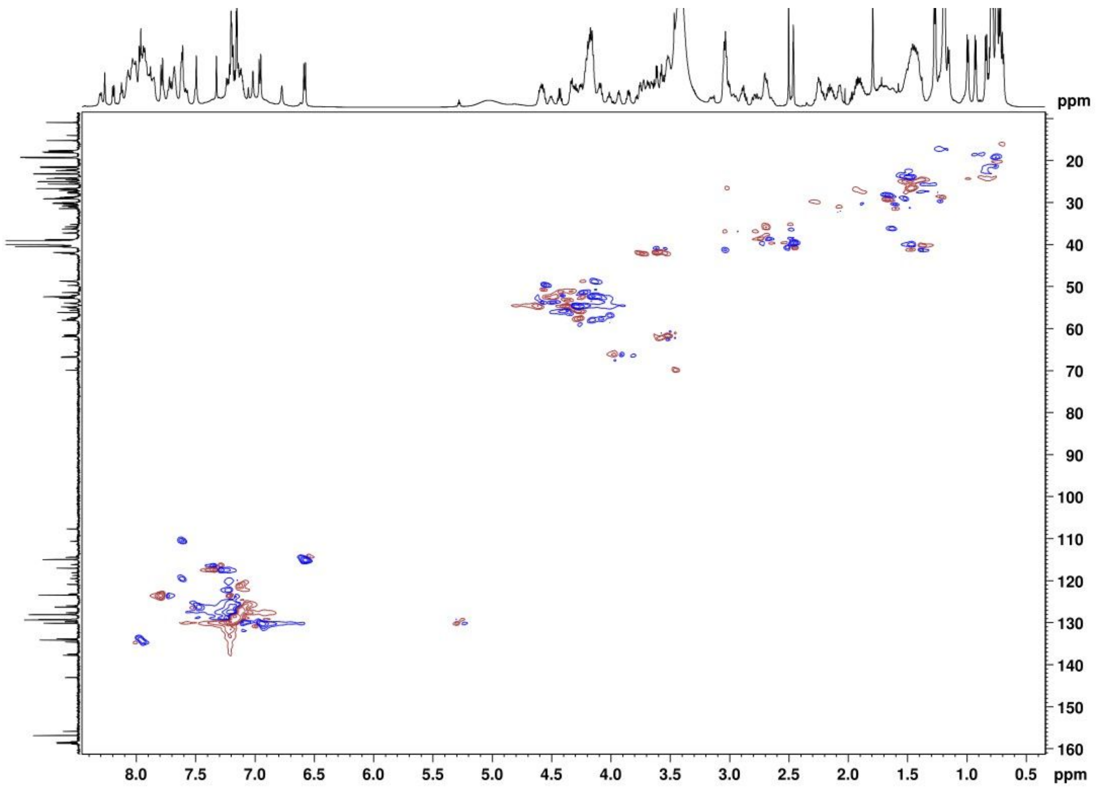

Figure 33. ^{1}H-^{13}C HSQC NMR of ***N*****$_{(in)}$-arylated GLP-1 (7-37)** analog (600 MHz, DMSO-d_6).

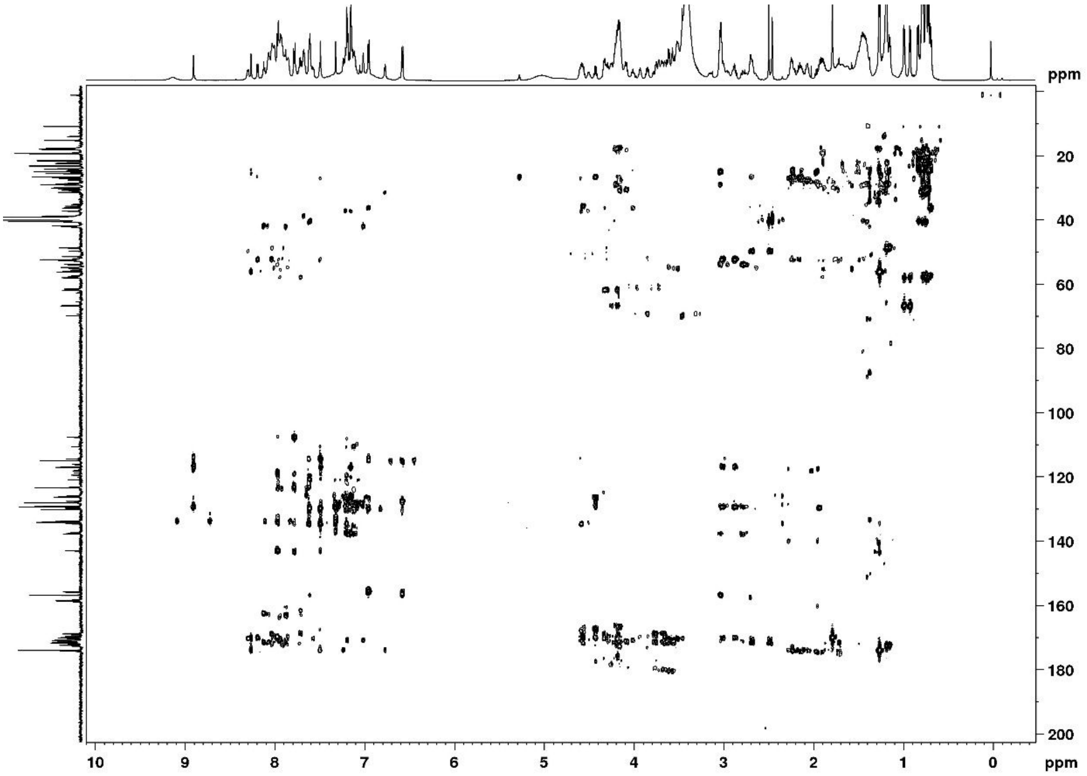

Figure 34. ^{1}H-^{13}C HMBC NMR of ***N*****$_{(in)}$-arylated GLP-1 (7-37)** analog (600 MHz, DMSO-d_6).

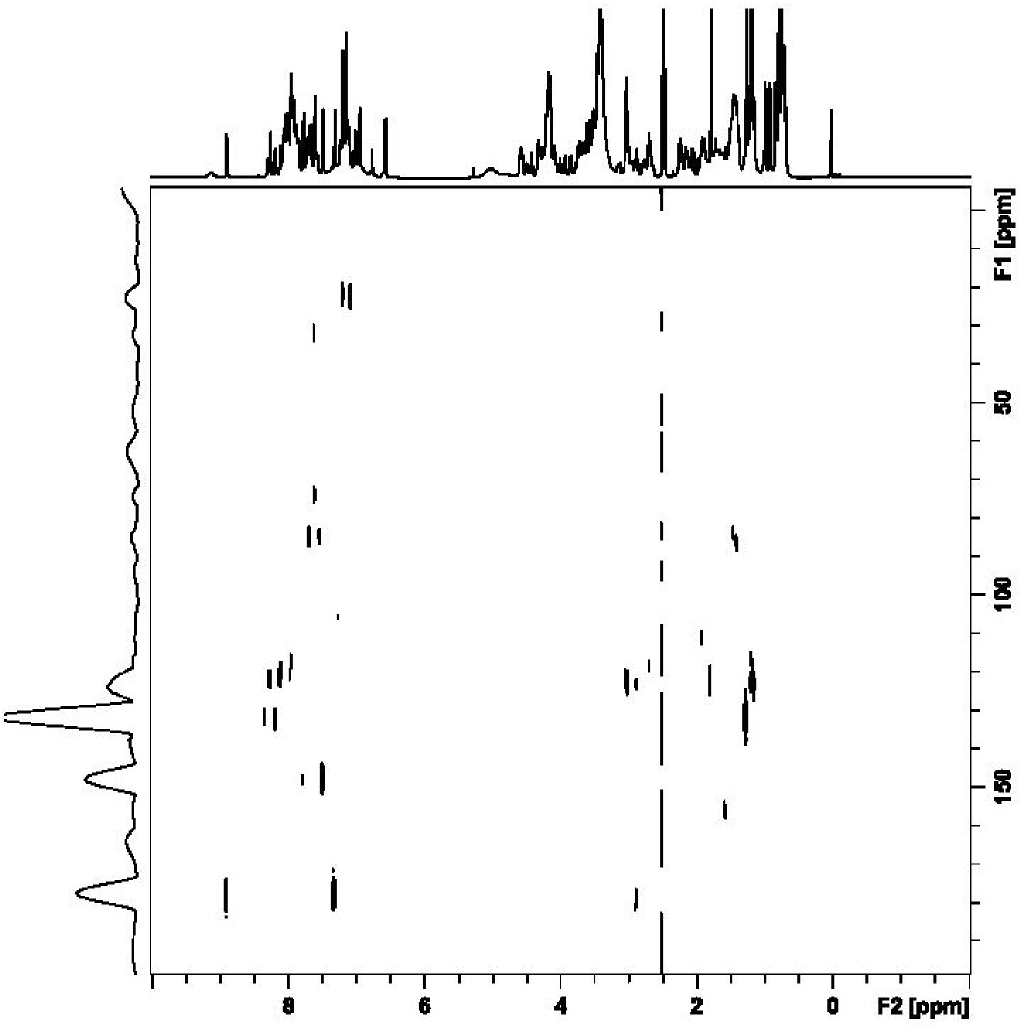

Figure 35. ^1H-^{15}N HMBC NMR of ***N*$_{(in)}$-arylated GLP-1 (7-37)** analog (600 MHz, DMSO-d_6).

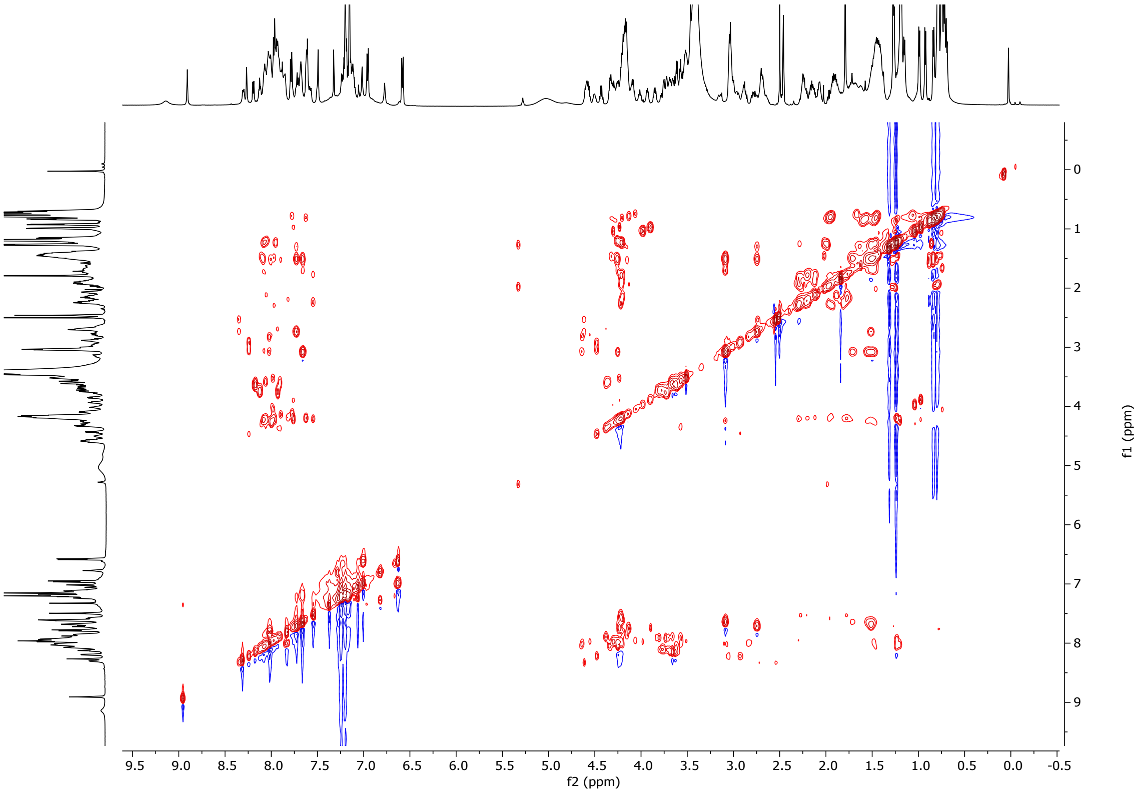

Figure 36. TOCSY NMR of ***N*$_{(in)}$-arylated GLP-1 (7-37)** analog (600 MHz, DMSO-d_6).

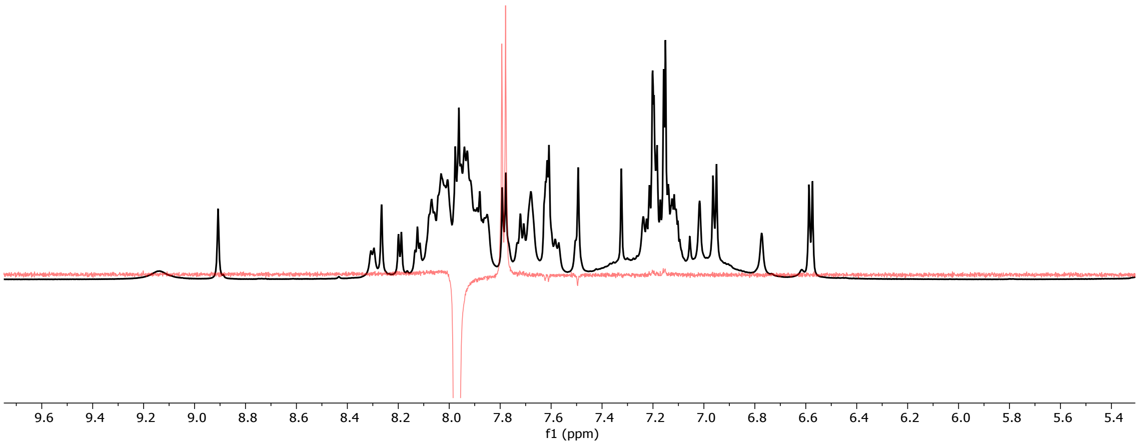

Figure 37. Overlapped ^{1}H- (grey) and selective 1D-ROESY (red) (irradiation at the δ 7.97 (d, *J* = 8.5 Hz, 2H) signal) NMRs of ***N*$_{(in)}$-arylated GLP-1 (7-37)** analog (600 MHz, DMSO-d_6).

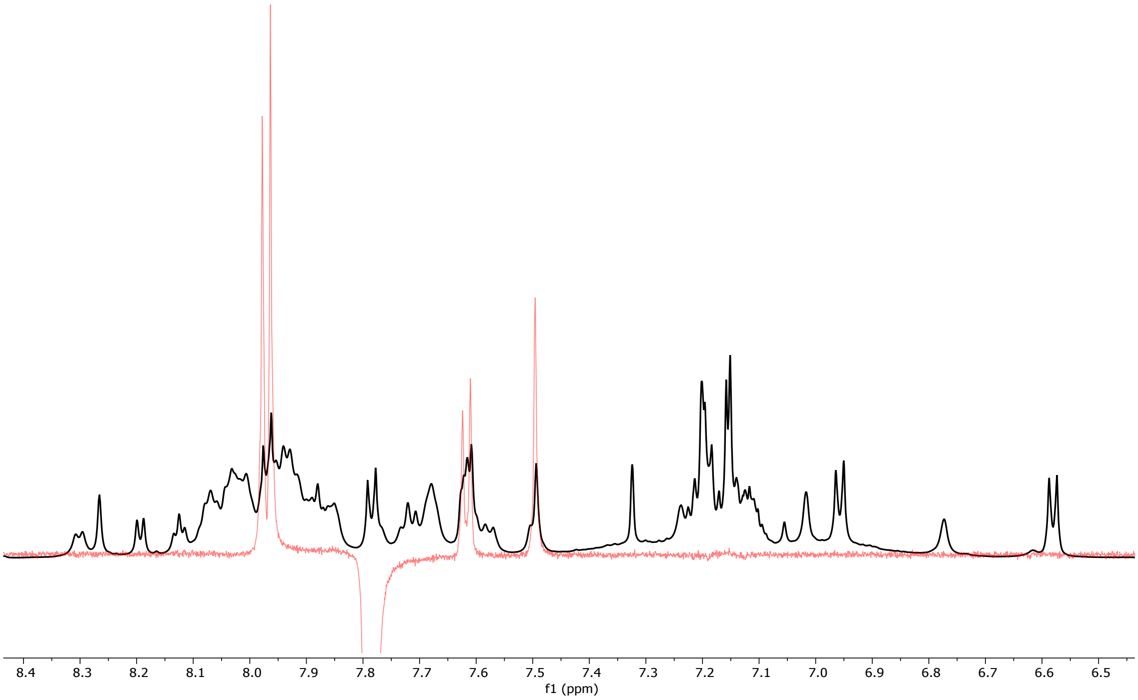

Figure 38. Overlapped ^{1}H- (grey) and selective 1D-ROESY (red) (irradiation at the δ 7.78 (d, J = 8.4 Hz, 2H) signal) NMRs of ***N*$_{(in)}$-arylated GLP-1 (7-37)** analog (600 MHz, DMSO-d_6).

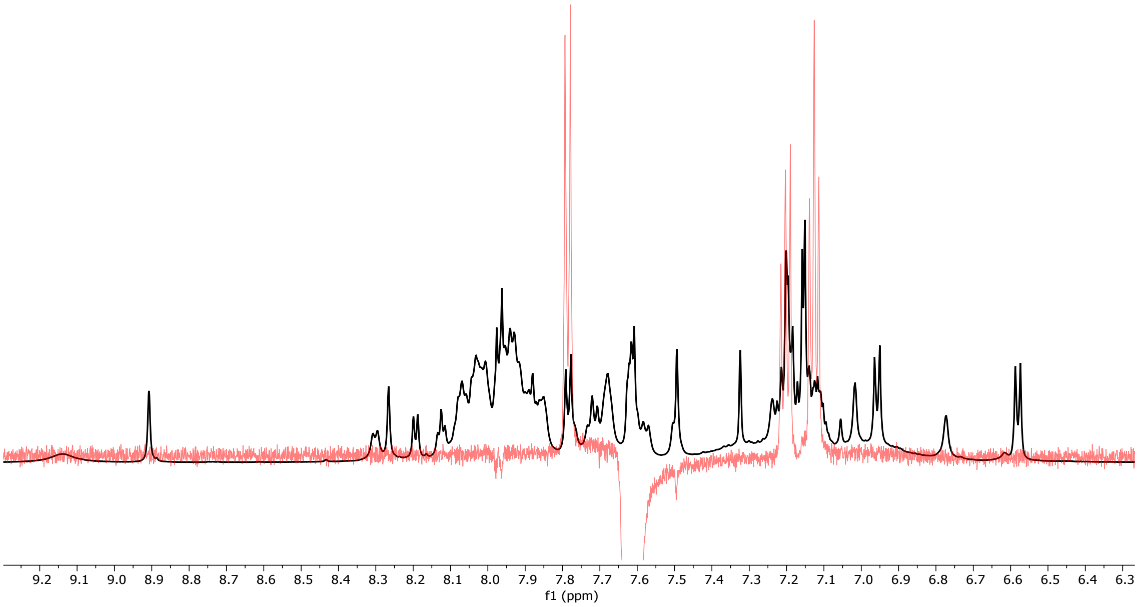

Figure 39. Overlapped ^{1}H- (grey) and selective 1D-ROESY (red) (irradiation at the δ 7.63 – 7.61 ppm (m, 2H) signal) NMRs of ***N*$_{(in)}$-arylated GLP-1 (7-37)** analog (600 MHz, DMSO-d_6).

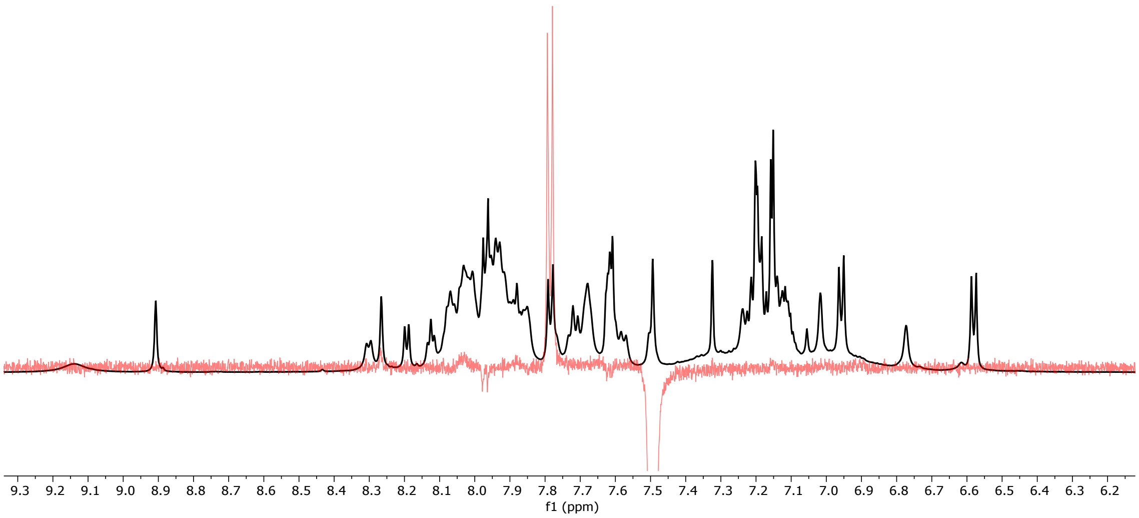

Figure 40. Overlapped ^{1}H- (grey) and selective 1D-ROESY (red) (irradiation at the δ 7.49 ppm (s, 1H) signal) NMRs of ***N*$_{(in)}$-arylated GLP-1 (7-37)** analog (600 MHz, DMSO-d_6).

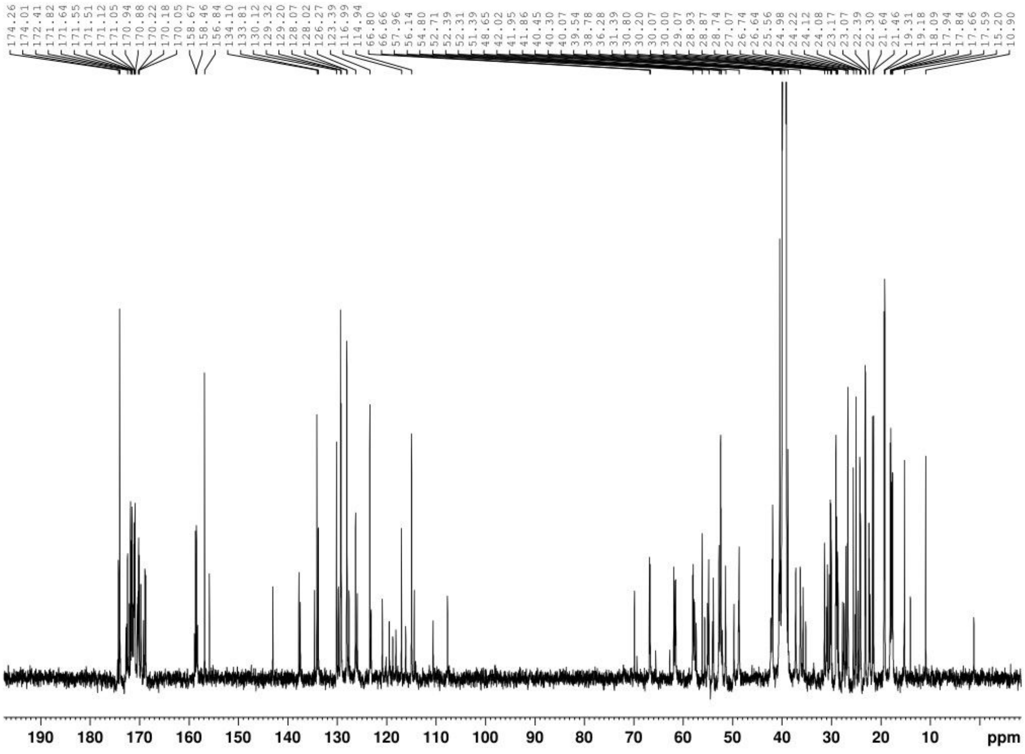

Figure 41. ^{13}C NMR of ***N*$_{(in)}$-arylated GLP-1 (7-37)** analog (150 MHz, DMSO-d_6).

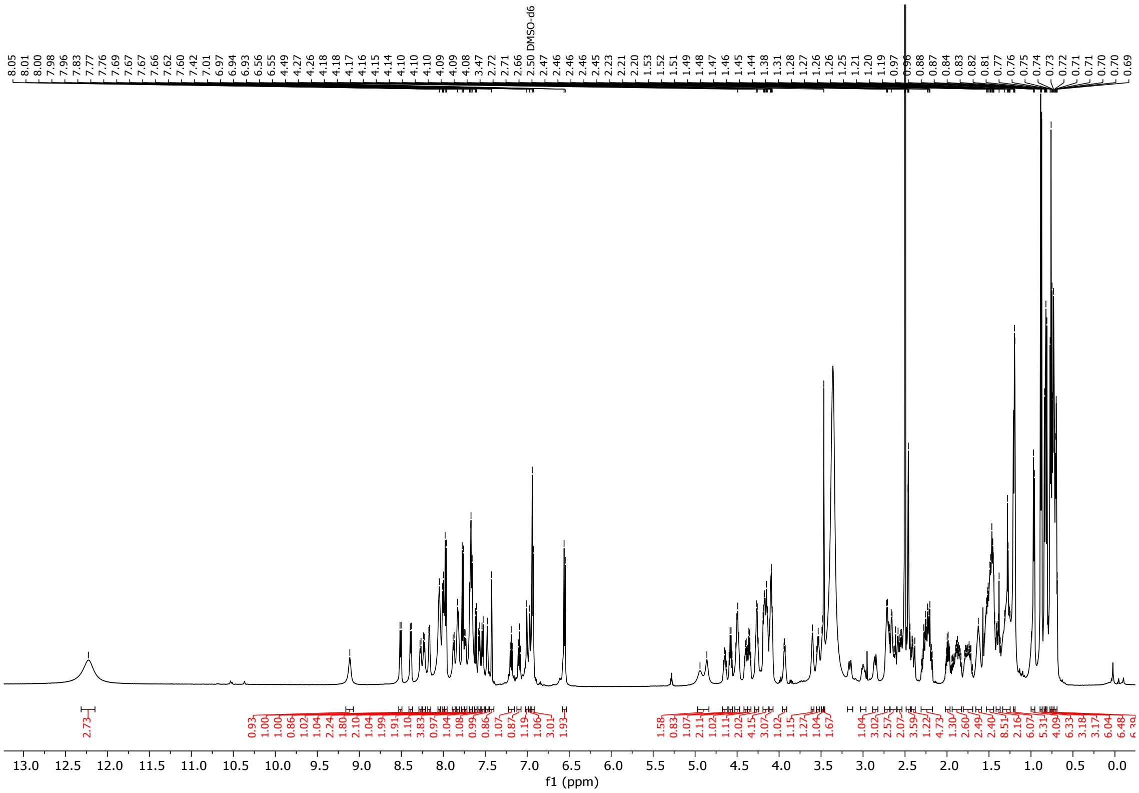

Figure 42. ^{1}H NMR of ***N*$_{(in)}$-arylated Flufirvitide-3** analog (600 MHz, DMSO-d_6).

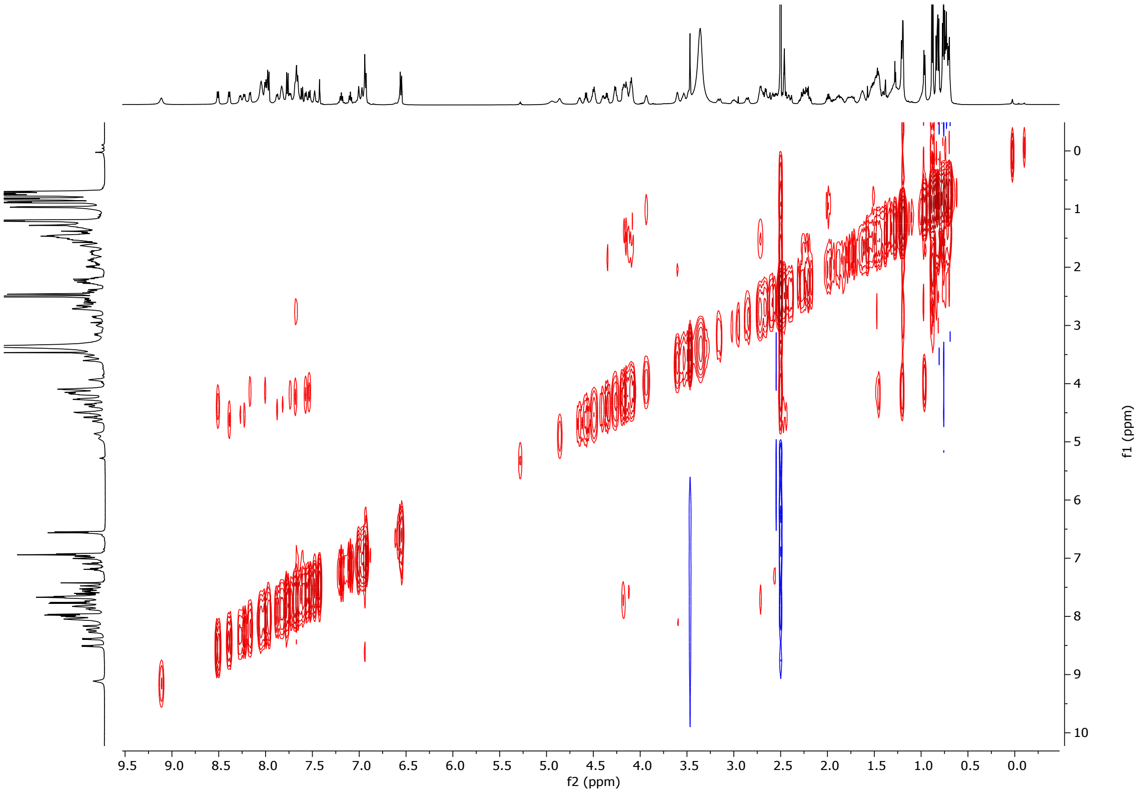

Figure 43. COSY NMR of ***N*$_{(in)}$-arylated Flufirvitide-3** analog (600 MHz, DMSO-d_6).

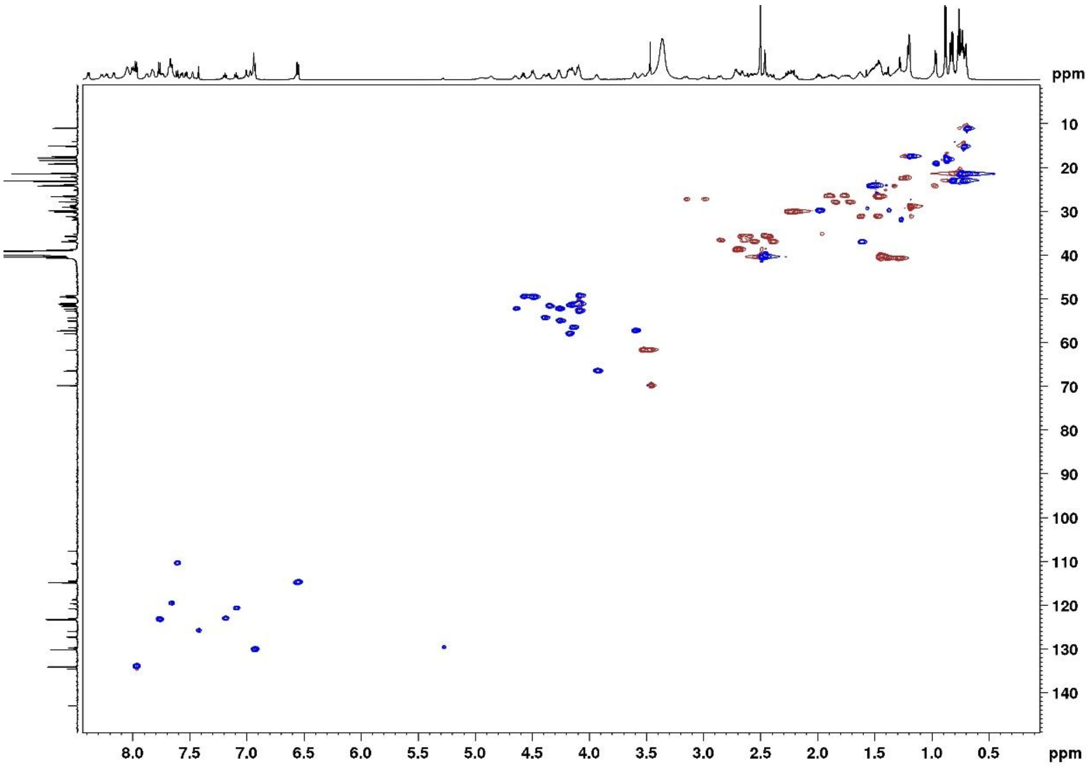

Figure 44. ^{1}H-^{13}C HSQC NMR of ***N*$_{(in)}$-arylated Flufirvitide-3** analog (600 MHz, DMSO-d_6).

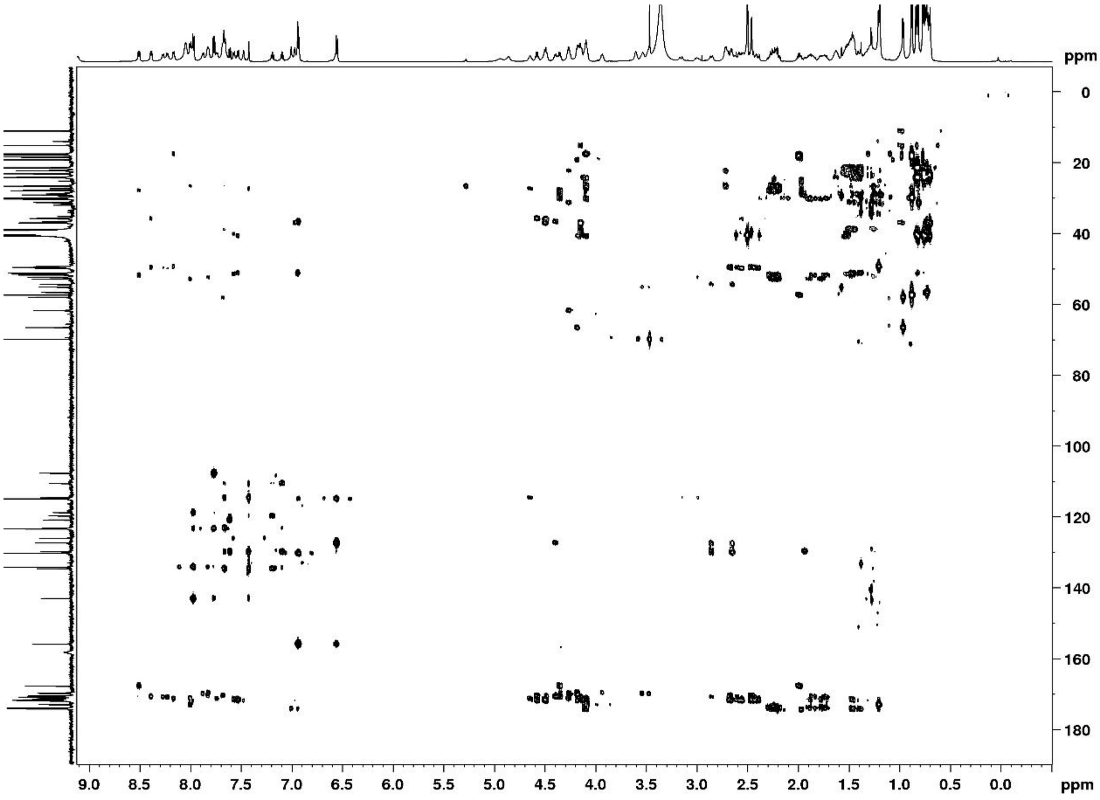

Figure 45. ^{1}H-^{13}C HMBC NMR of ***N*$_{(in)}$-arylated Flufirvitide-3** analog (600 MHz, DMSO-d_6).

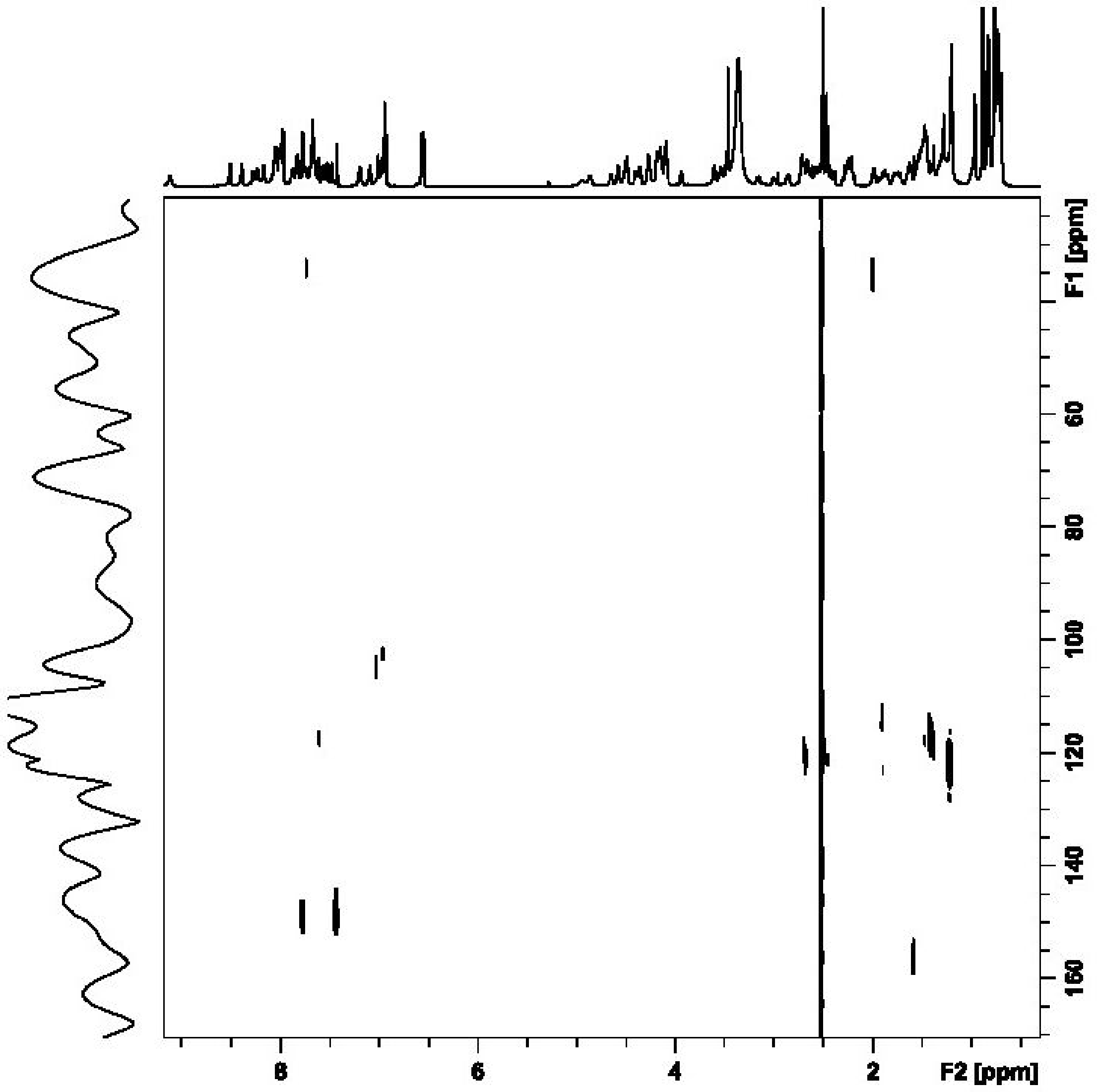

Figure 46. ^1H-^{15}N HMBC NMR of ***N*****(in)-arylated Flufirvitide-3** analog (600 MHz, DMSO-d_6).

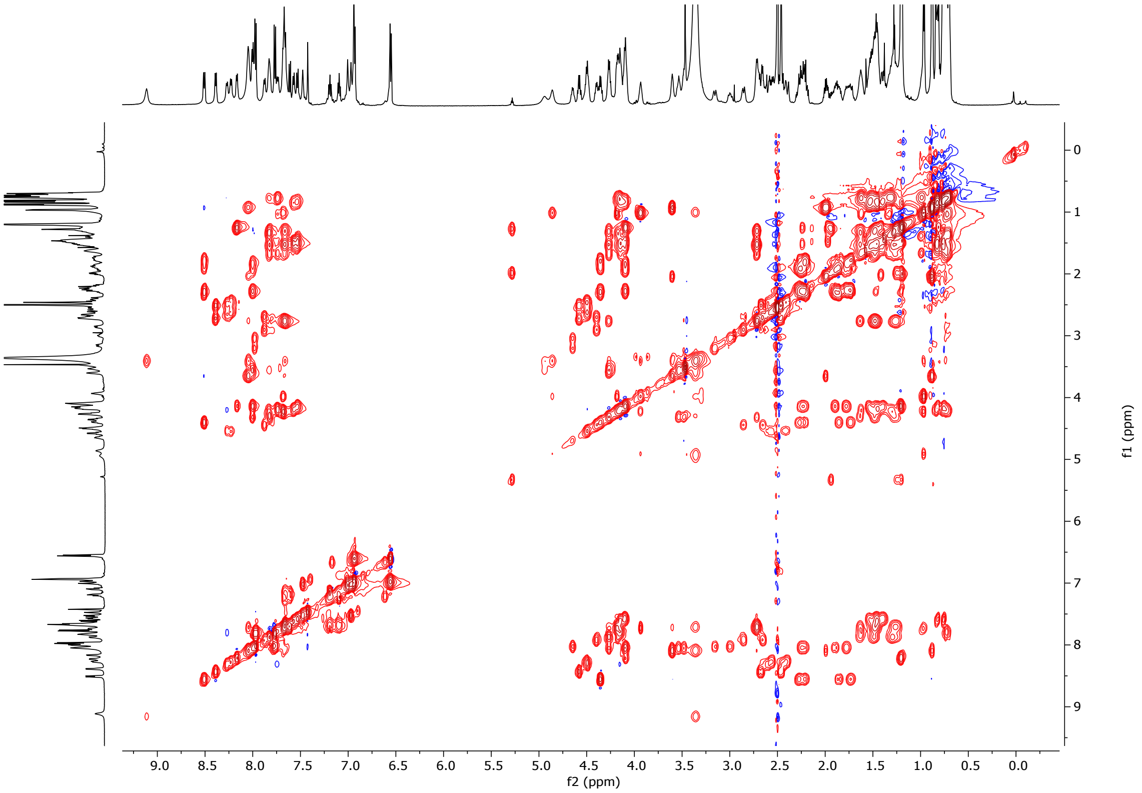

Figure 47. TOCSY NMR of ***N*_(in)-arylated Flufirvitide-3** analog (600 MHz, DMSO-d_6).

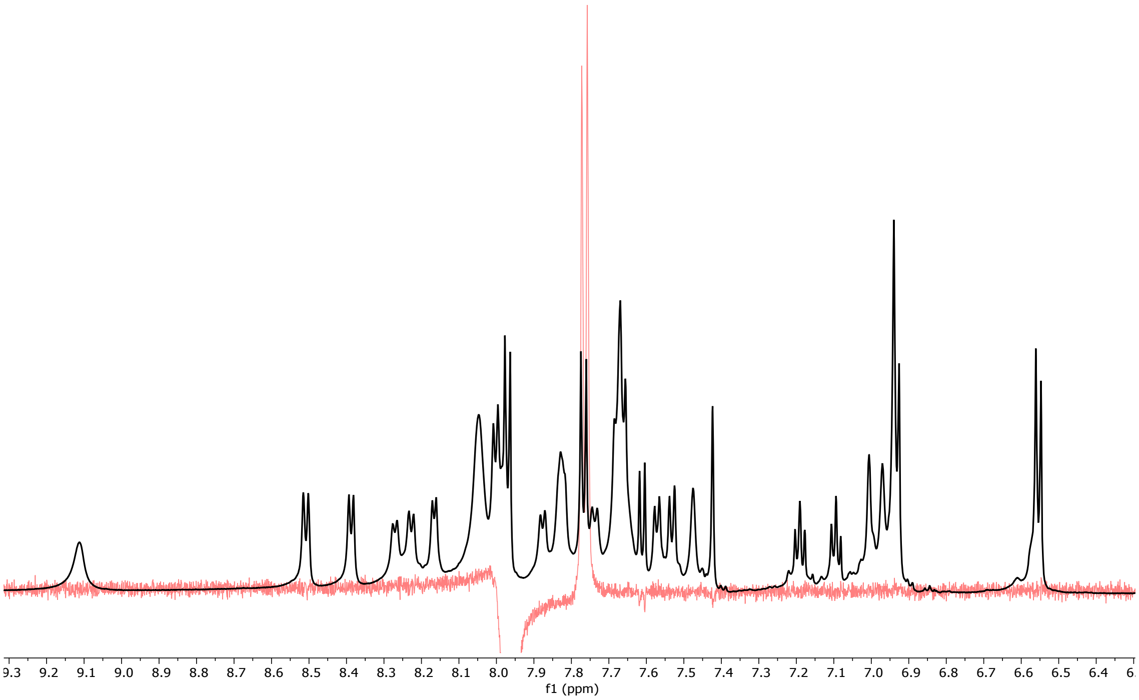

Figure 48. Overlapped ^{1}H- (grey) and selective 1D-ROESY (red) (irradiation at the δ 7.97 (d, *J* = 8.5 Hz, 2H) signal) NMRs of ***N*$_{(in)}$-arylated Flufirvitide-3** analog (600 MHz, DMSO-d_6).

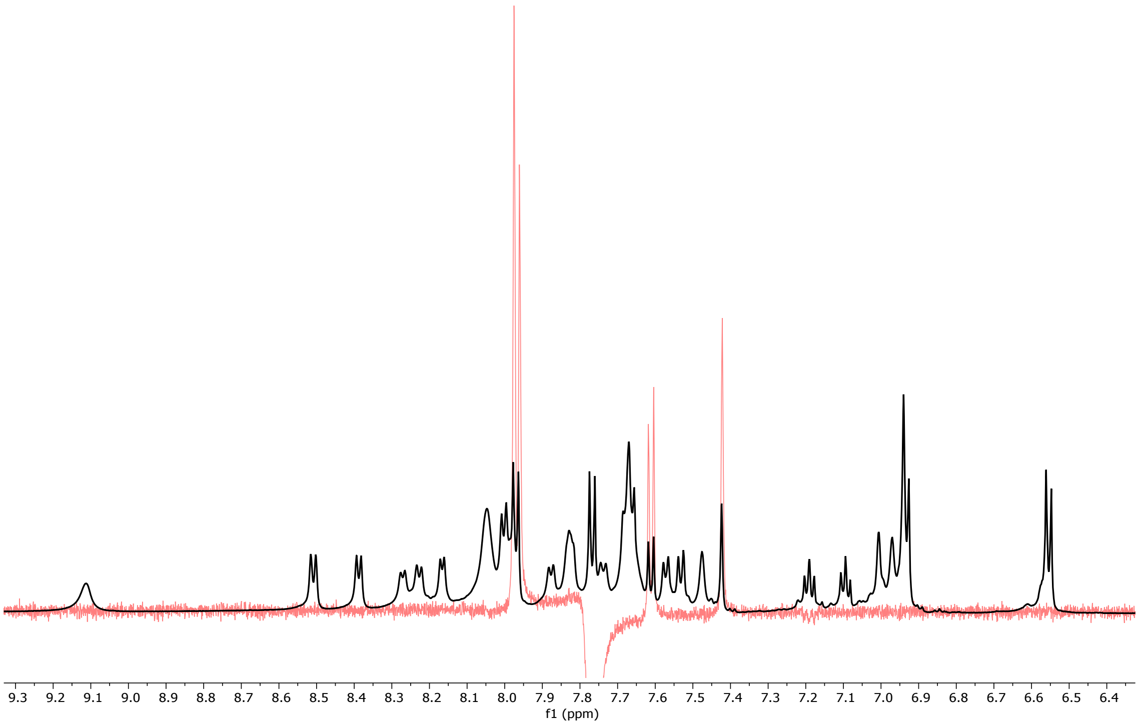

Figure 49. Overlapped ^{1}H- (grey) and selective 1D-ROESY (red) (irradiation at the δ 7.77 (d, J = 8.6 Hz, 2H) signal) NMRs of ***N*$_{(in)}$-arylated Flufirvitide-3** analog (600 MHz, DMSO-d_6).

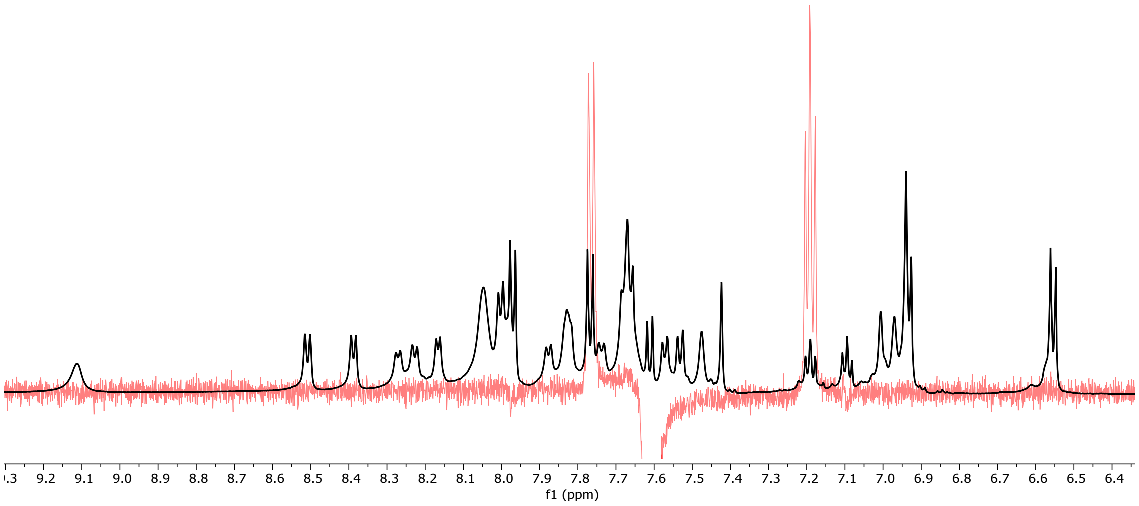

Figure 50. Overlapped ^{1}H- (grey) and selective 1D-ROESY (red) (irradiation at the δ 7.61 ppm (d, J = 8.4 Hz, 2H) signal) NMRs of ***N*$_{(in)}$-arylated Flufirvitide-3** analog (600 MHz, DMSO-d_6).

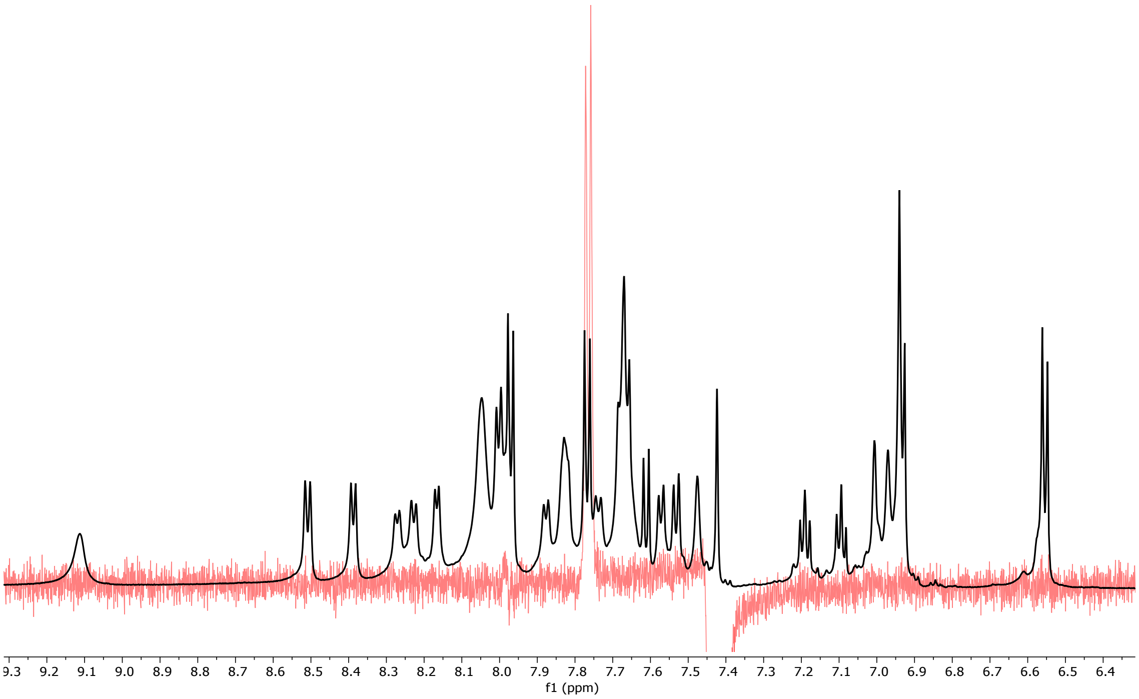

Figure 51. Overlapped ^{1}H- (grey) and selective 1D-ROESY (red) (irradiation at the δ 7.42 ppm (s, 1H) signal) NMRs of ***N*$_{(in)}$-arylated Flufirvitide-3** analog (600 MHz, DMSO-d_6).

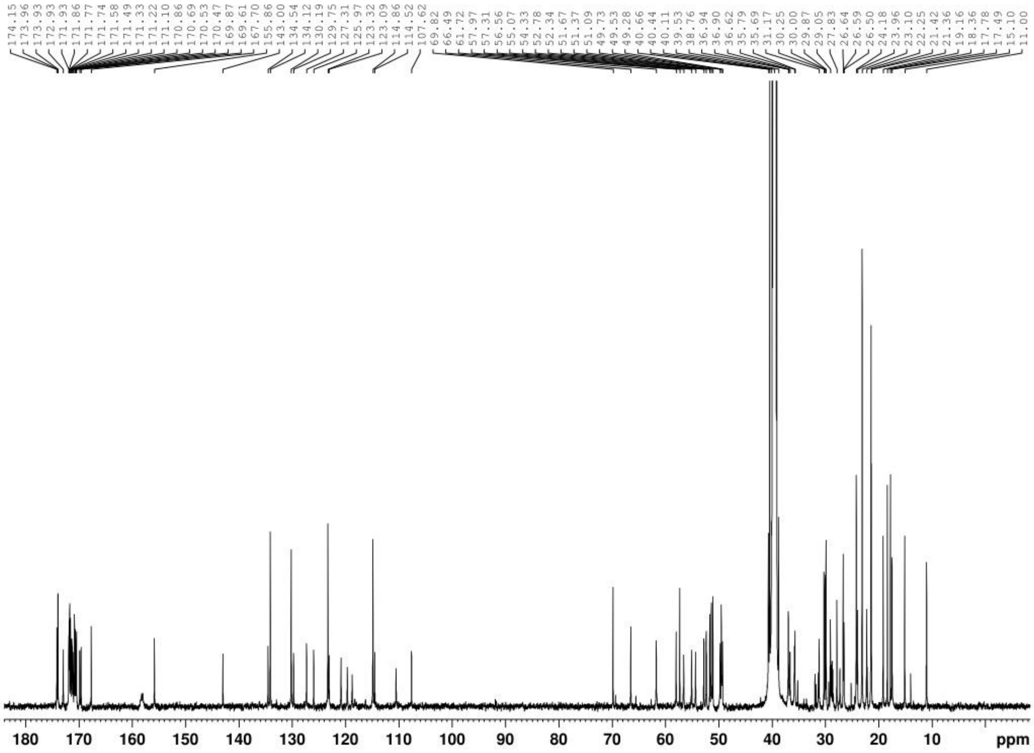

Figure 52. ^{13}C NMR of ***N*$_{(in)}$-arylated Flufirvitide-3** analog (150 MHz, DMSO-d_6).

11. References

(1) Armarego, W. L. F.; Chai, C. L. L. *Purification of Laboratory Chemicals*, Fifth Edit.; Butterworth-Heinemann, 2003.

(2) Fulmer, G. R.; Miller, A. J. M.; Sherden, N. H.; Gottlieb, H. E.; Nudelman, A.; Stoltz, B. M.; Bercaw, J. E.; Goldberg, K. I. NMR Chemical Shifts of Trace Impurities: Common Laboratory Solvents, Organics, and Gases in Deuterated Solvents Relevant to the Organometallic Chemist. *Organometallics* **2010**, *29* (9), 2176–2179.

(3) Jensen, K.J., Shelton, P.T. and Pedersen, S. L. (Ed). *Peptide Synthesis and Applications*; Jensen, K. J., Tofteng Shelton, P., Pedersen, S. L., Eds.; Methods in Molecular Biology; Humana Press: Totowa, NJ, 2013; Vol. 1047.

(4) Al Musaimi, O.; Basso, A.; de la Torre, B. G.; Albericio, F. Calculating Resin Functionalization in Solid-Phase Peptide Synthesis Using a Standardized Method Based on Fmoc Determination. *ACS Comb Sci* **2019**, *21* (11), 717–721.

12. Reagents

• 4-[(2,4-Dimethoxyphenyl)(amino)methyl]phenoxyacetic acid, polymer-bound to ChemMatrix® resin (H-Rink amide ChemMatrix® resin; Sigma-Aldrich, cat. no. 727768)
• *N*-(9-Fluorenylmethoxycarbonyl)-L-glycine (Fmoc-Gly-OH; Sigma-Aldrich, cat. no. 47627)
• *N*-(9-Fluorenylmethoxycarbonyl)-L-alanine (Fmoc-Ala-OH; Sigma-Aldrich, cat. no. 531480)
• *N*-(9-Fluorenylmethoxycarbonyl)-L-valine (Fmoc-Val-OH; Sigma-Aldrich, cat. no. 47638)
• *N*-(9-Fluorenylmethoxycarbonyl)-L-leucine (Fmoc-Leu-OH; Sigma-Aldrich, cat. no. 47633)
• *N*-(9-Fluorenylmethoxycarbonyl)-L-isoleucine (Fmoc-Ile-OH; Sigma-Aldrich, cat. no. 47628)
• *N*-(9-Fluorenylmethoxycarbonyl)-*O*-*tert*-butyl-L-serine (Fmoc-Ser(*t*Bu)-OH; Sigma-Aldrich, cat. no. 47619)
• *N*-(9-Fluorenylmethoxycarbonyl)-*O*-*tert*-butyl-L-threonine (Fmoc-Thr(*t*Bu)-OH; Sigma-Aldrich, cat. no. 47622)
• *N*-(9-Fluorenylmethoxycarbonyl)-L-phenylalanine (Fmoc-Phe-OH; Sigma-Aldrich, cat. no. 338338)
• *N*-(9-Fluorenylmethoxycarbonyl)-*O*-*tert*-butyl-L-tyrosine, (Fmoc-Tyr(*t*Bu)-OH; Sigma-Aldrich, cat. no. 47623)
• N_{α}-(9-Fluorenylmethoxycarbonyl)-$N_{(im)}$-trityl-L-histidine (Fmoc-His(Trt)-OH; Sigma-Aldrich, cat. no. 47639)
• *N*-(9-Fluorenylmethoxycarbonyl)-L-tryptophan (Fmoc-Trp-OH; AmBeed, Inc., cat. no. A203213)
• N_{α}-(9-Fluorenylmethoxycarbonyl)-N_{ε}-(*tert*-butyloxycarbonyl)-L-lysine (Fmoc-Lys(Boc)-OH; Sigma-Aldrich, cat. no. 47624)
• N_{α}-(9-Fluorenylmethoxycarbonyl)-N_{ω}-(2,2,4,6,7-pentamethyldihydrobenzofuran-5-sulfonyl)-L-arginine (Fmoc-Arg(Pbf)-OH; Sigma-Aldrich, cat. no. 47349)
• *N*-(9-Fluorenylmethoxycarbonyl)-L-aspartic acid 4-*tert*-butyl ester (Fmoc-

Asp(O*t*Bu)-OH; Sigma-Aldrich, cat. no. 47618)
• *N*-(9-Fluorenylmethoxycarbonyl)-L-glutamic acid 5-*tert*-butyl ester (Fmoc-Glu(O*t*Bu)-OH; Sigma-Aldrich, cat. no. 47625)
• N_α-(9-Fluorenylmethoxycarbonyl)-N_γ-trityl-L-asparagine (Fmoc-Asn(Trt)-OH; Nfinitu Tech LLC, cat. no. 857898)
• N_α-(9-Fluorenylmethoxycarbonyl)-N_δ-trityl-L-glutamine (Fmoc-Gln(Trt)-OH; Sigma-Aldrich, cat. no. 47674)
• 1-Hydroxybenzotriazole hydrate (HOBt; Sigma-Aldrich, cat. no. 54802)
• 1,2,3-Indantrione monohydrate (Ninhydrin; Sigma-Aldrich, cat. no. 151173)
• *N,N'*-Diisopropylcarbodiimide (DIC; Oakwood Chemical, cat. no. M02889)
• *N,N*-Diisopropylethylamine (DIPEA; Sigma-Aldrich, cat. no. D125806)
• Ethanol (Merck KGaA, cat. no. 1.11727.4000)
• Methanol (Biograde, cod. PA1368-G1L)
• *N,N*-Dimethylformamide (DMF; Biograde, cod. HS1693-GL4L)
• Diethyl ether (ÊXODO científica, cod. EE04837RA)
• Dichloromethane (DCM; ÊXODO científica, cod. D06960RA)
• Acetic anhydride (Sigma-Aldrich, cat. no. 242845)
• Nickel(II) chloride ethylene glycol dimethyl ether complex ($NiCl_2$-Glyme; Sigma-Aldrich, cat. no. 696668)
• 4,4′-Di-*tert*-butyl-2,2′-dipyridyl (dtbbpy; Sigma-Aldrich, cat. no. 515477)
• Potassium phosphate dibasic (K_2HPO_4; Sigma-Aldrich, cat. no. P3786)
• 4-Bromobenzonitrile (TCI Chemicals, cat. no. B1426)
• Triisopropylsilane (TIPS; AmBeed, cat. no. A187865)
• Dimethyl sulfoxide (DMSO; Aldrich, cat. no. M8, 180-2)
• Dimethyl sulfoxide-d_6 (DMSO-d_6 (0.75 mL ampules); Sigma-Aldrich, cat. no. 296147)
• *n*-Butanol (ÊXODO científica, cod. AB08439RA)
• Acetonitrile (ACN; Sigma-Aldrich, cat. no. 34851)
• Acetonitrile (LC-MS grade; Merck KGaA, cat. no. 1.00029.2500)
• Water (H_2O; deionized water, > 15 MΩ/cm resistance, filtered through a 0.2 μm disc filter, Millipak® Express 40, cat. no. MPGP04001)
• Water (LC-MS grade; Merck KGaA, cat. no. 1.15333.4000)
• Trifluoroacetic acid (TFA; Sigma-Aldrich, cat. no. 302031)
• Formic acid (LC-MS grade; ÊXODO científica, cod. AF04531RA)
• Piperidine (Sigma-Aldrich, cat. no. 822299)
• Phenol (Sigma-Aldrich, cat. no. W322340)
• Pyridine (Sigma-Aldrich, cat. no. 360570)

13. Equipment and Materials

• Mechanical overhead stirrer (IKA, mod. RW11)
• Disposable graduated polypropylene syringes (Sigma-Aldrich, cat. no. Z683531 (luer slip tip, centered, capacity 1 mL, graduated, 0.01 mL, non-sterile))
• Disposable graduated polypropylene syringes (Henke Sass Wolf, cat. no. 4050-X00V0 (luer lock tip, centered, capacity 5 mL, graduated, 0.2 mL, non-sterile) and cat. no. 4100-X00V0 (luer lock tip, centered, capacity 10 mL, graduated, 0.5 mL, non-sterile))
• Polyethylene Frits (Roland Vetter Laborbedarf OHG, cat. no. CEL-053 (average pore size 35 µm, diameter 12.4 mm, 3 mm thick) and cat. no. CEL-1016 (average pore size 35 µm, diameter 16.2 mm, 3 mm thick))
• Hypodermic injection needles (BD PrecisionGlide, cat. no. 300078 (0.8x30 mm))

• Disposable syringe filters (BioNaky, cod. no. FSPTFE1322 (13 mm – 22 μm PTFE membrane)
• Disposable graduated 15- and 50-mL polypropylene centrifuge tubes (with screw cap)
• Crimp headspace vials (6 mL clear borosilicate glass 22x38 mm, 20 mm beveled edge, flat bottom, short neck, cod. P0000032)
• Aluminum crimp caps (20 mm open-top aluminum crimp cap (10 mm hole) with 20 mm natural PTFE/ white silicone septa 3 mm thick, cod. C0000196)
• Adjustable standard biological pipettes (various sizes)
• Polypropylene centrifuge microtubes (OLEN, cat. no. K30-1020 (2.0 mL))
• Benchtop centrifuge (ThermoFisher Scientific, mod. Sorvall ST 8)
• Vortex mixer (Gehaka, mod. AV-2)
• Orbital shaker platform (SOLAB, mod. SL-180/DT)
• Digital ultra-thermostatic refrigerated bath circulator (SOLAB, mod. SL-152)
• Lyophilizer (Martin Christ Gefriertrocknungsanlagen GmbH, mod. Alpha 2-4 LSCbasic)
• Analytical RP-HPLC systems (Shimadzu Corp., Prominence-*i* LC-2030C 3D Plus and Prominence UFLC (SPD-M20A (PDA detector), SIL-10AF (autosampler), DGU-20A5R (degasser), LC-20AT (pump), and CBM-20A (system controller)); (column: Inertsil ODS-3V (250x4.6 mm, 5μm))
• HRMS-ESI-QTOF 6545
• UHPLC - Agilent Infinity II 1290
• NMR spectrometer Bruker Biospin Avance III operating at 11.4 T (600 MHz for ^{1}H, 150 MHz for ^{13}C) with a 5 mm TCI cryoprobe.
